# Whole-exome analysis reveals novel somatic genomic alterations associated with outcome in immunochemotherapy-treated diffuse large B-cell lymphoma

**DOI:** 10.1038/bcj.2015.69

**Published:** 2015-08-28

**Authors:** A J Novak, Y W Asmann, M J Maurer, C Wang, S L Slager, L S Hodge, M Manske, T Price-Troska, Z-Z Yang, M T Zimmermann, G S Nowakowski, S M Ansell, T E Witzig, E McPhail, R Ketterling, A L Feldman, A Dogan, B K Link, T M Habermann, J R Cerhan

**Affiliations:** 1 Division of Hematology, Mayo Clinic, Rochester, MN, USA; 2 Department of Health Sciences Research, Mayo Clinic, Jacksonville, FL, USA; 3 Division of Biomedical Statistics and Informatics, Mayo Clinic, Rochester, MN, USA; 4 Department of Laboratory Medicine and Pathology, Mayo Clinic, Rochester, MN, USA; 5 Departments of Pathology and Laboratory Medicine, Hematopathology Service, Memorial Sloan Kettering Cancer Center, New York, NY, USA; 6 Internal Medicine, University of Iowa, Iowa City, IA, USA; 7 Division of Epidemiology, Mayo Clinic, Rochester, MN, USA

## Abstract

Lack of remission or early relapse remains a major clinical issue in diffuse large B-cell lymphoma (DLBCL), with 30% of patients failing standard of care. Although clinical factors and molecular signatures can partially predict DLBCL outcome, additional information is needed to identify high-risk patients, particularly biologic factors that might ultimately be amenable to intervention. Using whole-exome sequencing data from 51 newly diagnosed and immunochemotherapy-treated DLBCL patients, we evaluated the association of somatic genomic alterations with patient outcome, defined as failure to achieve event-free survival at 24 months after diagnosis (EFS24). We identified 16 genes with mutations, 374 with copy number gains and 151 with copy number losses that were associated with failure to achieve EFS24 (*P*<0.05). Except for *FOXO1 and CIITA,* known driver mutations did not correlate with EFS24. Gene losses were localized to 6q21-6q24.2, and gains to 3q13.12-3q29, 11q23.1-11q23.3 and 19q13.12-19q13.43. Globally, the number of gains was highly associated with poor outcome (*P*=7.4 × 10^−12^) and when combined with *FOXO1* mutations identified 77% of cases that failed to achieve EFS24. One gene (*SLC22A16*) at 6q21, a doxorubicin transporter, was lost in 54% of EFS24 failures and our findings suggest it functions as a doxorubicin transporter in DLBCL cells.

## Introduction

Diffuse large B-cell lymphoma (DLBCL) is an aggressive non-Hodgkin lymphoma and the most common subtype in Western countries.^1, 2^ Factors that predict DLBCL prognosis include clinical characteristics,^3^ cell-of-origin (COO)^4, 5^ and *MYC* translocation status.^6, 7, 8^ Although the majority of DLBCL patients are potentially cured with the current standard of care that consists of rituximab (anti-CD20 monoclonal antibody) plus anthracycline-based chemotherapy (immunochemotherapy), most commonly given as rituximab, cyclophosphamide, doxorubicin, vincristine and prednisone (R-CHOP), lack of remission or early relapse remains a major clinical issue.^9^ We recently reported that ~70% of DLBCL patients who were treated with R-CHOP for curative intent and who did not have progression or relapse, retreatment or death within 24 months of diagnosis (termed as event-free survival or EFS24) had an 8% absolute risk of DLBCL relapse in the next 5 years and a subsequent overall survival equivalent to that of the age- and sex-matched general population (that is, a normal life expectancy).^10^ In contrast, of the ~30% of DLBCL patients who did not achieve EFS24, outcome was poor with a median survival of 13 months after a relapse or retreatment event. Although clinical factors such as age, sex, lactate dehydrogenase, extranodal sites, bulky disease, stage, performance status and international prognostic index score can partially predict EFS24,^11^ additional factors are needed to identify these high-risk patients, particularly biologic factors that might ultimately be amenable to intervention such as a pathway-targeted therapy.^9, 10^

Next-generation sequencing and related technologies are approaches to identify somatic mutations that contribute to lymphomagenesis (that is, drivers), predict prognosis, and identify novel therapeutic targets. For example, somatically acquired mutations or deletions in genes involved in B-cell receptor (BCR) signaling (*CD79,*^12^ *CARD11,*^13^ *BLIMP*^14, 15^), apoptosis (*PIM1,*^16^ *TNFAIP3,*^17^ *TRAF5*^(ref. 18)^) or inflammatory responses (*MYD88*^(ref. 19)^) have recently been shown to contribute to DLBCL pathogenesis. The biologic importance of these mutations, which result in constitutive activation of the BCR pathway and/or activation of nuclear factor-κB (NF-κB), have been validated by *in vitro* and clinical studies. Whole-exome sequencing (WES) has identified additional recurrent mutations in genes associated with DLBCL lymphomagenesis.^20, 21, 22^ Although providing important biologic insights, few of these studies have focused on identifying key genomic events that predict response to therapy or impact overall prognosis of DLBCL patients. Factors that predict DLBCL prognosis include COO^4^ and *MYC* translocation status.^6, 8^ For COO, the poorer prognosis of ABC-DLBCL is frequently associated with constitutive activity of the NF-κB pathway and mutations in upstream activators such as *MYD88,*^19^ *CARD11*^(ref. 13)^ and *CD79B.*^12^ However, there is conflicting data regarding its usefulness as a predictive biomarker for DLBCL clinical failure.^23^ *c-MYC* rearrangements or *MYC* ‘double hits' detected by fluorescence *in situ* hybridization are correlated with poor prognosis, but they occur in only 6–14%^(refs 6, 24)^ of DLBCL cases.

In an exploratory study, we evaluated associations between whole-exome data from 51 immunochemotherapy-treated DLBCL patients with clinical and outcome data, focusing on identifying patients who fail to achieve EFS24. We report the association of copy number alterations (CNAs) and somatic mutations with EFS24 and our results highlight a potential role for novel gene mutations and CNAs on chromosomes 3q13.12-3q29, 6q21, 11q23.1-11q23.3 and 19q13.12-19q13.43 in DLBCL prognosis. From one of the top regions, 6q21, we also identify and biologically validate the potential impact of deletions in *SLC22A16,* a doxorubicin transporter.

## Materials and methods

### Patients

This study was reviewed and approved by the human subjects review board of Mayo Clinic and the University of Iowa, and written informed consent was obtained from all participants. Since 2002, all newly diagnosed lymphoma patients have been prospectively offered enrollment into the Molecular Epidemiology Resource of the University of Iowa/Mayo Clinic Specialized Program of Research Excellence.^25^ Patients with primary mediastinal or testicular DLBCL, patients who did not receive full immunochemotherapy due to toxicity or comorbidities and patients in remission who died of non-lymphoma causes within 24 months of their diagnosis (that is, deaths unrelated to DLBCL or its treatment) were excluded from this analysis.

### Whole-exome sequencing

WES of DNA from 51 newly diagnosed frozen DLBCL tumors and paired blood samples was performed at the Broad Institute as previously described.^22^ Tumor purity ranged from 40 to 90% based on pathologic review of hematoxylin-and-eosin-stained slides. All available paired samples (*n*=43) were also genotyped using the Affymetrix 6.0 SNP array (Affymetrix, Santa Clara, CA, USA). A full description of the WES and CNA analysis is described in Supplementary Methods.

### Determination of COO and double hit lymphoma status

COO was determined using gene expression profile data (*n*=34)^26^ or the Hans algorithm (*n*=16).^27^ All available cases (*n*=45) were screened for a *MYC* rearrangement, methods and fluorescence *in situ* hybridization interpretation are shown in (Supplementary Table 1).

### Statistical analysis

EFS24 was based on disease status 24 months after diagnosis^10^—achieving EFS24 was defined as being alive without progression, relapse or unplanned retreatment following immunochemotherapy 24 months after diagnosis. We estimate measures of association using odds ratios and report the association of genomic variants with EFS24 using a *χ*^2^ test. For this exploratory study, we did not adjust for multiple testing. Outcomes on identified regions of interest were also presented via continuous time-to-progression (TTP) curves by mutation or CNA status using the method of Kaplan–Meier and log-rank tests.

### SLC22A16 mRNA expression and sequencing

Quantitative PCR for *SLC22A16* was performed using the CFX96 Real Time System (Bio-Rad, Hercules, CA, USA). cDNA was synthesized using Super-Script III First Strand (Life Technologies, Carlsbad, CA, USA). All primer/probes were synthesized by Integrated DNA Technologies, Inc. (Coralville, IA, USA). Each sample was run in triplicate and ΔΔ*C*_t_ analysis was performed to quantitate SLC22A16 expression using GAPDH as an internal standard.^28^ For Sanger sequencing, DNA was extracted from DLBCL biopsies using Puregene (Qiagen, Germantown, MD, USA), used for PCR amplification and sequenced at the Mayo Clinic Genomics Research Core.

### Generation of the OCI-Ly7-SLC22A16 cell line

SLC22A16 mRNA was amplified by PCR with the addition of a 5′ HA tag and cloned into the pLEX-MCS vector (ThermoScientific, Lafayette, CO, USA). OCI-Ly7, OCI-Ly3 and SUDHL4 cells were transduced with 1 × 10^6^ million infectious units of each virus, puromycin was added, and cells were maintained in selection medium. To confirm protein expression, 1 × 10^6^ million cells were lysed, supernatants were collected and immunoprecipitated with anti-HA beads (Pierce, Rockford, IL, USA) and analyzed by western blot analysis.

### Doxorubicin uptake assay

OCI-LY7 cells expressing SLC22A16 and vector control were incubated with increasing concentrations of [14-^14^C]doxorubicin hydrochloride (PerkinElmer, Waltham, MA, USA) in a medium containing 100 mM NaCl, 2 mM KCl, 1 mM CaCl_2_, 1 mM MgCl_2_ and 10 mM HEPES, pH 7.5, for 60 min at 37 °C and uptake was terminated with ice-cold phosphate-buffered saline. Samples were lysed in 1% Triton X-100 (Sigma, St Louis, MO, USA), and radioactivity was counted using a MicroBeta TriLux (PerkinElmer).

### Proliferation assay

OCI-LY7, OCI-Ly3 and SUDHL4 cells expressing SLC22A16 and vector control were cultured in 0.5% bovine serum albumin, Iscove's modified Dulbecco's medium and doxorubicin hydrochloride (Sigma) was added at the indicated concentrations in triplicate. After 24 h, cultures were pulsed with 0.05 mCi tritiated thymidine (PerkinElmer) for 18 h. ^3^H-TdR incorporation levels were determined using a MicroBeta TriLux.

## Results

### Patient characteristics

Of the 51 immunochemotherapy-treated DLBCL patients in the analysis (Table 1, R-CHOP *n*=43, rituxan-etoposide, prednisone, oncovin, cytoxan, hydroxyrubicin *n*=5, epratuzumab, rituxan-cytoxan, hydroxyrubicin, oncovin, prednisone *n*=2, R-ProMACE CytaBOM *n*=1), 13 (25%) patients did not achieve EFS24, whereas 38 (75%) were event free at 24 months, which is consistent with the full Specialized Program of Research Excellence DLBCL cohort and other cohorts.^10^ Based on a median follow-up of 48 months (range, 38–59 months), 77% of patients not achieving EFS24 had died compared with only 5% of patients achieving EFS24. Compared with patients achieving EFS24, those who did not achieve EFS24 were older, but similar in Ann Arbor stage, performance status, B-symptoms and tumor molecular characteristics (Table 1).

### Copy number alterations associated with failure to achieve EFS24

CNAs are typically defined as genomic structural changes with either less or more than two copies of DNA,^29^ and are known to be important in DLBCL.^30^ The exon-level CNAs identified by patternCNV showed excellent concordance with the CNAs identified using the Affymetrix SNP Chip 6.0 from the same tumor sample (Supplementary Figure 1). PatternCNV analysis of individual patients is shown in Supplementary Figure 2 and a summary of the number of samples with a CNA called by chromosomal position is shown in Supplementary Figure 3.

**Figure 1:**
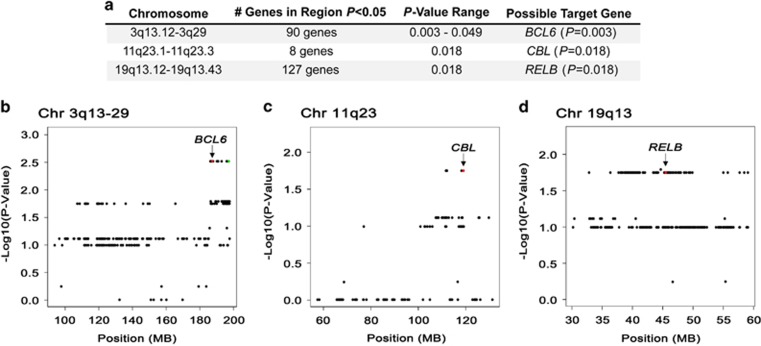
CNA gains associated with failure to achieve EFS24. (**a**) Regions of chromosome gain, number of genes with *P*<0.05 and potential target genes in the regions. Graphical representation of individual genes (represented by a dot) plotted by chromosomal position and –Log10(*P*-value) on chromosomes (**b**) 3q13.12-3q29, (**c**) 11q23.1-11q23.3 and (**d**) 19q13.12-19q13.43. The position of *BCL6*, *CBL* and *RELB* is highlighted on individual graphs. Statistical analysis is described in the Materials and methods section.

In the gene-level copy number gain analysis, we identified 374 genes that had evidence of an association with a failure to achieve EFS24 at *P*<0.05, all with odds ratios >5.0 (Supplementary Table 2). Although micro-alterations were detected, most of the genes clustered into eight regions of chromosomal gains. The regions with the strongest association with failure to achieve EFS24 included 3q13.12-3q29, 11q23.1-11q23.3 and 19q13.12-19q13.43 (Figure 1a). On chromosome 3q, 90 gene gains were identified, with a cluster of genes close to *BCL6* having *P*-values of 0.003 (Figure 1b). On chromosome 11q, eight gene gains were identified, with a cluster of genes proximal to *CBL* having *P*-values of 0.018 (Figure 1c). On chromosome 19q, 127 gene gains were identified, with a multiple genes near *RELB* having *P*-values of 0.018 (Figure 1d).

**Figure 2:**
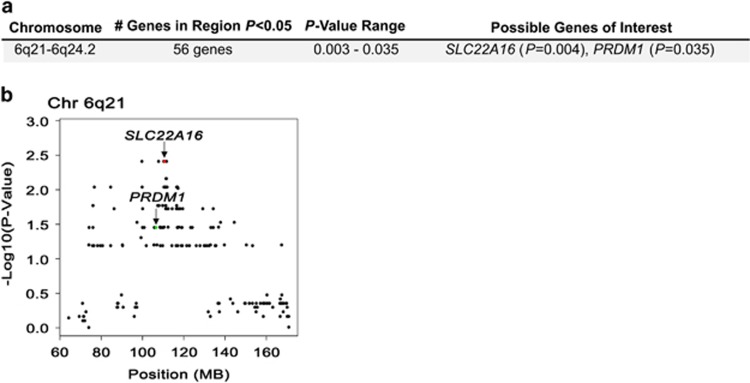
CNA losses associated with failure to achieve EFS24. (**a**) Regions of chromosome loss, number of genes with *P*<0.05 and potential target genes in the regions. (**b**) Graphical representation of individual genes (represented by a dot) plotted by chromosomal position and –Log10(*P*-value) on chromosome 6q21-6q24.2. The position of *SLC22A16* and *PRDM1* is highlighted on the graphs. Statistical analysis is described in the Materials and methods section.

In the gene-level copy number loss analysis, we identified 151 genes in eight regions that were associated with failure to achieve EFS24 at *P*<0.05, all with odds ratios >50 (Supplementary Table 3); 56 of these genes were located on chromosomes 6q21-6q24.2 (Figure 2a), with a cluster of genes proximal to *SLC22A16* having a *P*-value of 0.004 (Figure 2b). The two genes from this region most closely associated with 6q deletion in lymphoma are *PRDM1* and *TNFAIP3*, but only *PRDM1* was associated with failure to achieve EFS24 (*P*=0.04).

### Coding single nucleotide variants associated with failure to achieve EFS24

In previous work,^22^ we identified multiple somatic mutations enriched in DLBCL, and together with other publications,^20, 21^ there is now a fairly comprehensive list of common somatic variants (potential ‘drivers') in DLBCL. In an analysis of previously identified mutations,^20, 21, 22^ none were associated with failure to achieve EFS24 (Supplementary Table 4). Although, *CIITA* (*P*=0.08) and *FOXO1* (*P*=0.10) showed a suggestive association with failure to achieve EFS24. *FOXO1* is consistent with other work showing that mutations in this gene are associated with decreased overall survival in DLBCL patients.^31^ *CIITA*, previously shown to be a recurrent gene fusion partner in lymphoid cancers,^32^ is a master regulator of MHC class II expression and loss of its function may contribute to escape from immunosurveillance and poor outcome.

In our analysis of novel variants, we identified 16 genes for which the presence of somatic mutations predicted failure to achieve EFS24 at *P*<0.05, all odds ratios >7.0 (Table 2). The mutations were rarely observed in patients achieving EFS24 (0–8%), whereas they were observed in 20–30% of patients who failed to achieve EFS24. The cytogenetic band of the gene position, whether or not the gene is located within a CNA region, and the gene description and function are described in Table 2.

### Genetic signature associated with EFS24 and TTP

**Figure 3:**
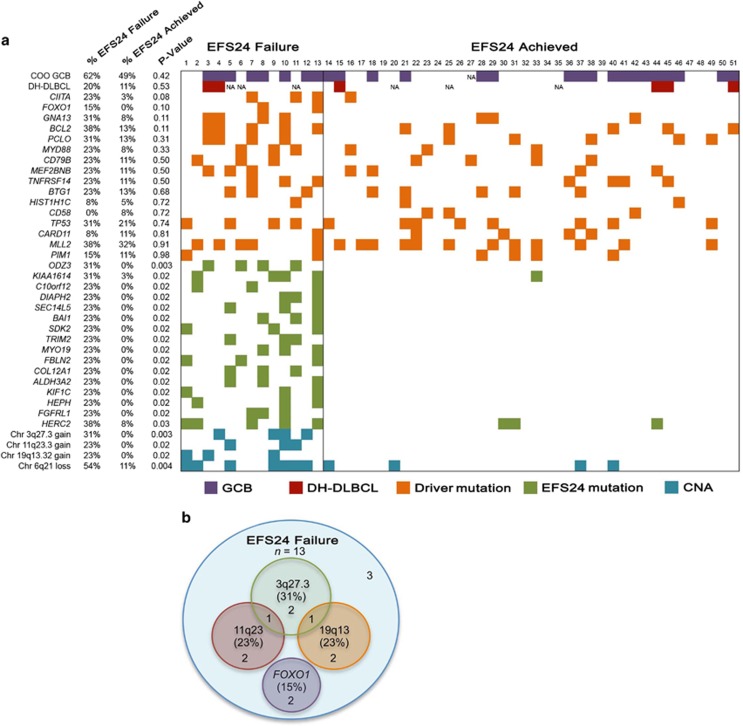
Genetic signature associated with failure to achieve EFS24. (**a**) Molecular features including COO, *MYC*-double hit (DH) status, the presence of driver mutations or mutations and CNAs associated with EFS24 failure are shown in individual patients. (**b**) Venn diagram showing integration of the EFS24 CNA and mutation data reveal that 77% patients who fail to achieve EFS24 can be identified using a combination of four variants (*FOXO1* mutation and gains in 3q27.3 (*BCL6*), 11q23.3 (*CBL*) and 19q13.32 (*RELB*)).

**Figure 4:**
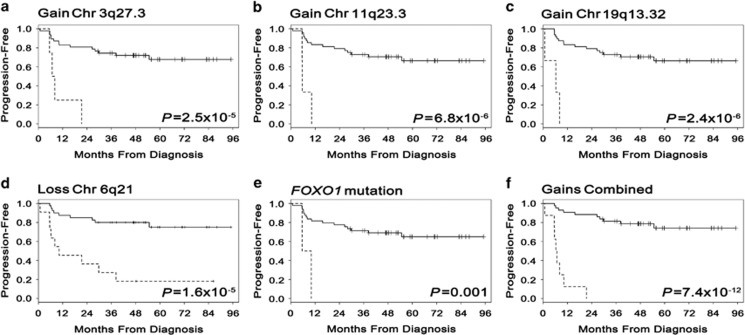
Association of CNAs and mutations with DLBCL time to progression. Kaplan–Meier curves for the association of DLBCL time to progression with gains in (**a**) Chr 3q27.3 (*BCL6*, *n*=4), (**b**) 11q23.3 (*CBL*, *n*=3), (**c**) 19q13.32 (*RELB*, *n*=3), loss in (**d**) 6q21 (*SLC22A16*, *n*=11), a mutation in (**e**) *FOXO1* (*n*=2) or (**f**) a combined analysis of the three gains.

A full summary of individual patient molecular features (COO, *MYC*-double hit), mutations and CNAs are summarized for the 51 cases in Figure 3a and highlight a novel and unique genetic signature of patients who fail to achieve EFS24. Integration of the CNA and mutation status that were specific to EFS24 failures revealed that 77% of patients who fail to achieve EFS24 have a combination of four variants (*FOXO1* mutation and gains in 3q27.3, 11q23.3 and 19q13.32; Figure 3b). Kaplan–Meier curves for the association of these variants with TTP are shown in Figures 4a and e. Combined analysis of the three gains (Figure 4f) was highly associated with TTP. The strong association of CNAs with outcome suggests an important role for DNA genomic stability. Indeed, patients who failed to achieve EFS24 had a greater total number of genes gained and lost compared with cases who achieved EFS24 (*P*=0.004; Supplementary Figure 4), supporting the hypothesis of greater genomic instability in patients with poor prognosis disease.

### A role for SLC22A16 in the transport of doxorubicin in DLBCL

The most common genetic change associated with failure to achieve EFS24 was a deletion in genes located at 6q21 (54% of cases). Genes in this region with *P*=0.004 include *SOBP*, *GPR6*, *METTL24*, *DDO*, *SLC22A16* and *REV3L*. Of these, only *SLC22A16* had a potentially damaging mutation (R150Q) detected by WES and suggestive biologic relevance for DLBCL. A detailed description of CNA loss in *SLC22A16* by exon is shown in Supplementary Figure 5A, a gene-level CNA loss was called if two or more exons were lost by patternCNV analysis. Sanger sequencing and DNA quantitative PCR of SLC22A16 to detect a CNA loss were performed in an additional 43 cases of DLBCL (patient characteristics shown in Supplementary Figure 5B) and revealed two additional mutations, L325R and H49Y, both of which were in cases that failed to achieve EFS24. Of the 43 cases, we detected a CNA loss in 7; however, we did not find an association with EFS24 failure. This may be due in part to the fact that we found that DNA quantitative PCR is not as sensitive at calling CNAs as WES and Affymetrix SNP Chip, likely due to mixed tumor purity. A full summary of all the cases with a genetic variant in *SLC22A16* (CNA and single nucleotide variant), the method used to identify the CNA and single nucleotide variant, and the EFS24 status of each case is shown in Supplementary Figure 5C.

**Figure 5:**
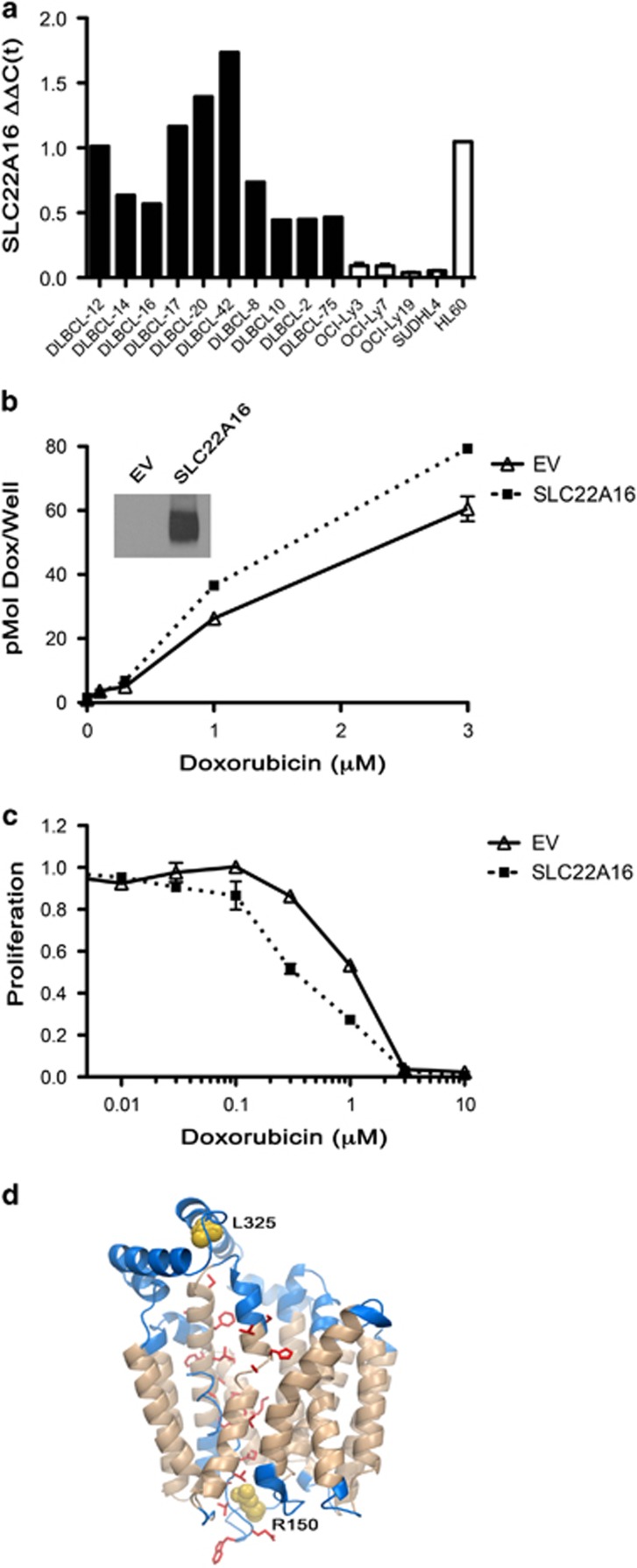
Expression and functional characterization of SLC22A16 in DLBCL cells. (**a**) Expression of SLC22A16 mRNA levels was measured by quantitative real-time PCR in a panel of DLBCL tumors (*n*=10) and cell lines (*n*=5) as described in Materials and methods. (**b**) Analysis of OCI-LY7 cells expressing an empty vector control (EV) or HA-SLC22A16 was performed by western blot analysis with an anti-HA-specific antibody to confirm SLC22A16 expression (inset). Doxorubicin uptake was measured in OCI-LY7 EV (solid line, open triangle) or SLC22A16 (dashed line, square symbol) cells using ^14^C-doxorubicin as described in Materials and methods. The experiment shown is representative of three independent experiments and error bars for triplicate wells are shown. (**c**) Proliferation of OCI-LY7 EV (solid line) or SLC22A16 (dashed line) cells was measured in the presence of increasing doses of doxorubicin (0–10 μM) by ^3^H-TdR incorporation. Data from each cell line were normalized to its respective nil control and the experiment shown is representative of three independent experiments, error bars for triplicate wells are shown. (**d**) Bioinformatic modeling of SLC22A16 mutations shows their location in major facilitator superfamily domains, which were assigned by Pfam using a hidden Markov model. Tan regions are predicted to be trans-membrane helices and red residues (side chains shown) are the predicted pore. Each mutation site sits at the entrance/exit of the predicted translocation pore (yellow spheres).

*SLC22A16* is an organic cation transporter that has been shown to transport chemotherapeutic drugs including doxorubicin, a central component of DLBCL immunochemotherapy. Successful drug response has been correlated with the level of activity and expression of this transporter in tumor cells, but its role in DLBCL remains undefined.^33, 34^ We first measured SLC22A16 mRNA by quantitative real-time PCR in a panel of DLBCL tumors and cell lines and found that it was expressed at variable levels, with undetectable levels seen in DLBCL cell lines; HL60 cells served as a positive control (Figure 5a). To determine whether expression of SLC22A16 would have an impact on doxorubicin transport, we overexpressed either a control or a HA-tagged SLC22A16 in OCI-Ly7 DLBCL cells, which do not express endogenous SLC22A16 (Figure 5a). SLC22A16 expression was confirmed by western blot analysis (Figure 5b, inset). In a doxorubicin uptake assay, we found that OCI-Ly7 cells expressing SLC22A16 consistently had more ^14^C-doxorubicin uptake (25% increase at the 3 μM dose; Figure 5b) and are more sensitive to doxorubicin-induced growth inhibition across a wide dose range (0.3–1 μM) when compared with control cells (Figure 5c). Although the SLC22A16-OCI-Ly7 cells did not have a statistically significant increase in ^14^C-doxorubicin uptake or inhibition of proliferation, the results were consistent over three experiments, and were similar to SLC22A16 in overexpression models.^33, 34^ Furthermore, OCI-Ly3 and SUDHL4 DLBCL cell lines showed a similar proliferation response upon SLC22A16 overexpresssion (Supplementary Figure 6). Owing to the very low level of endogenous *SLC22A16* expression in DLBCL cell lines, we were unable to assess the impact of its knockdown on ^14^C-doxorubicin uptake.

As previously mentioned, mutations in *SLC22A16* (L325R, R150Q, H49Y) were identified and modeling of L325R and R150Q mutations shows that both mutations sit at the entrance/exit of the predicted translocation pore, suggesting a possible impact on function and doxorubicin transport, H49Y was not predicted to have an impact (Figure 5d). However, the functional significance of the mutations remains unexplored as mutations in *SLC22A16* are rare compared with CNA loss. Taken, together these data suggest SLC22A16 can transport ^14^C-doxorubicin in DLBCL cells and that loss of SLC22A16 through gene deletion or mutation could impact the sensitivity of DLBCL cells toward doxorubicin-containing therapies.

## Discussion

We have identified somatic genomic alterations associated with disease-related outcome in immunochemotherapy-treated DLBCL patients, defined here as failure to achieve EFS24. This is a sentinel time point for DLBCL patients, as immunochemotherapy-treated patients achieving EFS24 have a low probability of relapse and an overall survival rate equivalent to the background population, whereas patients who fail to achieve EFS24 have a very poor prognosis and are a group that should be considered for alternative and novel therapies. In our gene-level analysis of CNAs and mutations, four chromosomal regions and a panel of 16 genes were identified that correlated with failure to achieve EFS24. Furthermore, for loss of 6q21, one of our strongest findings, we characterized the potential role of *SLC22A16* from this region, showing that loss of its expression may contribute to doxorubicin resistance, one of the key chemotherapeutic agents in R-CHOP. Finally, none of the published driver mutations,^20, 21, 22^ with the possible exception of *CTIIA* and *FOXO1*, were found to be associated with failure to achieve EFS24, suggesting that the somatic genomic alterations in the etiology of DLBCL may be different from those important in response to therapy and prognosis.

High-resolution array comparative genomic hybridization, fluorescence *in situ* hybridization and quantitative PCR of DLBCL tumors have been previously performed to assess the significance of CNAs in DLBCL, many of which focused on the correlation of CNAs with COO. Germinal center B-cell (GCB)-DLBCL is characterized by frequent amplifications in *REL* and *BCL2* translocations.^35, 36^ Consistent with these studies, we also found that the *BCL2/IGH* fusion was restricted to GCB-DLBCL (Supplementary Table 1), whereas gains in *REL* trended toward GCB-DLBCL (70% of *REL* gains were in GCB cases, data not shown). Our results for 6q21 and 3q27 (Figures 1 and 2) are also consistent with those of Bea *et al.*,^37^ who studied 224 DLBCL tumors by array comparative genomic hybridization and reported loss of 6q21-q22 in 25% of tumors and gain/amplifications of 3q27-qter in 16% of tumors. Both 6q21-q22 and chromosome 3 gains were found more frequently in ABC-DLBCL,^37^ whereas we found fairly equal distribution across COO subtype (Figure 3).

Previous correlations between CNAs and clinical outcome in DLBCL highlight a role for gains in 3q27 and 18q, as well as loss of 6q21. In a study of 64 newly diagnosed DLBCL treated with anthracycline-based therapies, Chen *et al.* reported that loss of 6q and gain of 3q were associated with shorter time to treatment failure.^38^ Gains of 3q25-q27 were also found to correlate shorter patient survival and elevated *BCL6* expression.^37^ Gain of *BCL6*, which occurred in 8% of our cases, was identified as one of our top hits from 3q27.3, and was associated with failure to achieved EFS24 (*P*=0.003). It will be of future interest to determine if 3q27.3 gains correlate with *BCL6* expression and outcome in a larger cohort. Another study showed that shorter cause-specific overall survival of DLBCL at 5 years correlated with gains in 18q gains.^39^ Although we identified gene copy number gains in18q, including *MALT1* at 18q21.32 (20% of cases) and *BCL2* at 18q21.33 (32% of cases), none of the genes in this region were associated with failure to achieve EFS24 (data not shown). The discrepancy in our results may be partially due to the use of different end points, how the CNAs were defined, the fact that 18q showed the highest correlation with outcome when the analysis was restricted to nodal disease,^39^ or our small sample size. In an analysis of 180 DLBCLs, Monti and colleagues used high-density SNP arrays to identify CNAs in DLBCL and reported that alteration of the p53 pathway and cell cycle genes predicted overall survival.^40^ Patients with ‘complex tumors' or those who had multiple alterations in p53/cell cycle genes had decreased overall survival. Of the 14 genes used to define ‘complex tumors' for that study, we found that all 14 genes had one or more alterations in our tumors, but only *MDM2*, which had a CNA gain in 12% of cases, was associated with failure to achieve EFS24 (*P*=0.049). Because of low frequency of many of the variants and the small sample of this study, we were not able to conduct a multi-gene analysis. However, our finding that there was a strong association of the total number of genes gained and lost with failure to achieve EFS24 (Supplementary Figure 2) provides support for the role of complex genomes/genomic instability with poor outcome in DLBCL.

Genomic alterations at 11q23 and 19q13 have been previously identified, but are less well characterized in DLBCL. Gains of 11q23-q24 were identified in CD5^+^ DLBCL,^41^ and follow-up studies highlighted the potential role for *SIK2* amplification. Although *SIK2* was not identified as one of the top 11q23 candidate genes for EFS24 in our study, we did identify alterations in this gene in 8% of cases, and there was a suggestive association of gains in *SIK2* with failure to achieve EFS24 (*P*=0.07). We identified a cluster of seven genes on 19q13 (*ALG9*, *DLAT*, *PIH1D2*, *C11orf57*, *PTS*, *UBE4A* and *CBL*) that were associated with failure to achieve EFS24. Of these, only *CBL*, a known proto-oncogene, has been shown to have a role in hematopoietic malignancies. The Cbl adaptor protein has been studied intensively in many cell types and up to 150 proteins are affected or regulated by Cbl proteins or the Cbl interactome.^42^ Depending on the specific environment, Cbl can act as a scaffolding protein, an E3 ubiquitin protein ligase, or negatively regulate kinase activity. Although we have no direct evidence that Cbl is overexpressed in tumors that carry the 11q23 gain, one can envision a scenario where dysregulation of the Cbl interactome could have significant impact on pathway activation in lymphoma. Gain of 19q13 involved a large number of genes that were associated with failure to achieve EFS24, making it challenging to identify specific gene(s). *RELB*, central to non-canonical NF-κB activation, may be an obvious candidate and has been shown to be activated by BAFF-R, lymphotoxin-β and CD40 ligand, all of which have been shown to activate B cells and contribute to lymphomagenesis.^43, 44, 45^ *RELB* can also heterodimerize with p50 upon canonical NF-κB activation, which has a significant role in lymphoma.^46^

In addition to CNAs, our analysis highlights the potential importance of novel mutations in disease progression. Of the genes identified, none have been previously shown to have a significant role in lymphomagenesis and based on their gene ontogeny annotations, many of the genes are important in metabolism and actin/cell adhesion regulation. Furthermore, 14 of the 16 genes are also located in a region of a CNA suggesting the potential for additional mechanisms of dysregulation (Table 2). *ALDH2A2* belongs to the aldehyde dehydrogenase family, expression of which is thought to contribute to drug resistance and poor response to antitumoral drugs. In particular, ALDH is responsible for tumor resistance against alkylating agents.^47, 48^ *DIAPH2, SDK2, MYO19, FBLN2* and *KIF3Ca* all have a role in cell adhesion or regulation of microtubules and the actin cytoskeleton. Recent studies highlight an important role for structural proteins in regulation of BCR clustering and internalization, and disruption of these pathways can lead to chronic BCR activation, which is characteristic of ABC-DLBCL.^49, 50^

Recent work has highlighted the significance of mutations in *TP53*,^51, 52^ *FOXO1*^(ref. 31)^ and *MYD88*^(ref. 53)^ with inferior DLBCL outcomes. Our analysis of these genes (Supplementary Table 4) complement this work, mutations in *FOXO1* were more prevalent in cases that failed to achieve EFS24 (15% vs 0%) and *FOXO1* mutations correlated with TTP (Figure 4e). Similarly, mutations in *MYD88* were found in 23% of EFS24 failure vs 8% of those who achieved EFS24. In a recent of analysis of *TP53* mutations in 506 *de novo* R-CHOP-treated DLBCL, 21.9% had mutations in *TP53* and the presence of a mutation was associated with both overall survival and progression-free survival. *TP53* mutations were found in 24% of our cases, however, there was not a strong correlation with outcome (*P*=0.57, 31% of EFS24 failures and 21% of those that achieved EFS24). Our use of EFS24 as an end point and sample size may partially explain this discrepancy. Our data also complement previous studies on the association of mutations with COO. In our patients, the GCB-associated mutations in *TNFRSF14*^(ref. 20)^ were restricted to GCB-DLBCL cases and the ABC-associated mutations in *MYD88*^(ref. 20)^ were restricted to non-GCB/ABC-DLBCL tumors (Figure 3).

A limitation of our exploratory study is the lack of validation of our findings in an independent cohort and the lack of correction for multiple testing. Thus, it is possible that our results may be false positives. Although our provocactive novel targets are supported by prior work, they will require replication and functional validation.

In summary, we provide the first data using WES to identify somatic genomic alterations in immunochemotherapy-treated DLBCL tumors that are associated with failure to achieve EFS24, identifying those patients who have treatment-resistant and aggressive disease. Although these novel targets require replication and functional validation, the results are provocative and supported by prior work. We biologically validate the potential impact of deletions in *SLC22A16* at 6q21 and future biological analysis of additional CNAs and mutations is warranted.

## Figures and Tables

**Table 1:** DLBCL patient characteristics

*Characteristic*	*Patients achieving EFS24 (*N=*38)*	*Failure to achieve EFS24 (*N=*13)*	P*-value*
Diagnosis age, median (range), years; IQR	62 (26–82); 55–70	67 (57–82); 62–74	
Age > 60 years	20 (52%)	11 (85%)	0.042
Male	25 (66%)	7 (54%)	0.44
PS 2+	5 (13%)	2 (15%)	0.84
Ann Arbor Stage III-IV	26 (68%)	9 (69%)	0.96
2+ Extranodal sites group	4 (11%)	3 (23%)	0.26
LDH >ULN	24 (63%)	10 (83%)	0.19
			
*IPI*			
0–1	11 (29%)	2 (15%)	0.35
2	13 (34%)	3 (23%)	
3	11 (29%)	5 (38%)	
4 or 5	3 (8%)	3 (23%)	
B Symptoms	12 (32%)	4 (31%)	0.96
Bulky disease	6 (16%)	3 (23%)	0.55
			
*Follow-up^a^*			
Death	2 (5%)	10 (77%)	NA
Event	6 (16%)	13 (100%)	NA
			
*COO (n=50)*			
GCB	18 (49%)	8 (61%)	0.42
*MYC*-double hit (*n*=45)	4 (11%)	2 (20%)	0.53

Treatment: R-CHOP *n*=43, R-EPOCH *n*=5, ER-CHOP *n*=2, R-ProMACE CytaBOM *n*=1.

Abbreviations: COO, cell of origin; DLBCL, diffuse large B-cell lymphoma; EFS24, event-free survival at 24 months after diagnosis; GCB, germinal center B-cell; IPI, international prognostic index; IQR, interquartile range; LDH, lactate dehydrogenase; NA, not applicable; PS, performance status; ULN, upper limit of normal.

a FU Months Alive Cases, median (range); IQR: 48 (29–97); (38–59).

**Table 2:** Mutations associated with failure to achieve EFS24

*Gene*	*Overall number of patients with mutation*	*Patients achieving EFS24 (*N=*38)*		*Failure to achieve EFS24 (*N=*13)*		P*-value*	*Cytogenetic band*	*In region of CNA*	*Human genome compendium description*	*GO annotation/function*
		N *with mutation*	*% With mutation*	N *with mutation*	*% With mutation*					
*ODZ3/TENM3*	4	0	0	4	31	0.003	5q34	Yes	Tenascin family	Protein heterodimerization and homodimerization activity
*KIAA1614*	5	1	2.6	4	31	0.02	1q25.3	Yes	Reported gene with no known function	–
*C10orf12*	3	0	0	3	23	0.02	10q24.1	Yes	Reported gene with no known function	–
*DIAPH2*	3	0	0	3	23	0.02	Xq21.33	No	Formin homology family	Rho GTPase binding and actin binding
*SEC14L5*	3	0	0	3	23	0.02	16p13.3	Yes	Reported gene with no known function	–
*BAI1*	3	0	0	3	23	0.02	8q24.3	Yes	G-protein-coupled receptor	G-protein-coupled receptor activity
*SDK2*	3	0	0	3	23	0.02	17q25.1	Yes	Immunoglobulin superfamily	Cell adhesion
*TRIM2*	3	0	0	3	23	0.02	4q31.1	Yes	Tripartitie motif family	Ubiquitin-protein ligase activity and zinc ion binding
*MYO19*	3	0	0	3	23	0.02	17q12	Yes	Myosin	Motor activity and actin binding
*FBLN2*	3	0	0	3	23	0.02	3p25.1	Yes	Fibulin family	Extracellular matrix binding and calcium ion binding
*COL12A1*	3	0	0	3	23	0.02	6q13	Yes	Alpha chain of type XII collagen	Stuctural molecule activity
*ALDH3A2*	3	0	0	3	23	0.02	17p11.2	Yes	Aldehyde dehydrogenase family	Long-chain-alcohol oxidase and aldehyde dehydrogenase (NAD)
*KIF1C*	3	0	0	3	23	0.02	17p13.2	Yes	Kinesin family	Motor activity and microtubule motor activity
*HEPH*	3	0	0	3	23	0.02	11q12	No	Multicopper oxidase protein family	Oxidoreductase activity and copper ion binding
*FGFRL1*	3	0	0	3	23	0.02	4p16	Yes	Fibroblast growth factor receptor	Heparin binding and fibroblast growth factor-activated receptor activity
*HERC2*	8	3	7.9	5	38	0.03	15q13	Yes	HERC gene family	Ubiquitin-protein ligase activity and heme binding

Abbreviations: CNA, copy number alteration; EFS24, event-free survival at 24 months after diagnosis; GO, gene ontogeny.

## References

[bib1] Howlader NNA Krapcho M Garshell J Neyman N Altekruse SF Kosary CL(eds). SEER Cancer Statistics Review, 1975–2010 National Cancer Institute: Bethesda, MDAvailable from: : http://seer.cancer.gov/csr/1975_2010/ (last accessed November 2012). SEER data submission, posted to the SEER website, April2013

[bib2] Sant M Allemani C Tereanu C De Angelis R Capocaccia R Visser O Incidence of hematologic malignancies in Europe by morphologic subtype: results of the HAEMACARE project Blood 2010 116 3724 3734 20664057 10.1182/blood-2010-05-282632

[bib3] A predictive model for aggressive non-Hodgkin's lymphoma The International Non-Hodgkin's Lymphoma Prognostic Factors Project N Engl J Med 1993 329 987 994 8141877 10.1056/NEJM199309303291402

[bib4] Alizadeh AA Eisen MB Davis RE Ma C Lossos IS Rosenwald A Distinct types of diffuse large B-cell lymphoma identified by gene expression profiling Nature 2000 403 503 511 10676951 10.1038/35000501

[bib5] Wright G Tan B Rosenwald A Hurt EH Wiestner A Staudt LM A gene expression-based method to diagnose clinically distinct subgroups of diffuse large B cell lymphoma Proc Natl Acad Sci USA 2003 100 9991 9996 12900505 10.1073/pnas.1732008100 PMC187912

[bib6] Barrans S Crouch S Smith A Turner K Owen R Patmore R Rearrangement of MYC is associated with poor prognosis in patients with diffuse large B-cell lymphoma treated in the era of rituximab J Clin Oncol 2010 28 3360 3365 20498406 10.1200/JCO.2009.26.3947

[bib7] Rimsza LM Leblanc ML Unger JM Miller TP Grogan TM Persky DO Gene expression predicts overall survival in paraffin-embedded tissues of diffuse large B-cell lymphoma treated with R-CHOP Blood 2008 112 3425 3433 18544678 10.1182/blood-2008-02-137372 PMC4467875

[bib8] Savage KJ Johnson NA Ben-Neriah S Connors JM Sehn LH Farinha P MYC gene rearrangements are associated with a poor prognosis in diffuse large B-cell lymphoma patients treated with R-CHOP chemotherapy Blood 2009 114 3533 3537 19704118 10.1182/blood-2009-05-220095

[bib9] Sehn LH Early detection of patients with poor risk diffuse large B-cell lymphoma Leuk Lymphoma 2009 50 1744 1747 19860615 10.3109/10428190903308064

[bib10] Maurer MJ Ghesquieres H Jais JP Witzig TE Haioun C Thompson CA Event-free survival at 24 months is a robust end point for disease-related outcome in diffuse large B-cell lymphoma treated with immunochemotherapy J Clin Oncol 2014 32 1066 1073 24550425 10.1200/JCO.2013.51.5866 PMC3965261

[bib11] Maurer MJ Ghesquieres H Jais J-P Witzig TE Haioun C Thompson CA IPI24: an international study to create an IPI for the event-free survival at 24 months (EFS24) endpoint for DLBCL in the immunochemotherapy era Blood 2013 122 362 362

[bib12] Davis RE Ngo VN Lenz G Tolar P Young RM Romesser PB Chronic active B-cell-receptor signalling in diffuse large B-cell lymphoma Nature 2011 463 88 92 20054396 10.1038/nature08638 PMC2845535

[bib13] Lenz G Davis RE Ngo VN Lam L George TC Wright GW Oncogenic CARD11 mutations in human diffuse large B cell lymphoma Science 2008 319 1676 1679 18323416 10.1126/science.1153629

[bib14] Mandelbaum J Bhagat G Tang H Mo T Brahmachary M Shen Q BLIMP1 is a tumor suppressor gene frequently disrupted in activated B cell-like diffuse large B cell lymphoma Cancer Cell 2010 18 568 579 21156281 10.1016/j.ccr.2010.10.030 PMC3030476

[bib15] Calado DP Zhang B Srinivasan L Sasaki Y Seagal J Unitt C Constitutive canonical NF-kappaB activation cooperates with disruption of BLIMP1 in the pathogenesis of activated B cell-like diffuse large cell lymphoma Cancer Cell 2010 18 580 589 21156282 10.1016/j.ccr.2010.11.024 PMC3018685

[bib16] Gaidano G Pasqualucci L Capello D Berra E Deambrogi C Rossi D Aberrant somatic hypermutation in multiple subtypes of AIDS-associated non-Hodgkin lymphoma Blood 2003 102 1833 1841 12714522 10.1182/blood-2002-11-3606

[bib17] Novak U Rinaldi A Kwee I Nandula SV Rancoita PM Compagno M The NF-{kappa}B negative regulator TNFAIP3 (A20) is inactivated by somatic mutations and genomic deletions in marginal zone lymphomas Blood 2009 113 4918 4921 19258598 10.1182/blood-2008-08-174110 PMC2686142

[bib18] Compagno M Lim WK Grunn A Nandula SV Brahmachary M Shen Q Mutations of multiple genes cause deregulation of NF-kappaB in diffuse large B-cell lymphoma Nature 2009 459 717 721 19412164 10.1038/nature07968 PMC2973325

[bib19] Ngo VN Young RM Schmitz R Jhavar S Xiao W Lim KH Oncogenically active MYD88 mutations in human lymphoma Nature 2011 470 115 119 21179087 10.1038/nature09671 PMC5024568

[bib20] Morin RD Mendez-Lago M Mungall AJ Goya R Mungall KL Corbett RD Frequent mutation of histone-modifying genes in non-Hodgkin lymphoma Nature 2011 476 298 303 21796119 10.1038/nature10351 PMC3210554

[bib21] Pasqualucci L Trifonov V Fabbri G Ma J Rossi D Chiarenza A Analysis of the coding genome of diffuse large B-cell lymphoma Nat Genet 2011 43 830 837 21804550 10.1038/ng.892 PMC3297422

[bib22] Lohr JG Stojanov P Lawrence MS Auclair D Chapuy B Sougnez C Discovery and prioritization of somatic mutations in diffuse large B-cell lymphoma (DLBCL) by whole-exome sequencing Proc Natl Acad Sci USA 2012 109 3879 3884 22343534 10.1073/pnas.1121343109 PMC3309757

[bib23] Barton S Hawkes EA Wotherspoon A Cunningham D Are we ready to stratify treatment for diffuse large B-cell lymphoma using molecular hallmarks Oncologist 2012 17 1562 1573 23086691 10.1634/theoncologist.2012-0218 PMC3528389

[bib24] Klapper W Stoecklein H Zeynalova S Ott G Kosari F Rosenwald A Structural aberrations affecting the MYC locus indicate a poor prognosis independent of clinical risk factors in diffuse large B-cell lymphomas treated within randomized trials of the German High-Grade Non-Hodgkin's Lymphoma Study Group (DSHNHL) Leukemia 2008 22 2226 2229 18754028 10.1038/leu.2008.230

[bib25] Drake MT Maurer MJ Link BK Habermann TM Ansell SM Micallef IN Vitamin D insufficiency and prognosis in non-Hodgkin's lymphoma J Clin Oncol 2010 28 4191 4198 20713849 10.1200/JCO.2010.28.6674 PMC2953973

[bib26] Wright G Tan B Rosenwald A Hurt EH Wiestner A Staudt LM A gene expression-based method to diagnose clinically distinct subgroups of diffuse large B cell lymphoma Proc Natl Acad Sci USA 2003 100 9991 9996 12900505 10.1073/pnas.1732008100 PMC187912

[bib27] Hans CP Weisenburger DD Greiner TC Gascoyne RD Delabie J Ott G Confirmation of the molecular classification of diffuse large B-cell lymphoma by immunohistochemistry using a tissue microarray Blood 2004 103 275 282 14504078 10.1182/blood-2003-05-1545

[bib28] Livak KJ Schmittgen TD Analysis of relative gene expression data using real-time quantitative PCR and the 2(-Delta Delta C(T)) Method Methods 2001 25 402 408 11846609 10.1006/meth.2001.1262

[bib29] Hastings PJ Lupski JR Rosenberg SM Ira G Mechanisms of change in gene copy number Nat Rev Genet 2009 10 551 564 19597530 10.1038/nrg2593 PMC2864001

[bib30] Tirado CA Chen W Garcia R Kohlman KA Rao N Genomic profiling using array comparative genomic hybridization define distinct subtypes of diffuse large B-cell lymphoma: a review of the literature J Hematol Oncol 2012 5 54 22967872 10.1186/1756-8722-5-54 PMC3479011

[bib31] Trinh DL Scott DW Morin RD Mendez-Lago M An J Jones SJ Analysis of FOXO1 mutations in diffuse large B-cell lymphoma Blood 2013 121 3666 3674 23460611 10.1182/blood-2013-01-479865 PMC3643765

[bib32] Steidl C Shah SP Woolcock BW Rui L Kawahara M Farinha P MHC class II transactivator CIITA is a recurrent gene fusion partner in lymphoid cancers Nature 2011 471 377 381 21368758 10.1038/nature09754 PMC3902849

[bib33] Okabe M Unno M Harigae H Kaku M Okitsu Y Sasaki T Characterization of the organic cation transporter SLC22A16: a doxorubicin importer Biochem Biophys Res Commun 2005 333 754 762 15963465 10.1016/j.bbrc.2005.05.174

[bib34] Aouida M Poulin R Ramotar D The human carnitine transporter SLC22A16 mediates high affinity uptake of the anticancer polyamine analogue bleomycin-A5 J Biol Chem 2010 285 6275 6284 20037140 10.1074/jbc.M109.046151 PMC2825423

[bib35] Rosenwald A Wright G Chan WC Connors JM Campo E Fisher RI The use of molecular profiling to predict survival after chemotherapy for diffuse large-B-cell lymphoma N Engl J Med 2002 346 1937 1947 12075054 10.1056/NEJMoa012914

[bib36] Huang JZ Sanger WG Greiner TC Staudt LM Weisenburger DD Pickering DL The t(14;18) defines a unique subset of diffuse large B-cell lymphoma with a germinal center B-cell gene expression profile Blood 2002 99 2285 2290 11895757 10.1182/blood.v99.7.2285

[bib37] Bea S Zettl A Wright G Salaverria I Jehn P Moreno V Diffuse large B-cell lymphoma subgroups have distinct genetic profiles that influence tumor biology and improve gene-expression-based survival prediction Blood 2005 106 3183 3190 16046532 10.1182/blood-2005-04-1399 PMC1895326

[bib38] Chen W Houldsworth J Olshen AB Nanjangud G Chaganti S Venkatraman ES Array comparative genomic hybridization reveals genomic copy number changes associated with outcome in diffuse large B-cell lymphomas Blood 2006 107 2477 2485 16317097 10.1182/blood-2005-07-2950 PMC1895737

[bib39] Bea S Colomo L Lopez-Guillermo A Salaverria I Puig X Pinyol M Clinicopathologic significance and prognostic value of chromosomal imbalances in diffuse large B-cell lymphomas J Clin Oncol 2004 22 3498 3506 15337798 10.1200/JCO.2004.11.025

[bib40] Monti S Chapuy B Takeyama K Rodig SJ Hao Y Yeda KT Integrative analysis reveals an outcome-associated and targetable pattern of p53 and cell cycle deregulation in diffuse large B cell lymphoma Cancer Cell 2012 22 359 372 22975378 10.1016/j.ccr.2012.07.014 PMC3778921

[bib41] Karnan S Tagawa H Suzuki R Suguro M Yamaguchi M Okamoto M Analysis of chromosomal imbalances in de novo CD5-positive diffuse large-B-cell lymphoma detected by comparative genomic hybridization Genes Chromosomes Cancer 2004 39 77 81 14603444 10.1002/gcc.10298

[bib42] Schmidt MH Dikic I The Cbl interactome and its functions Nat Rev Mol Cell Biol 2005 6 907 918 16227975 10.1038/nrm1762

[bib43] Novak AJ Grote DM Stenson M Ziesmer SC Witzig TE Habermann TM Expression of BLyS and its receptors in B-cell non-Hodgkin lymphoma: correlation with disease activity and patient outcome Blood 2004 104 2247 2253 15251985 10.1182/blood-2004-02-0762

[bib44] Hildebrand JM Luo Z Manske MK Price-Troska T Ziesmer SC Lin W A BAFF-R mutation associated with non-Hodgkin lymphoma alters TRAF recruitment and reveals new insights into BAFF-R signaling J Exp Med 2010 207 2569 2579 21041452 10.1084/jem.20100857 PMC2989778

[bib45] Sagaert X Van Cutsem E De Hertogh G Geboes K Tousseyn T Gastric MALT lymphoma: a model of chronic inflammation-induced tumor development Nat Rev Gastroenterol Hepatol 2010 7 336 346 20440281 10.1038/nrgastro.2010.58

[bib46] Lim KH Yang Y Staudt LM Pathogenetic importance and therapeutic implications of NF-kappaB in lymphoid malignancies Immunol Rev 2012 246 359 378 22435566 10.1111/j.1600-065X.2012.01105.x PMC4094296

[bib47] Muzio G Maggiora M Paiuzzi E Oraldi M Canuto RA Aldehyde dehydrogenases and cell proliferation Free Radical Biol Med 2012 52 735 746 22206977 10.1016/j.freeradbiomed.2011.11.033

[bib48] Sladek NE Aldehyde dehydrogenase-mediated cellular relative insensitivity to the oxazaphosphorines Curr Pharm Des 1999 5 607 625 10469894

[bib49] Davis RE Ngo VN Lenz G Tolar P Young RM Romesser PB Chronic active B-cell-receptor signalling in diffuse large B-cell lymphoma Nature 2010 463 88 92 20054396 10.1038/nature08638 PMC2845535

[bib50] Chaturvedi A Martz R Dorward D Waisberg M Pierce SK Endocytosed BCRs sequentially regulate MAPK and Akt signaling pathways from intracellular compartments Nat Immunol 2011 12 1119 1126 21964606 10.1038/ni.2116 PMC3746798

[bib51] Xu-Monette ZY Wu L Visco C Tai YC Tzankov A Liu WM Mutational profile and prognostic significance of TP53 in diffuse large B-cell lymphoma patients treated with R-CHOP: report from an International DLBCL Rituximab-CHOP Consortium Program Study Blood 2012 120 3986 3996 22955915 10.1182/blood-2012-05-433334 PMC3496956

[bib52] Young KH Leroy K Moller MB Colleoni GW Sanchez-Beato M Kerbauy FR Structural profiles of TP53 gene mutations predict clinical outcome in diffuse large B-cell lymphoma: an international collaborative study Blood 2008 112 3088 3098 18559976 10.1182/blood-2008-01-129783 PMC2569165

[bib53] Fernandez-Rodriguez C Bellosillo B Garcia-Garcia M Sanchez-Gonzalez B Gimeno E Vela MC MYD88 (L265P) mutation is an independent prognostic factor for outcome in patients with diffuse large B-cell lymphoma Leukemia 2014 28 2104 2106 24903481 10.1038/leu.2014.184

