# Supplementary material for: Whole-exome analysis reveals novel somatic genomic alterations associated with outcome in immunochemotherapy-treated diffuse large B-cell lymphoma
Source: Blood Cancer J. 2015 Aug 28;5(8):e346–. doi: 10.1038/bcj.2015.69 (PMC4558593; doi:10.1038/bcj.2015.69)
Supplement: Supplementary Information [file bcj201569x1.doc]

**Supplemental Methods**

**Whole-exome Sequencing**

WES of DNA from 51 DLBCL tumors-normal pairs was performed at the Broad Institute as previously described.[^1^](#_ENREF_1) The WES reads were mapped to Human Reference Genome Build 37 using Novoalign (http://www.novocraft.com/main/index.php), and SNVs and small insertions and deletions (INDELs) were called using GATK.[^2^](#_ENREF_2) Somatic mutations were identified using SomaticSniper.[^3^](#_ENREF_3) The variant calling algorithm uses a Gaussian mixture model combining multiple parameters to give one single score which determines if the variant is called or not. 43/51 paired samples were also genotyped using the Affymetrix 6.0 SNP array. For SNP microarray data, CNAs were called using an R package crlmm[^4^](#_ENREF_4) and further refined using a hidden Markov model based algorithm named VanillaICE.[^5^](#_ENREF_5)

**Copy Number Analysis**

The exon-level copy number alterations (CNAs) were identified using patternCNV,[^6^](#_ENREF_6) which calls CNAs based on the observation that the read coverage in exome-sequencing varies between exons in a given sample but is consistent across samples. Since we used a relatively stringent threshold to identify exon-level CNAs, the gene-level CNAs were assigned if one exon of a gene was identified with CNAs. If multiple exons within a gene gave conflicting CNV calls, the gene was excluded from further analysis. We included the following somatic mutations in the analysis: non-synonymous SNVs, SNVs resulting in stop and start codon gain or loss, SNVs located in the splice sites, and frame-shifting INDELs. The variant annotations were generated using SNPEFF (<http://snpeff.sourceforge.net/SnpEff_paper.pdf>). All SNVs and INDELs with minor allele frequencies (MAF) greater than 5% among the Caucasian populations in the HapMap (http://hapmap.ncbi.nlm.nih.gov/) or 1000 Genomes Project (http://www.1000genomes.org/) were filtered out. In Figures 3-4, a CNA was called for each chromosomal region based on the loss or gain of the following genes; 3q27·3 (*BCL6)*, 11q23·3 (*CBL*), 19q13·32 (*RELB*), and 6q21 (*SLC22A16*).

CNA loss of SLC22A16 was also measured by quantitative PCR. DNA from a validation cohort of DLBCL tumors (n=43) was extracted and analyzed for *SLC22A16* copy number by real-time PCR (RT-PCR). The *SLC22A16* copy number changes were assessed utilizing a commercial TaqMan® Copy Number Assay from Applied Biosystems (cat # Hs01339225_cn). Amplification and analysis was performed on the CFX96 Real Time System (BioRad). The analysis focused on the relative quantification located within exon 4 of the gene. A reference target, TaqMan Copy Number Reference Assay, RNase P (cat#4403326), was included in the reaction for standardization of copy number targets. The reaction was performed in a 25 μL volume, including 9.5 uL PCR grade water, 12.5 uL 2X HotStar Master Mix Buffer (Qiagen), 1.0 μL primer/probe mix for each amplicon (SLC22A16A-FAM labeled and REF-VIC labeled), and 1 uL (250 ng) template DNA. Amplification conditions were: 15 min at 95 °C, followed by 40 cycles of 95 °C for 15 s, 60°C for 60 s. The FAM/VIC signal was recorded after each anneal/extension cycle. Each sample was run in triplicate. Quantification was done using standard ΔΔC_t_ analysis using the CFX96 software. In the assay set up, the average ΔΔC_t_ of exon 4 of *SLC22A16* in non-malignant lymphoid tissue (negative control) was 1.04 ± 0.05 (range 0.98-1.09, n=11) and for DLBCL tissue that had a CNA loss called by WES (positive control, range 0.53-0.67, n=4) was 0.57 ± 0.13. For our analysis we called a sample positive for an SLC22A16 loss if it had a value of 0.83 or less (0.57 plus 2 standard deviations).

**Bioinformatic Modeling of SLC22A16 Mutations**

For the structural modeling of SLC22A16 mutations, a homology model was generated for SLC22A16 using the sequence from UniProt[^10^](#_ENREF_10) entry NP_149116.2 and the I-Tasser server.[^11^](#_ENREF_11) This model was refined using the method of Haddadian *et al*.[^12^](#_ENREF_12) annotated with domains and functional features according to Pfam,[^13^](#_ENREF_13) UniProt, and CDD,[^14^](#_ENREF_14) and visualized using PyMOL.

**References**

1. Lohr JG, Stojanov P, Lawrence MS, et al. Discovery and prioritization of somatic mutations in diffuse large B-cell lymphoma (DLBCL) by whole-exome sequencing. Proc Natl Acad Sci U S A. 2012;109(10):3879-3884.

2. McKenna A, Hanna M, Banks E, et al. The Genome Analysis Toolkit: a MapReduce framework for analyzing next-generation DNA sequencing data. Genome Res. 2010;20(9):1297-1303.

3. Larson DE, Harris CC, Chen K, et al. SomaticSniper: identification of somatic point mutations in whole genome sequencing data. Bioinformatics. 2012;28(3):311-317.

4. Scharpf RB, Irizarry RA, Ritchie ME, Carvalho B, Ruczinski I. Using the R Package crlmm for Genotyping and Copy Number Estimation. J Stat Softw. 2011;40(12):1-32.

5. Scharpf RB, Parmigiani G, Pevsner J, Ruczinski I. Hidden Markov models for the assessment of chromosomal alterations using high-throughput SNP arrays. Ann Appl Stat. 2008;2(2):687-713.

6. Wang C, Evans JM, Bhagwate AV, et al. PatternCNV: a versatile tool for detecting copy number changes from exome sequencing data. Bioinformatics. 2014.

7. Wright G, Tan B, Rosenwald A, Hurt EH, Wiestner A, Staudt LM. A gene expression-based method to diagnose clinically distinct subgroups of diffuse large B cell lymphoma. Proceedings of the National Academy of Sciences. 2003;100(17):9991-9996.

8. Jais JP, Haioun C, Molina TJ, et al. The expression of 16 genes related to the cell of origin and immune response predicts survival in elderly patients with diffuse large B-cell lymphoma treated with CHOP and rituximab. Leukemia. 2008;22(10):1917-1924.

9. Hans CP, Weisenburger DD, Greiner TC, et al. Confirmation of the molecular classification of diffuse large B-cell lymphoma by immunohistochemistry using a tissue microarray. Blood. 2004;103(1):275-282.

10. Magrane M, Consortium U. UniProt Knowledgebase: a hub of integrated protein data. Database : the journal of biological databases and curation. 2011;2011:bar009.

11. Roy A, Kucukural A, Zhang Y. I-TASSER: a unified platform for automated protein structure and function prediction. Nature protocols. 2010;5(4):725-738.

12. Haddadian EJ, Gong H, Jha AK, et al. Automated real-space refinement of protein structures using a realistic backbone move set. Biophysical journal. 2011;101(4):899-909.

13. Finn RD, Bateman A, Clements J, et al. Pfam: the protein families database. Nucleic acids research. 2014;42(Database issue):D222-230.

14. Marchler-Bauer A, Zheng C, Chitsaz F, et al. CDD: conserved domains and protein three-dimensional structure. Nucleic acids research. 2013;41(Database issue):D348-352.
