# Supplementary figures and images for: Whole-exome analysis reveals novel somatic genomic alterations associated with outcome in immunochemotherapy-treated diffuse large B-cell lymphoma
Source: Blood Cancer J. 2015 Aug 28;5(8):e346–. doi: 10.1038/bcj.2015.69 (PMC4558593; doi:10.1038/bcj.2015.69)

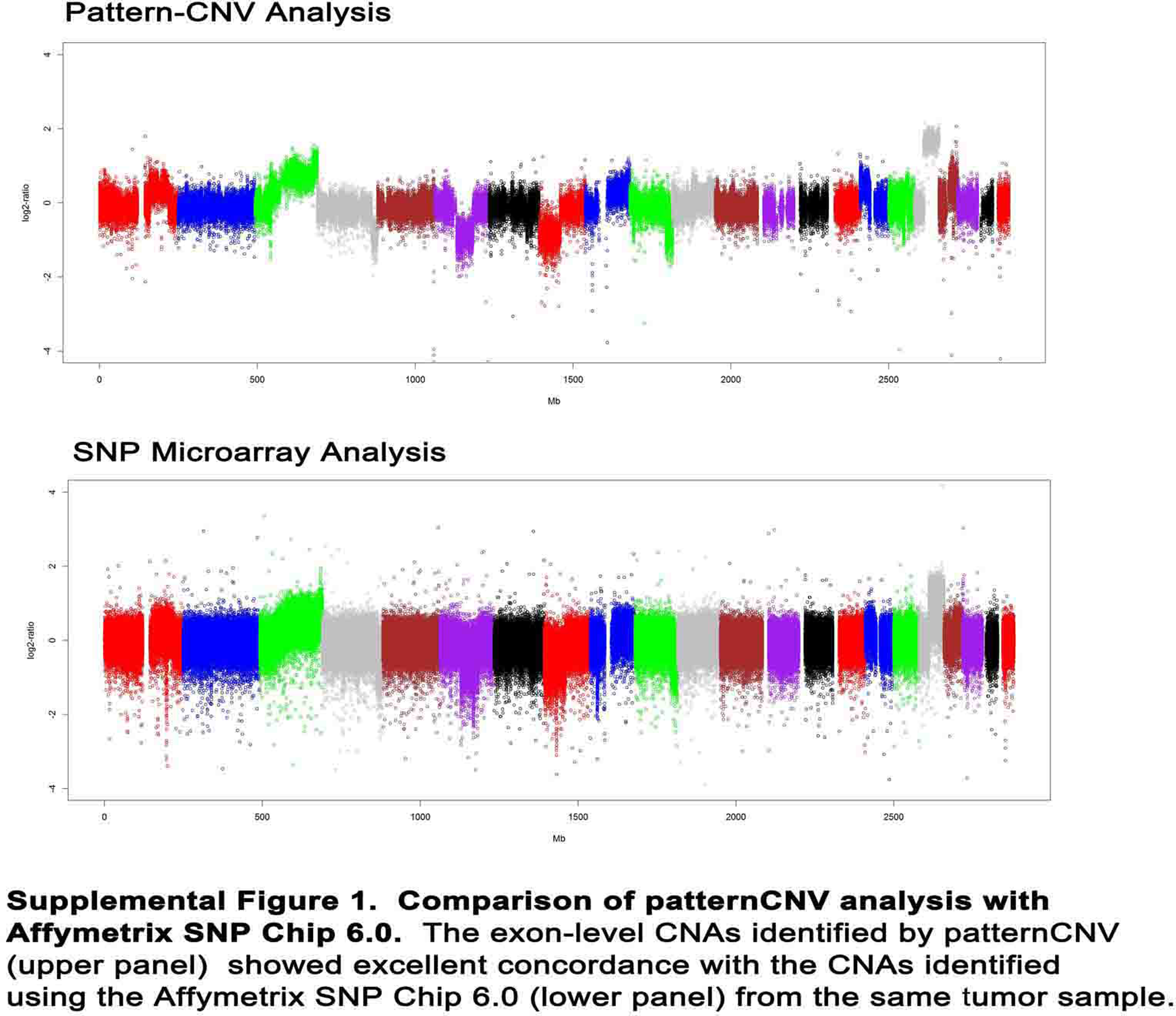

Supplement: Supplementary Figure 1 [file bcj201569x2.tif]

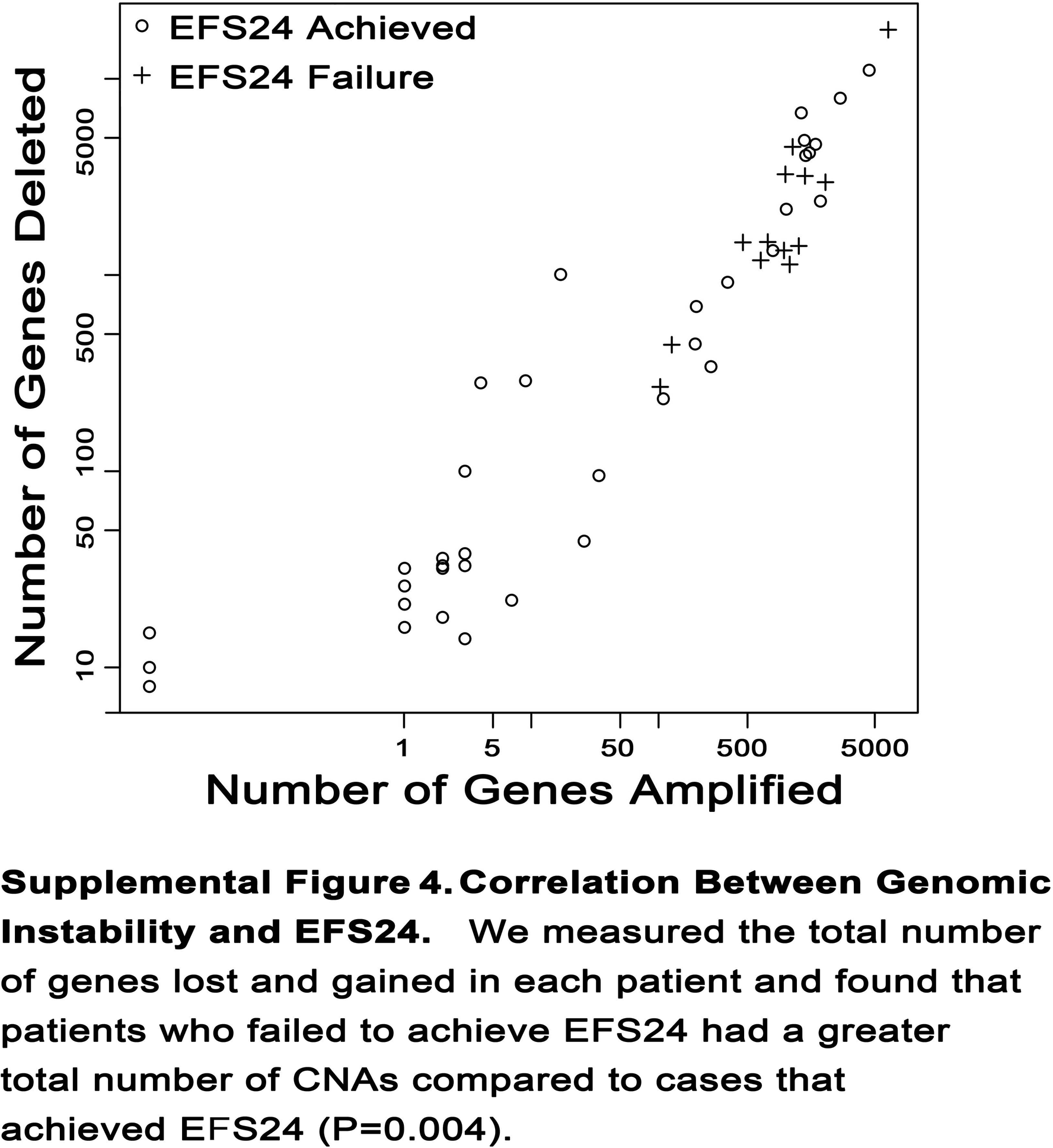

Supplement: Supplementary Figure 4 [file bcj201569x5.tif]

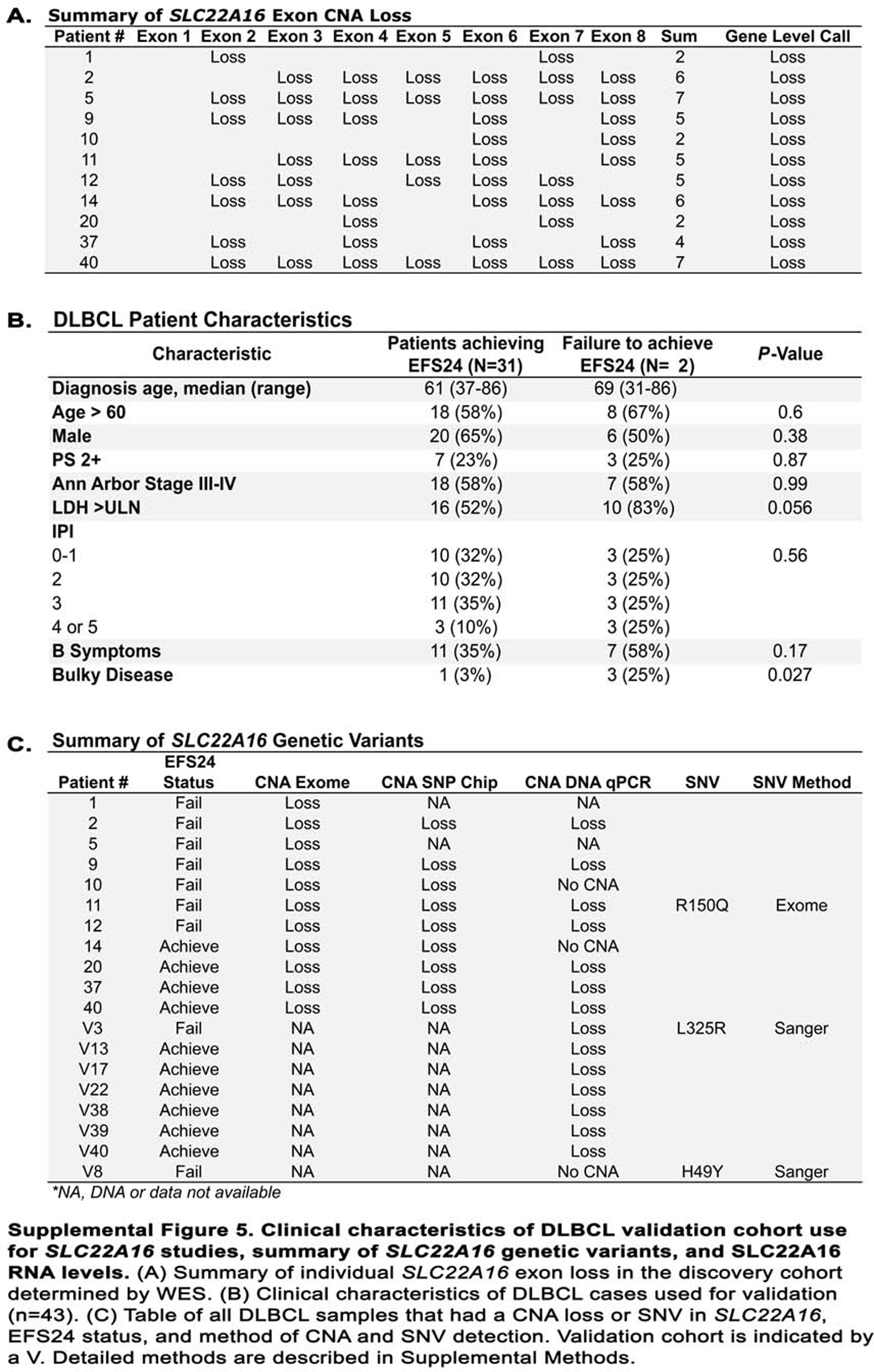

Supplement: Supplementary Figure 5 [file bcj201569x6.tif]

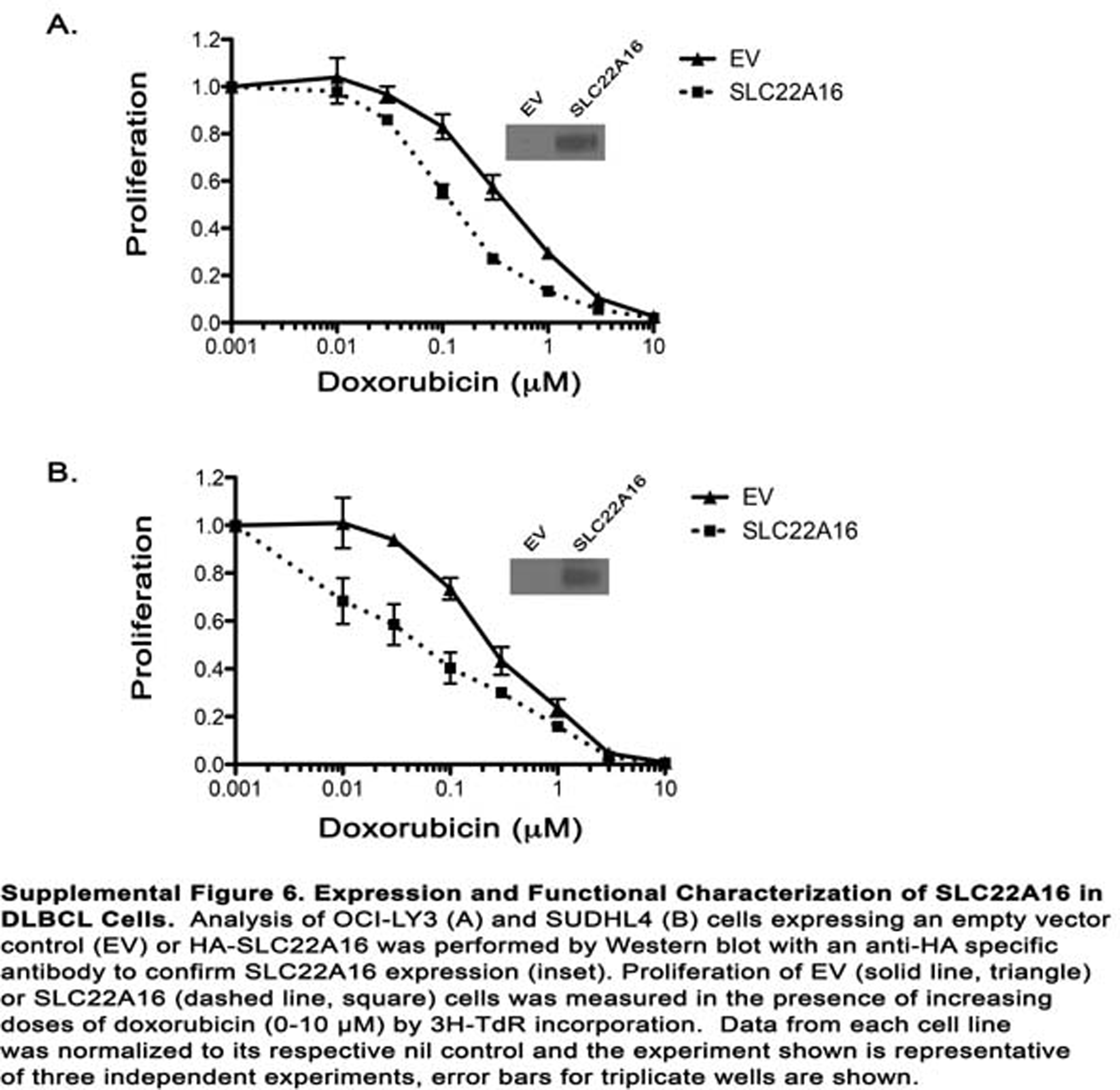

Supplement: Supplementary Figure 6 [file bcj201569x7.tif]

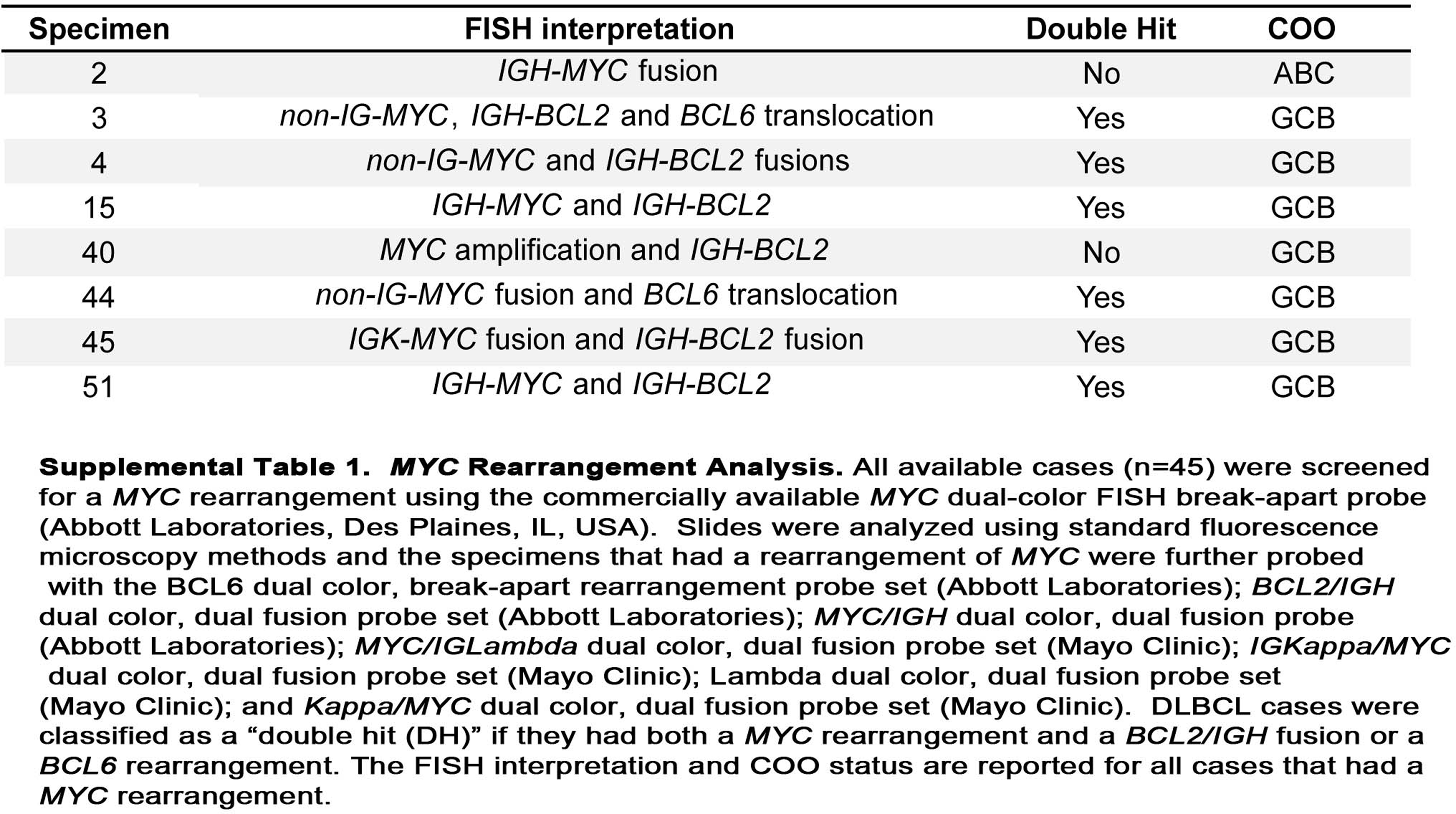

Supplement: Supplementary Table 1 [file bcj201569x8.tif]
