# Supplementary material for: Whole-exome analysis reveals novel somatic genomic alterations associated with outcome in immunochemotherapy-treated diffuse large B-cell lymphoma
Source: Blood Cancer J. 2015 Aug 28;5(8):e346–. doi: 10.1038/bcj.2015.69 (PMC4558593; doi:10.1038/bcj.2015.69)
Supplement: Supplementary Figure 2 [file bcj201569x3.pdf]

Supplemental Figure 2. Individual patternCNV analysis of 51 DLBCL tumors. The log ratio values between the sequencing coverage of individual tumors and the "average pattern" was calculated by patternCNV using all paired normal samples. Concatenated chromosomal positions are shown on the X-axis and individual chromosomes (Chr 1-22) are displayed in order by color.

Patient Sample 1

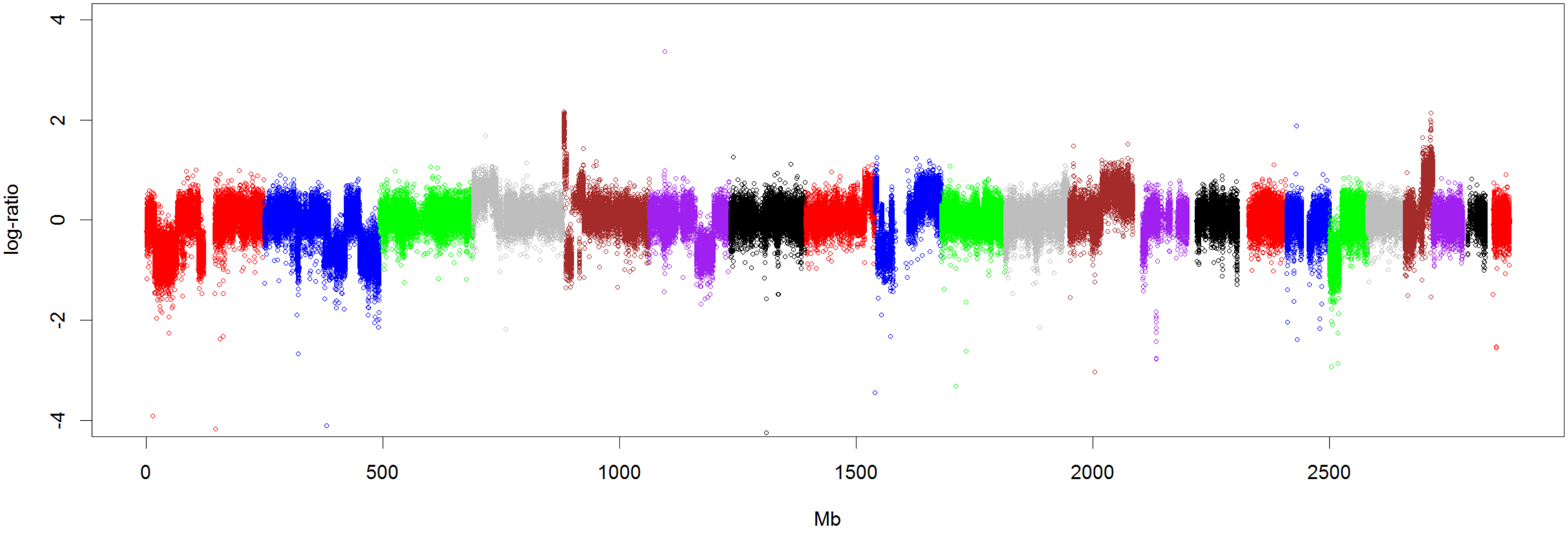

Patient Sample 2

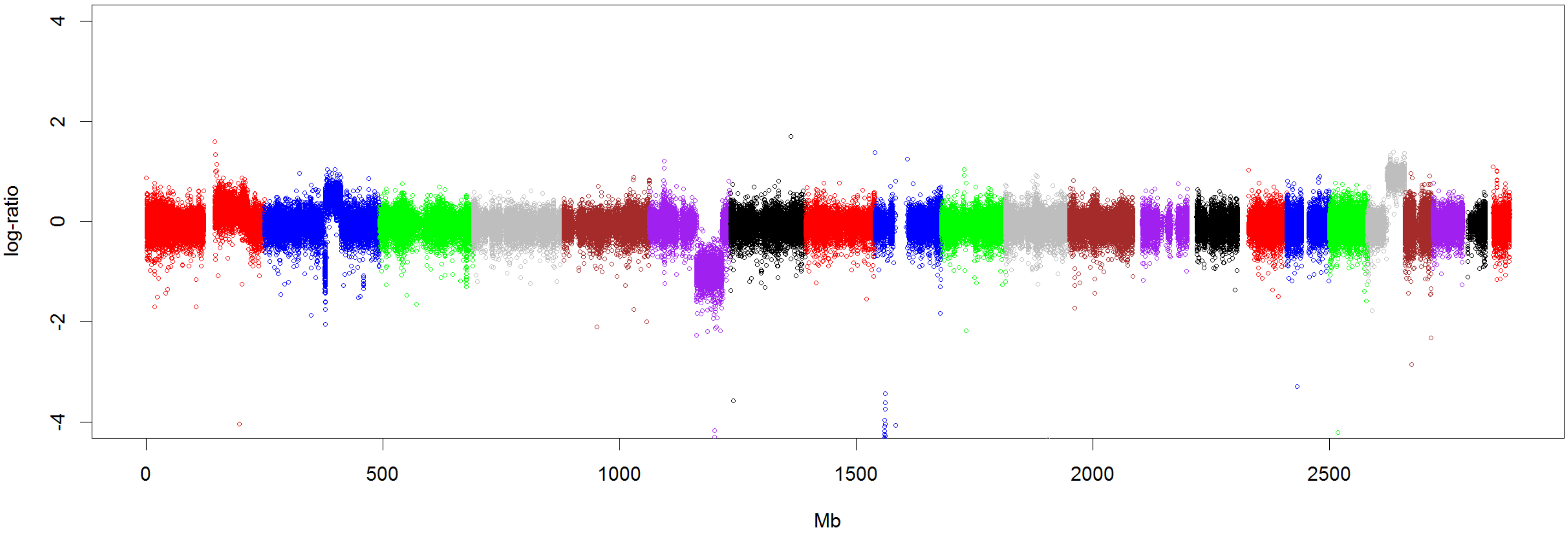

Patient Sample 3

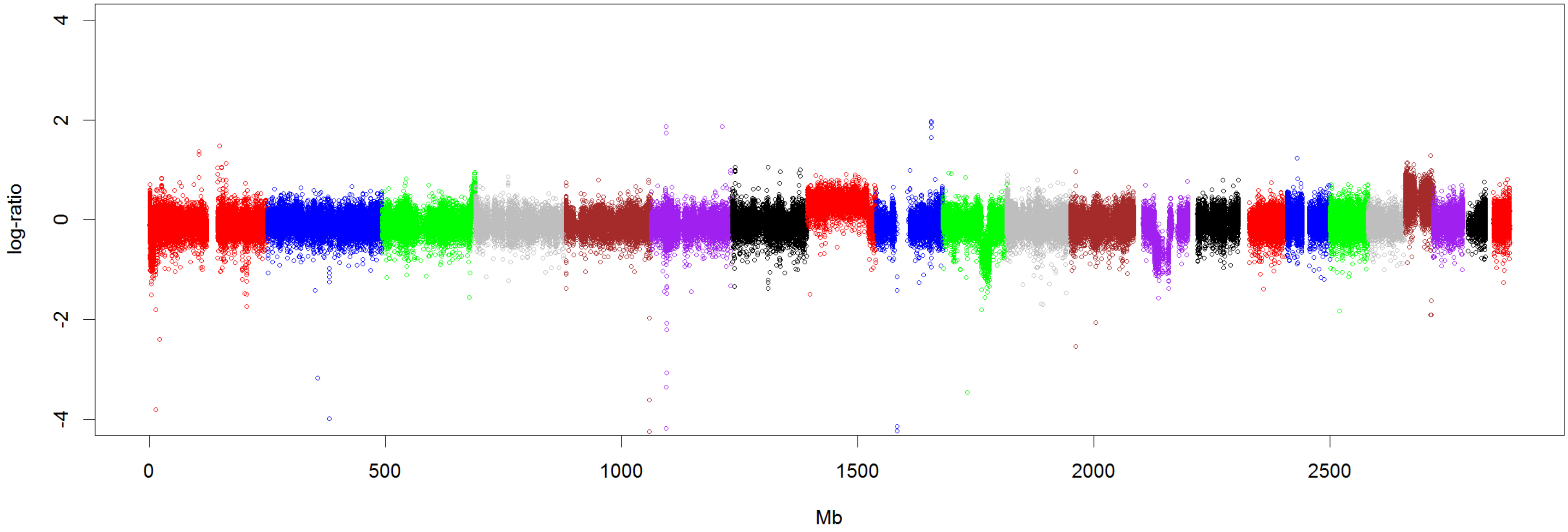

Patient Sample 4

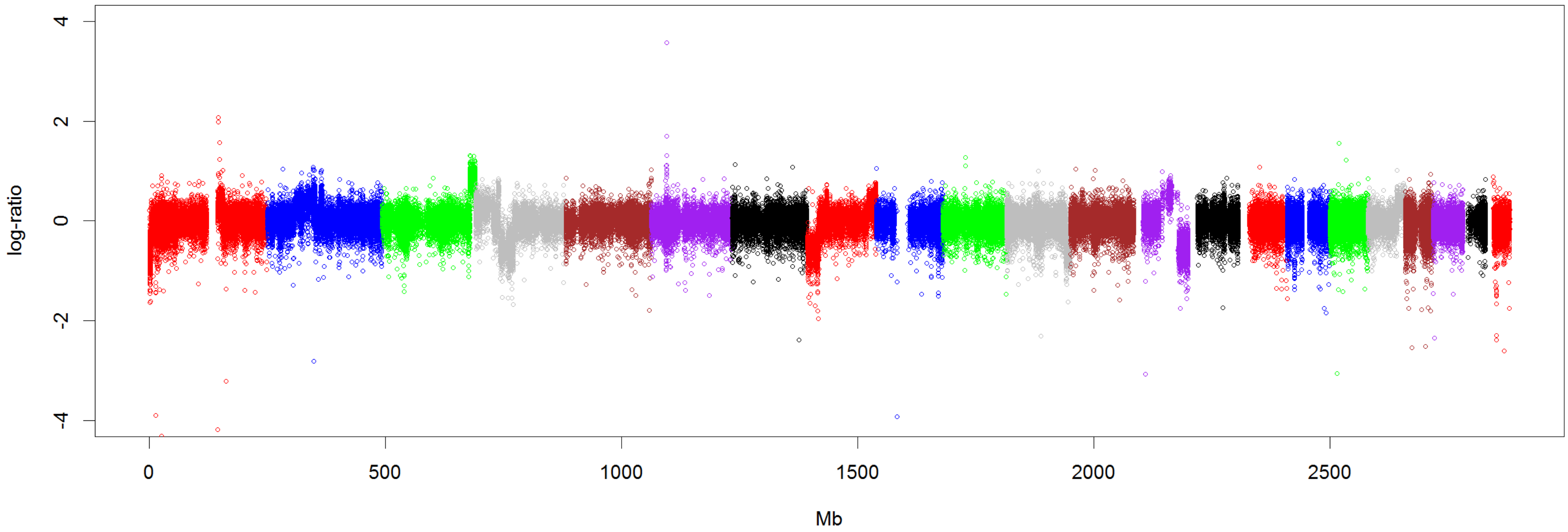

Patient Sample 5

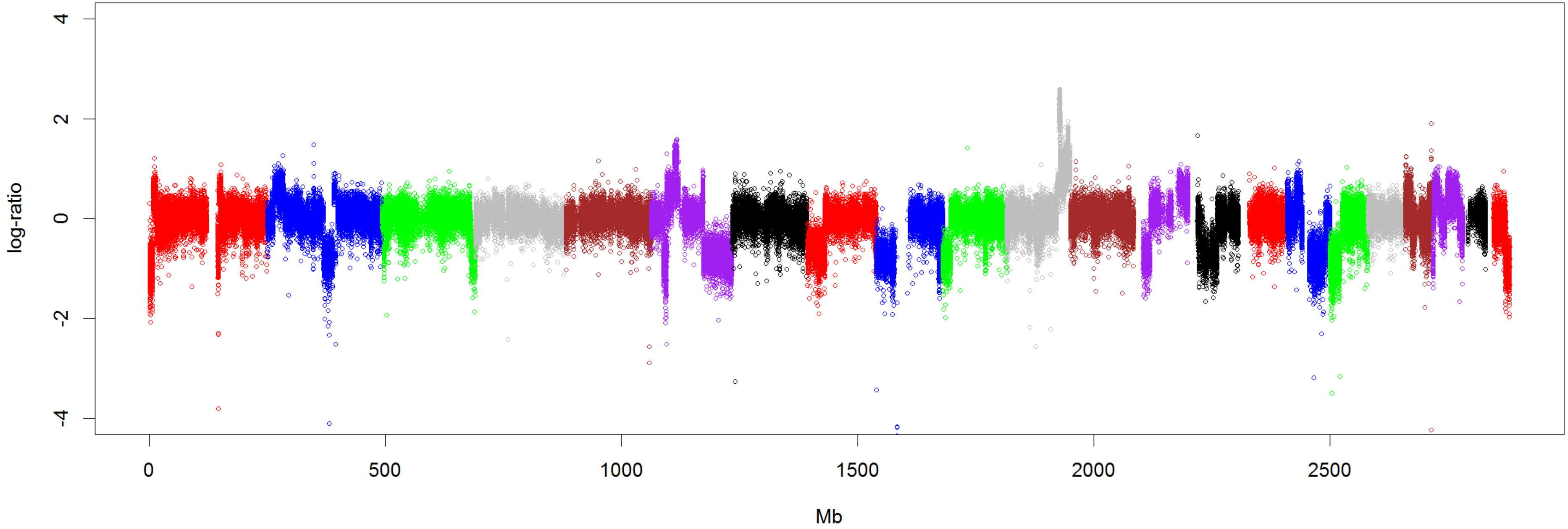

Patient Sample 6

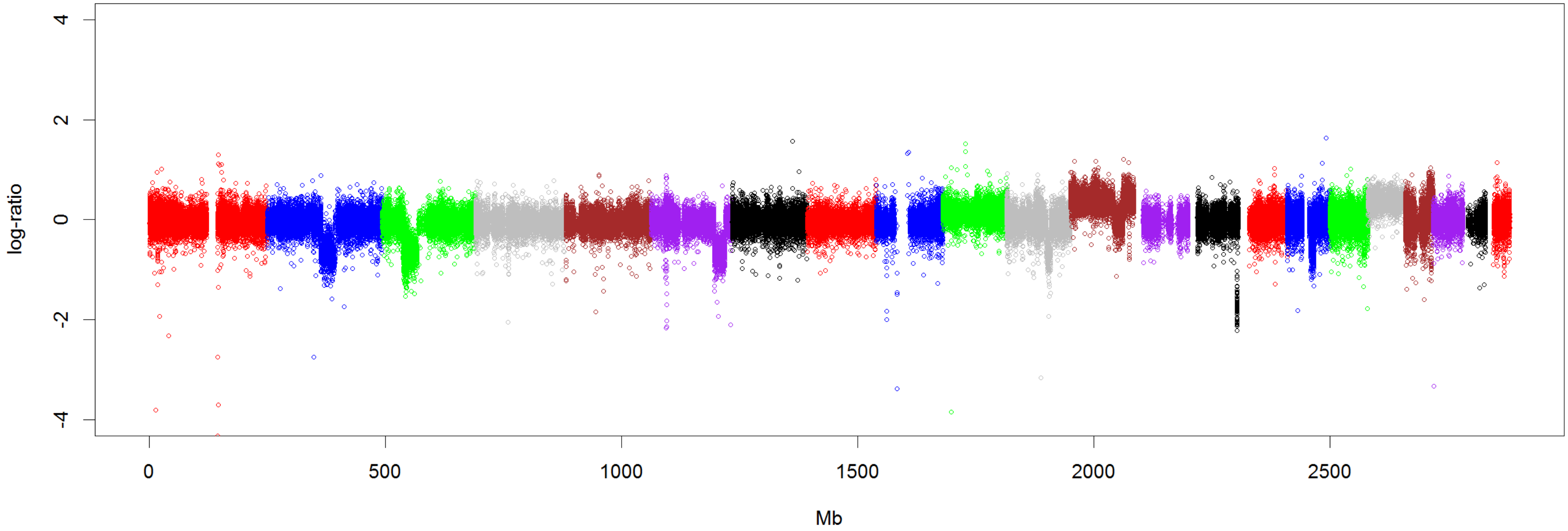

Patient Sample 7

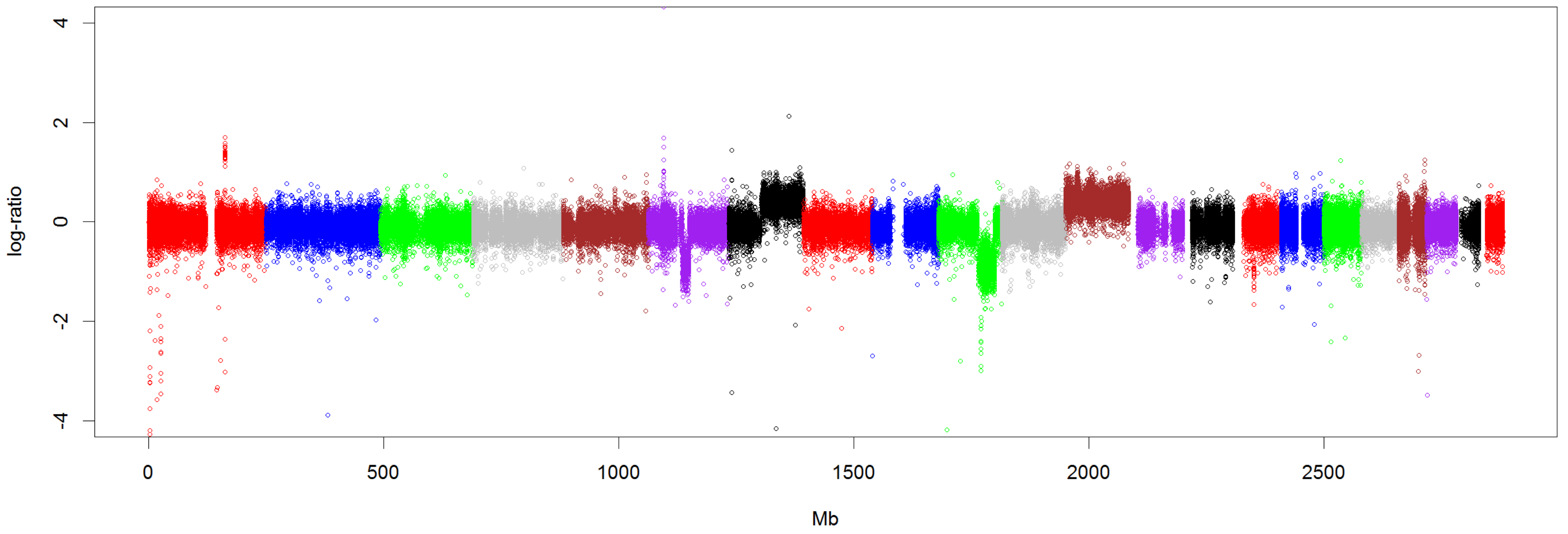

Patient Sample 8

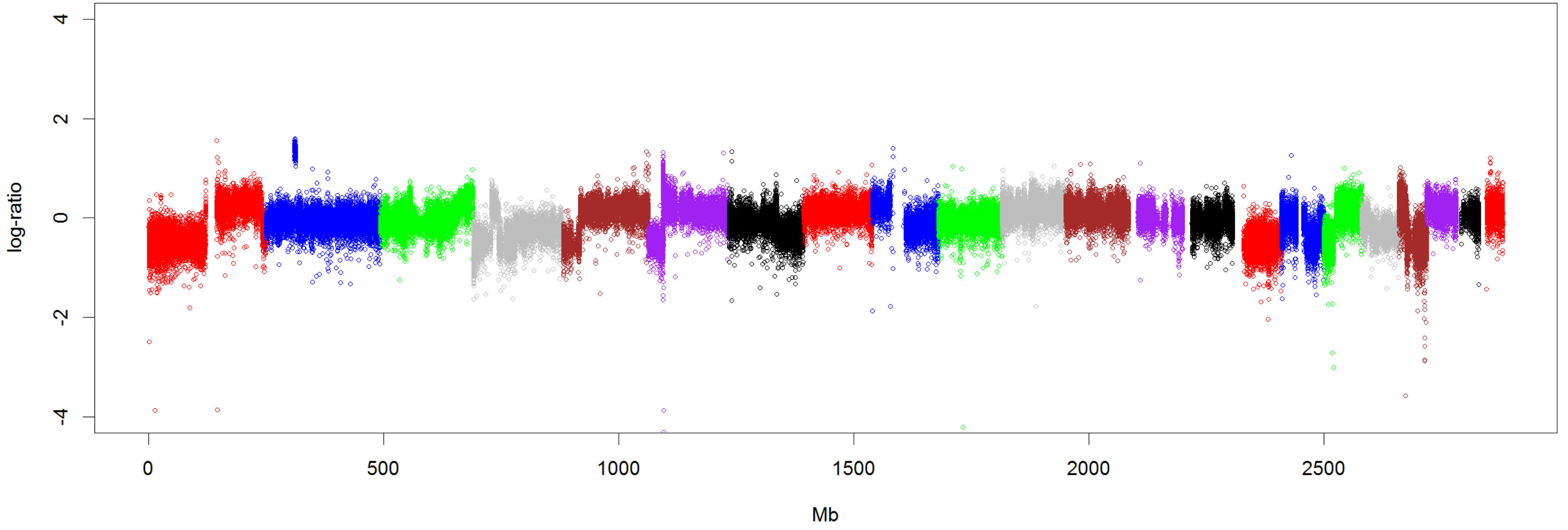

Patient Sample 9

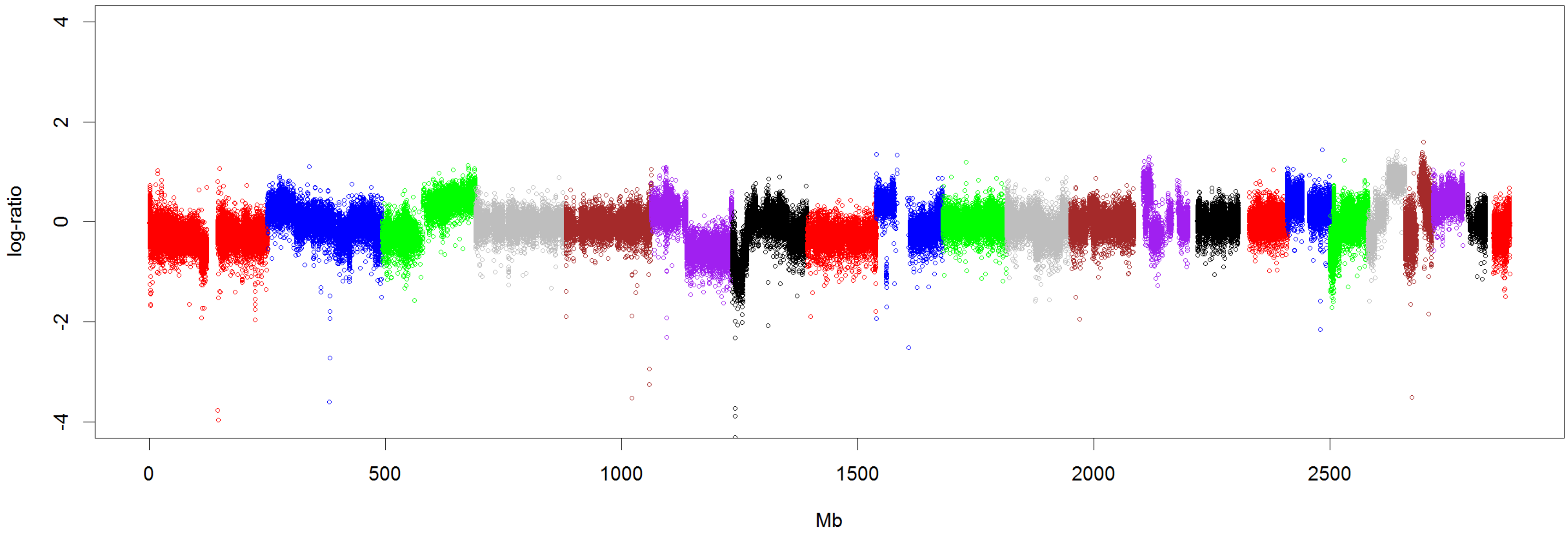

Patient Sample 10

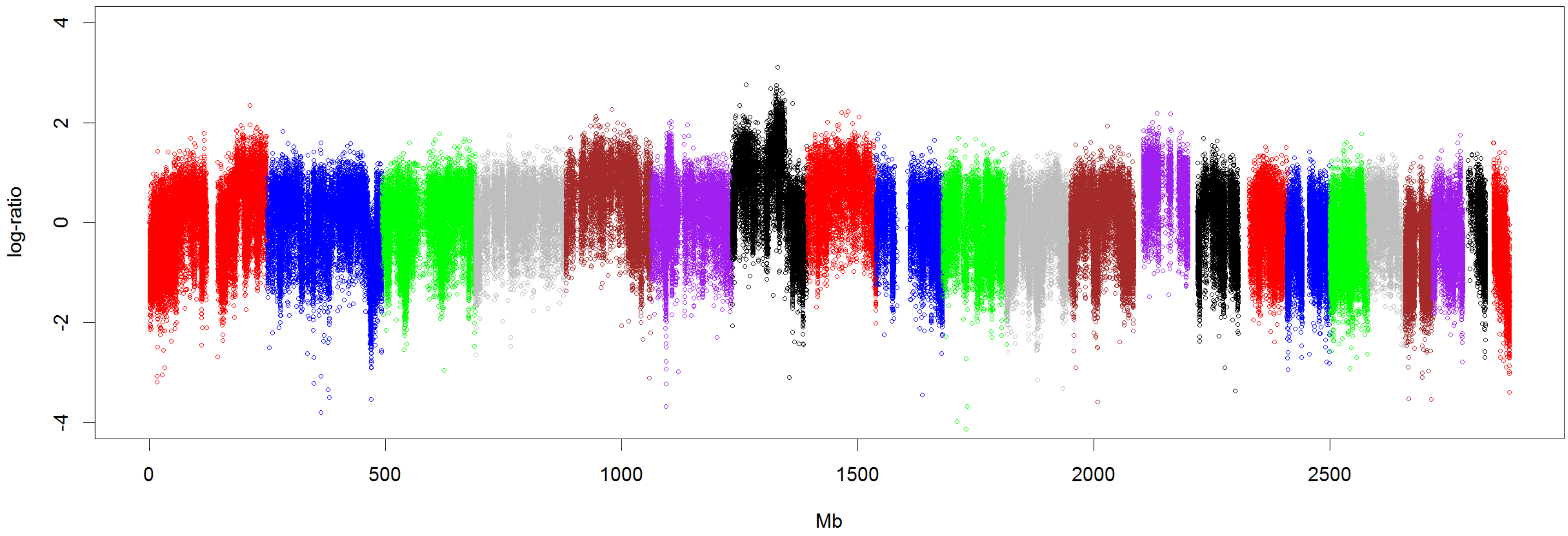

Patient Sample 11

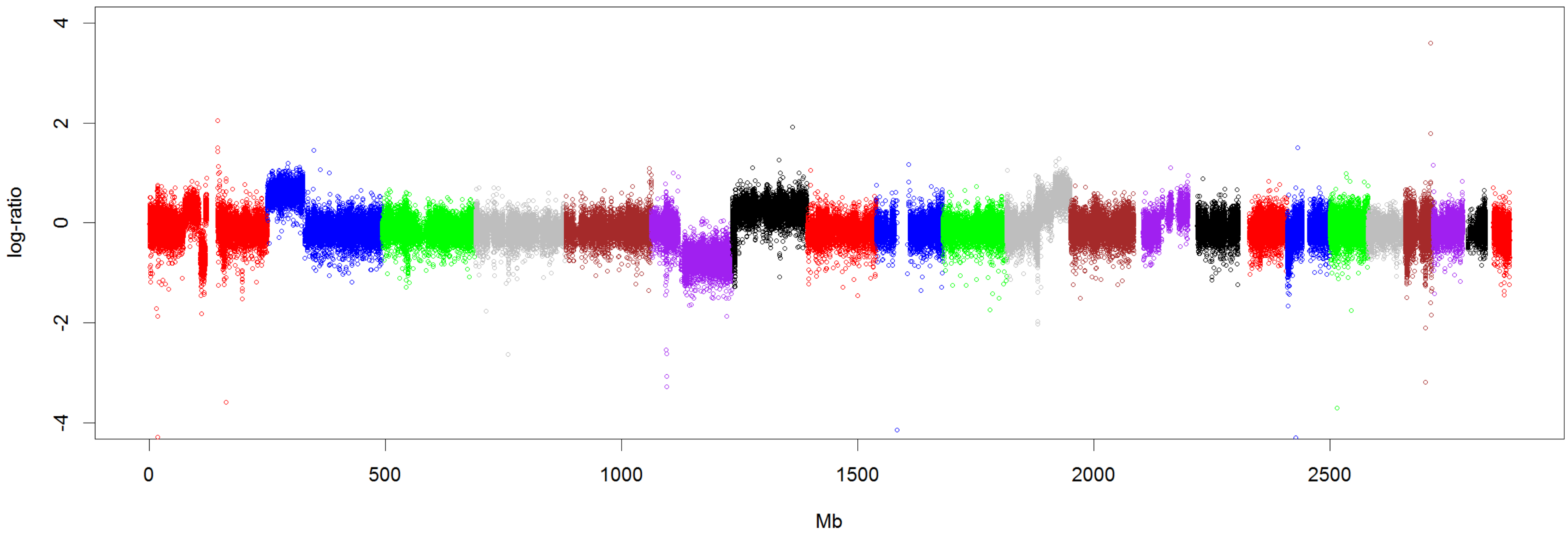

Patient Sample 12

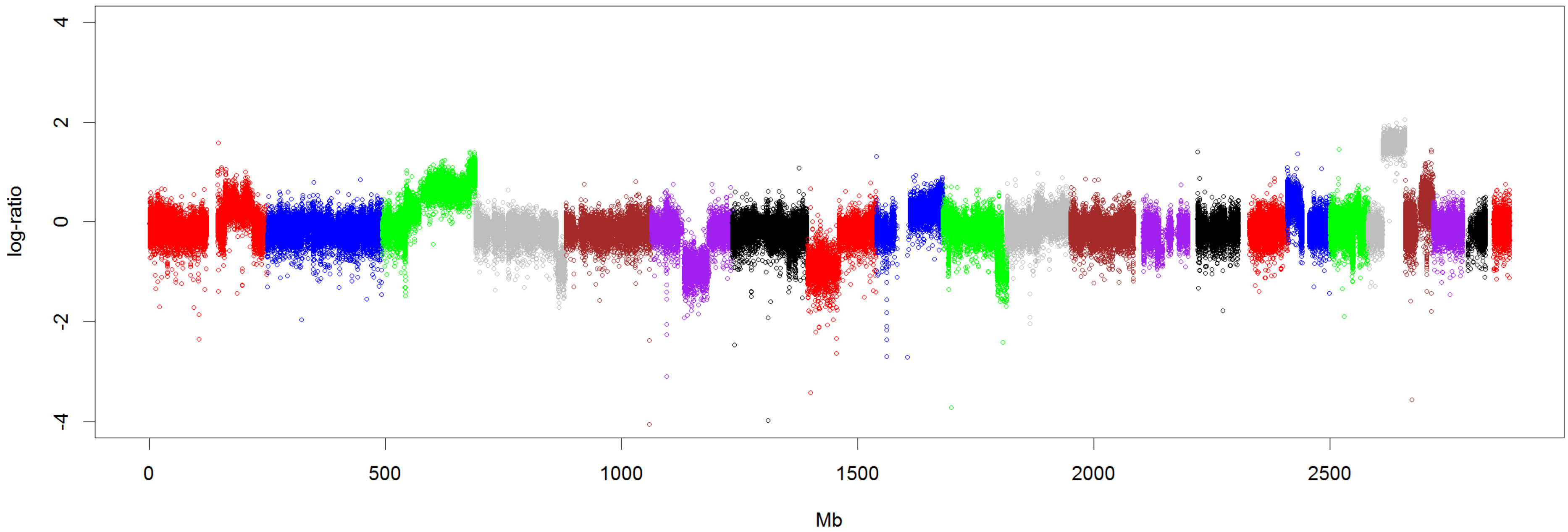

Patient Sample 13

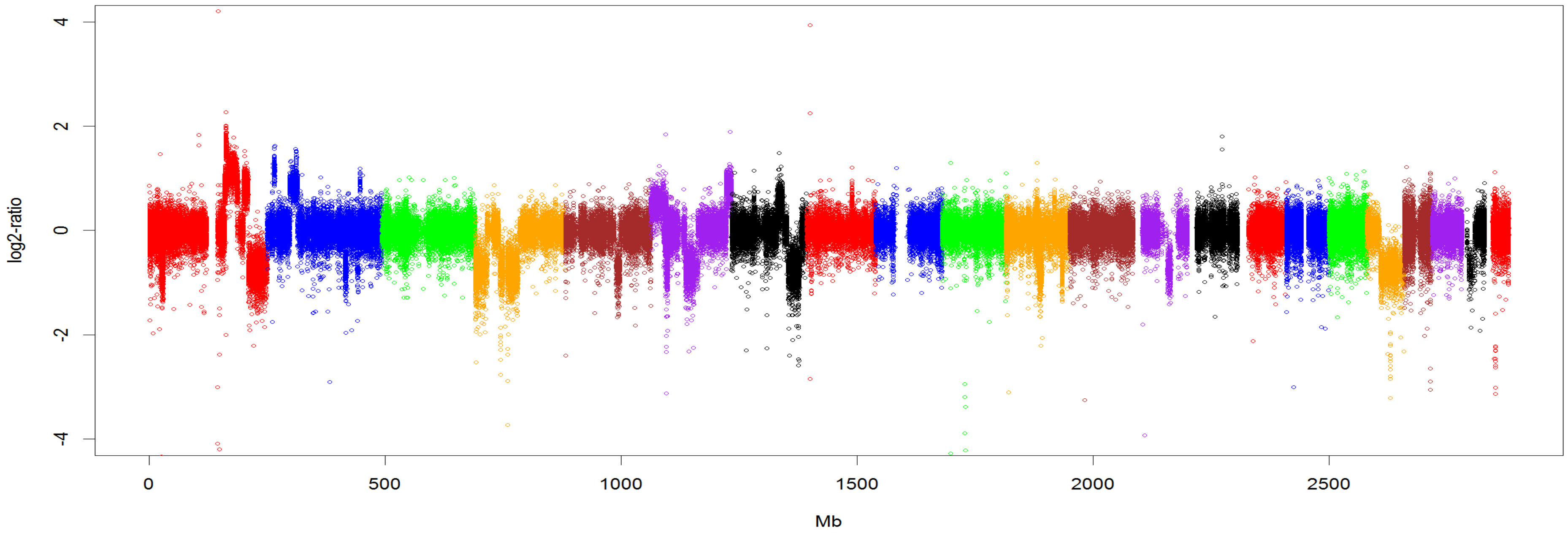

Patient Sample 14

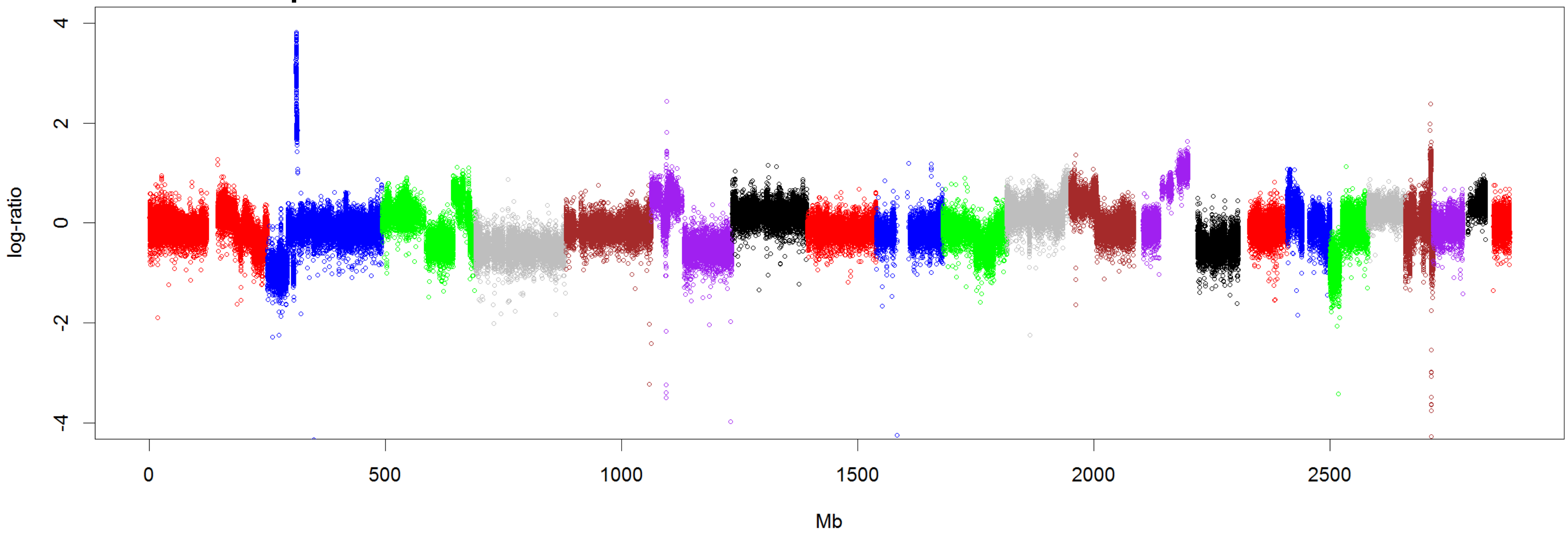

Patient Sample 15

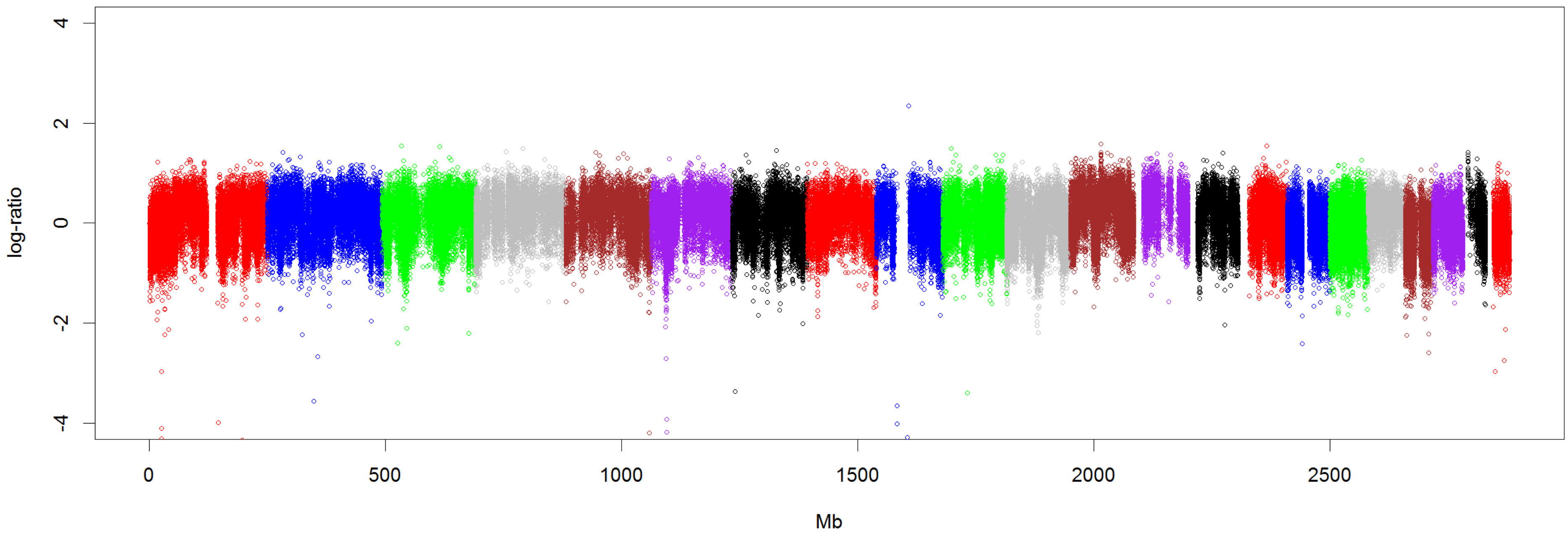

Patient Sample 16

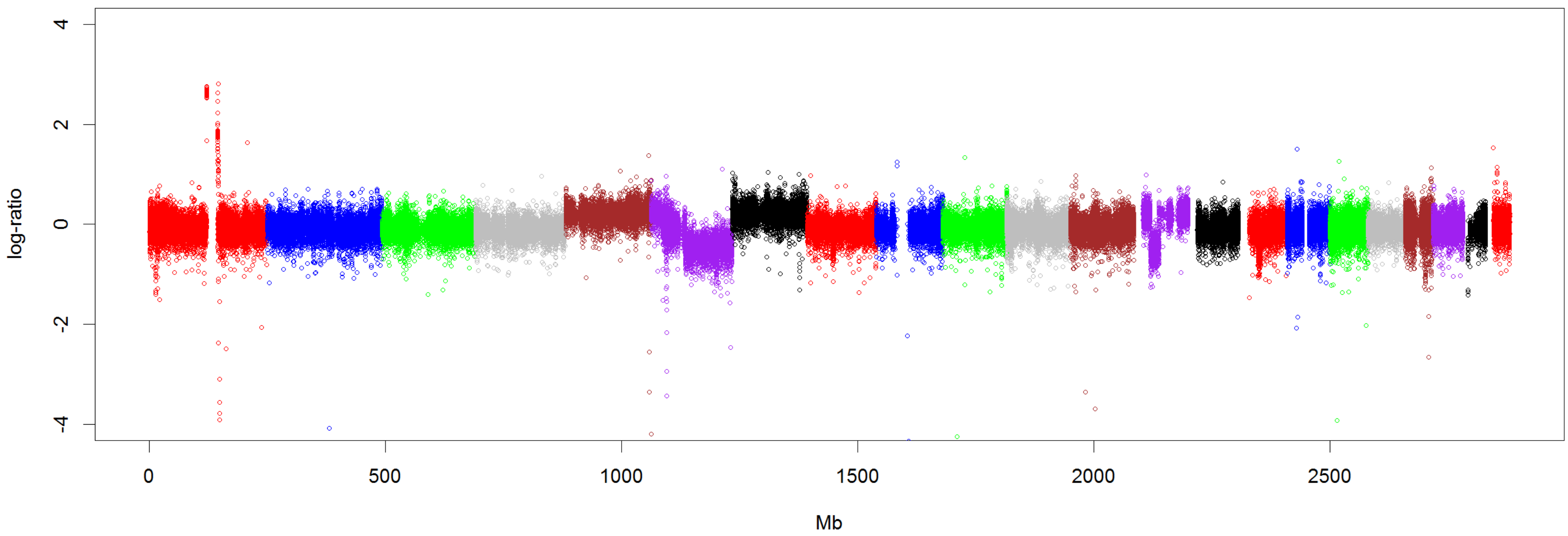

Patient Sample 17

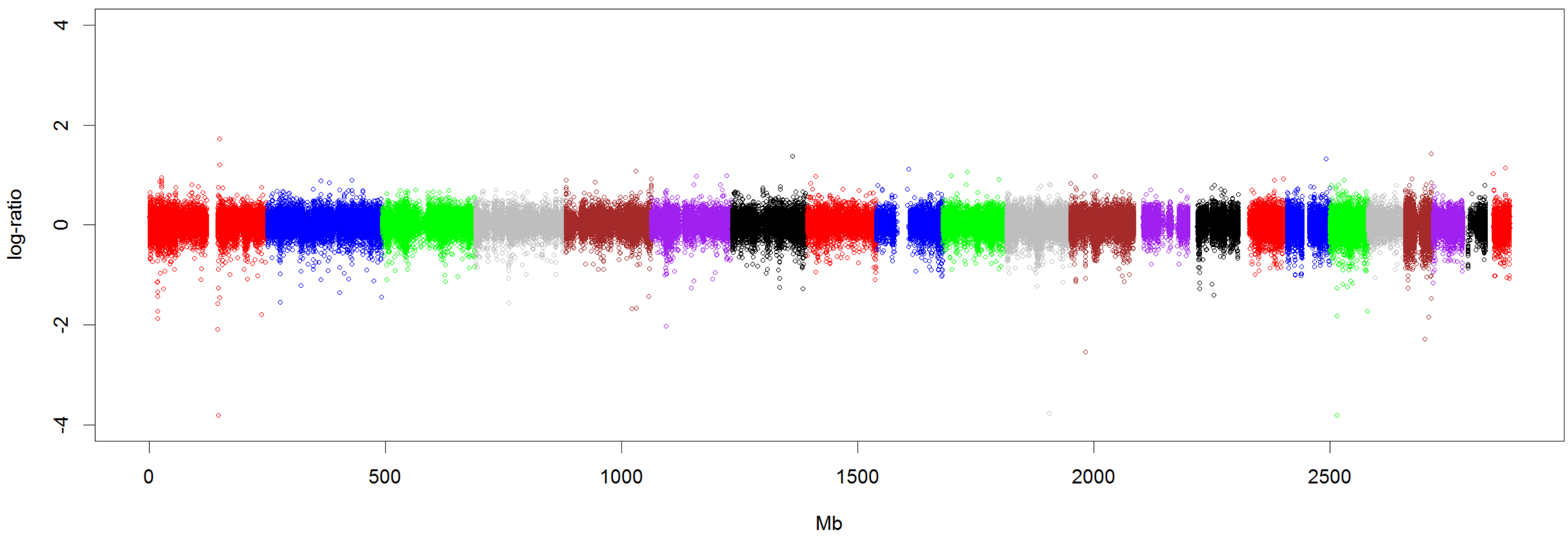

Patient Sample 18

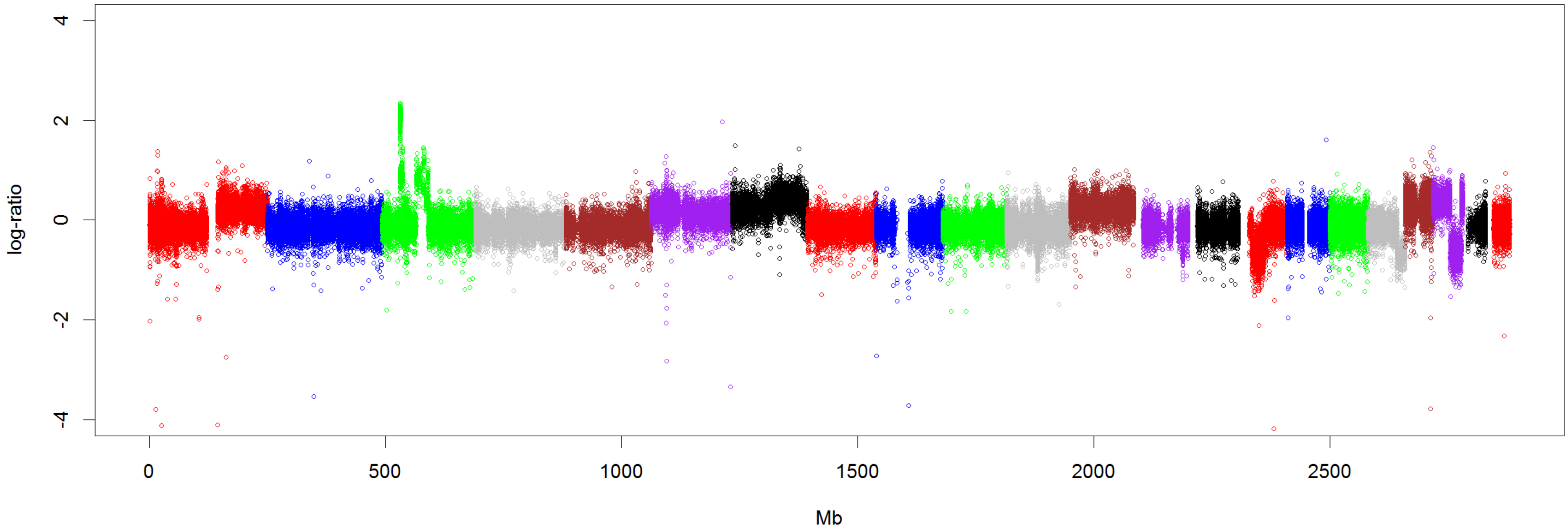

Patient Sample 19

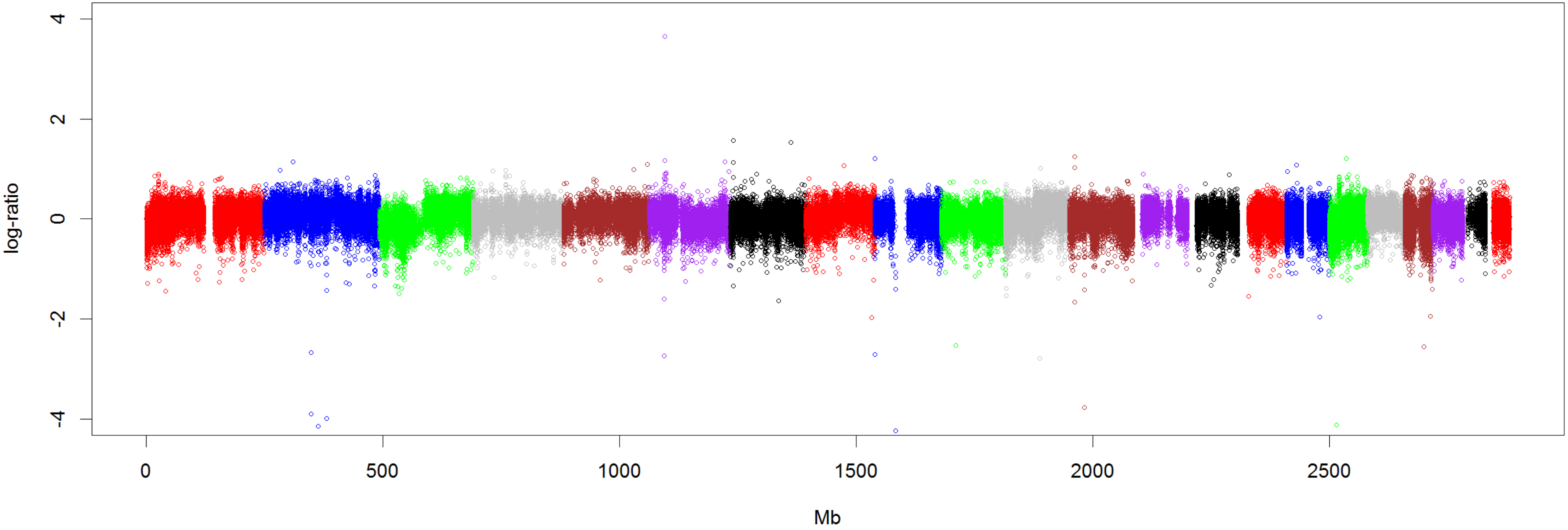

Patient Sample 20

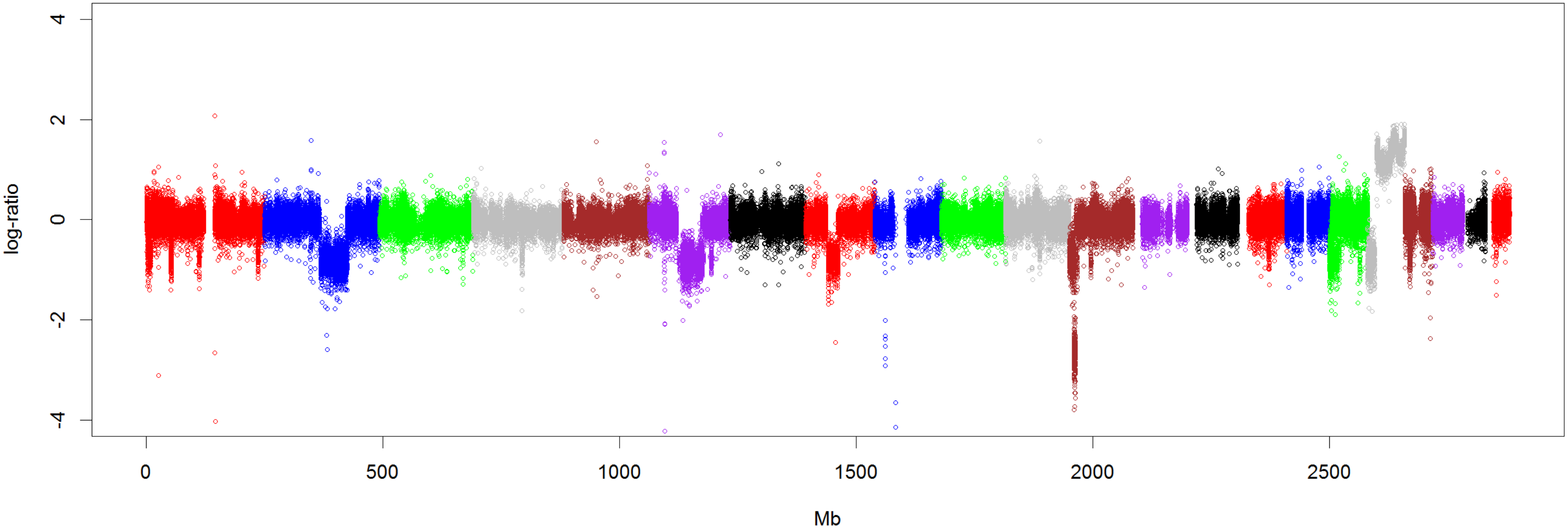

Patient Sample 21

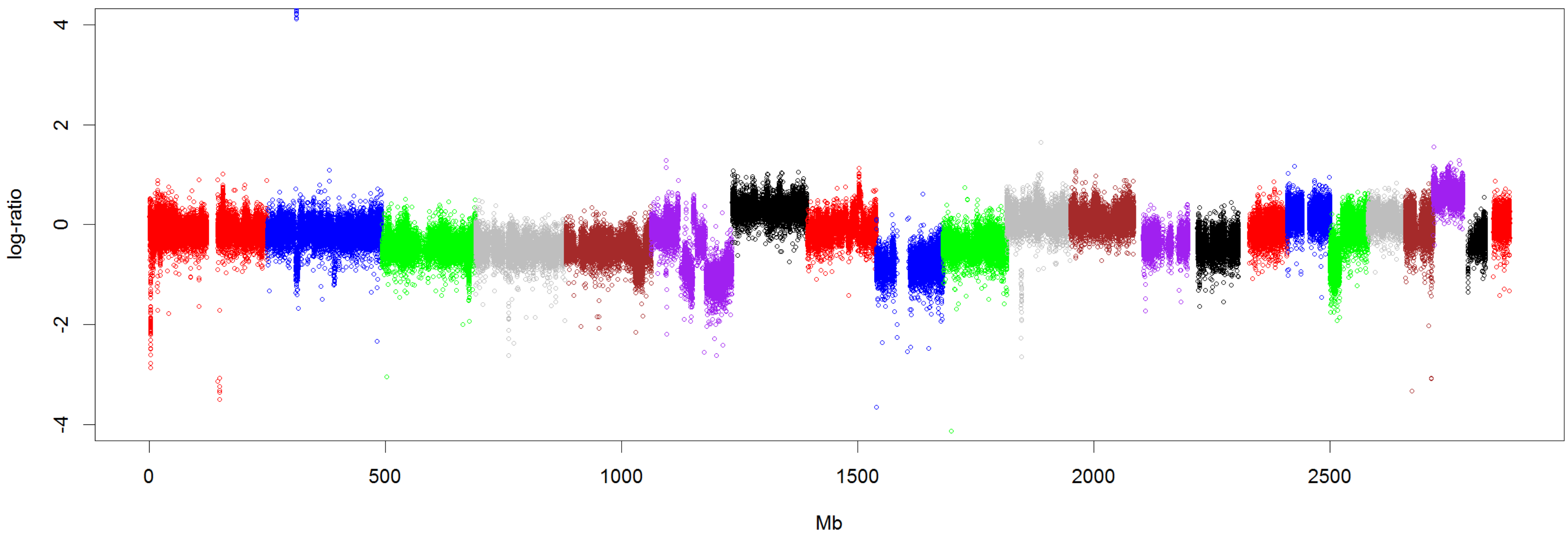

Patient Sample 22

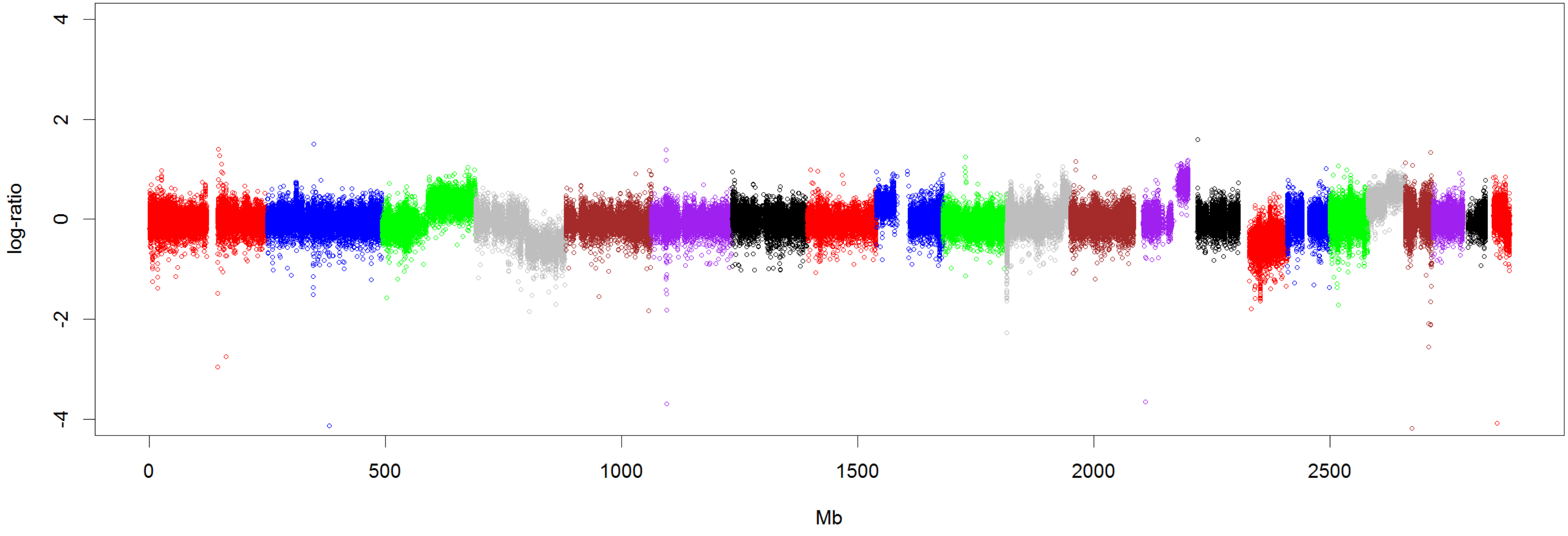

Patient Sample 23

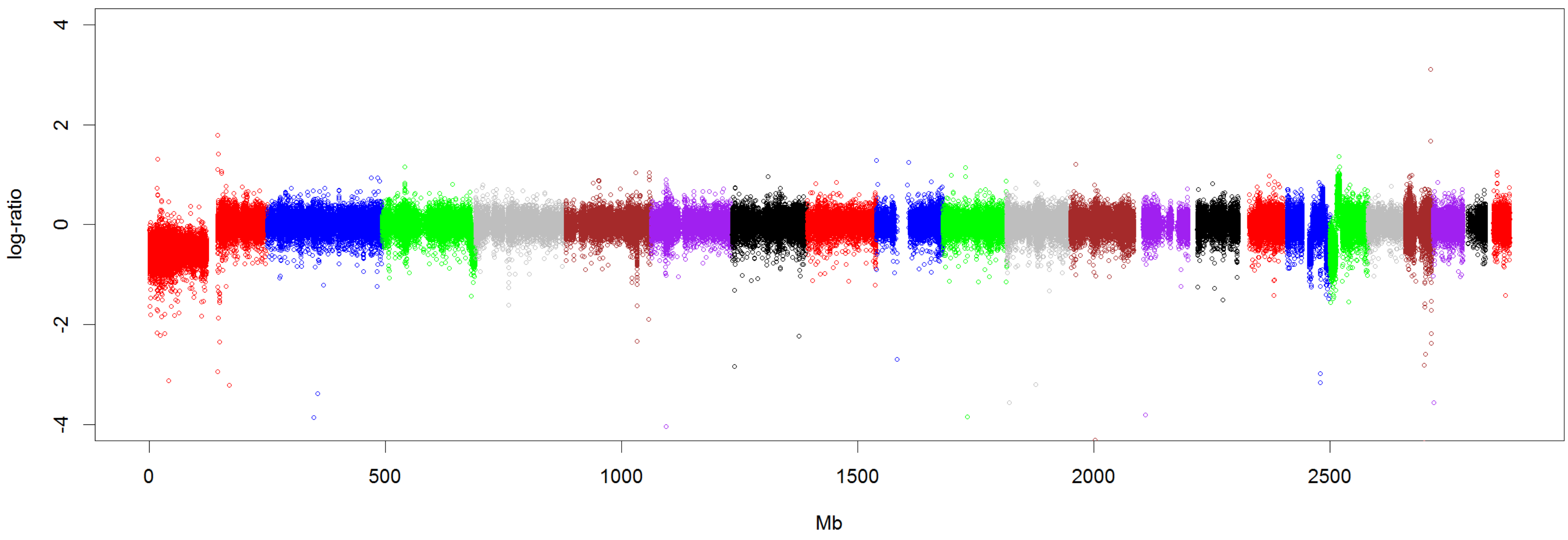

Patient Sample 24

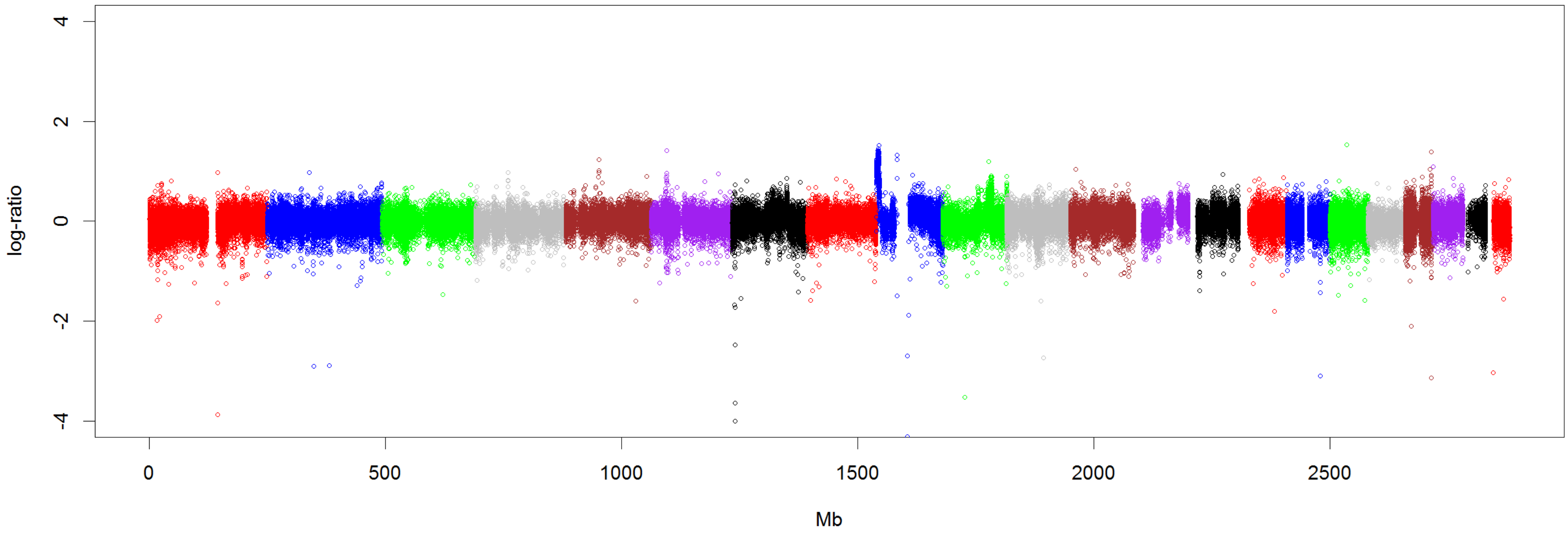

Patient Sample 25

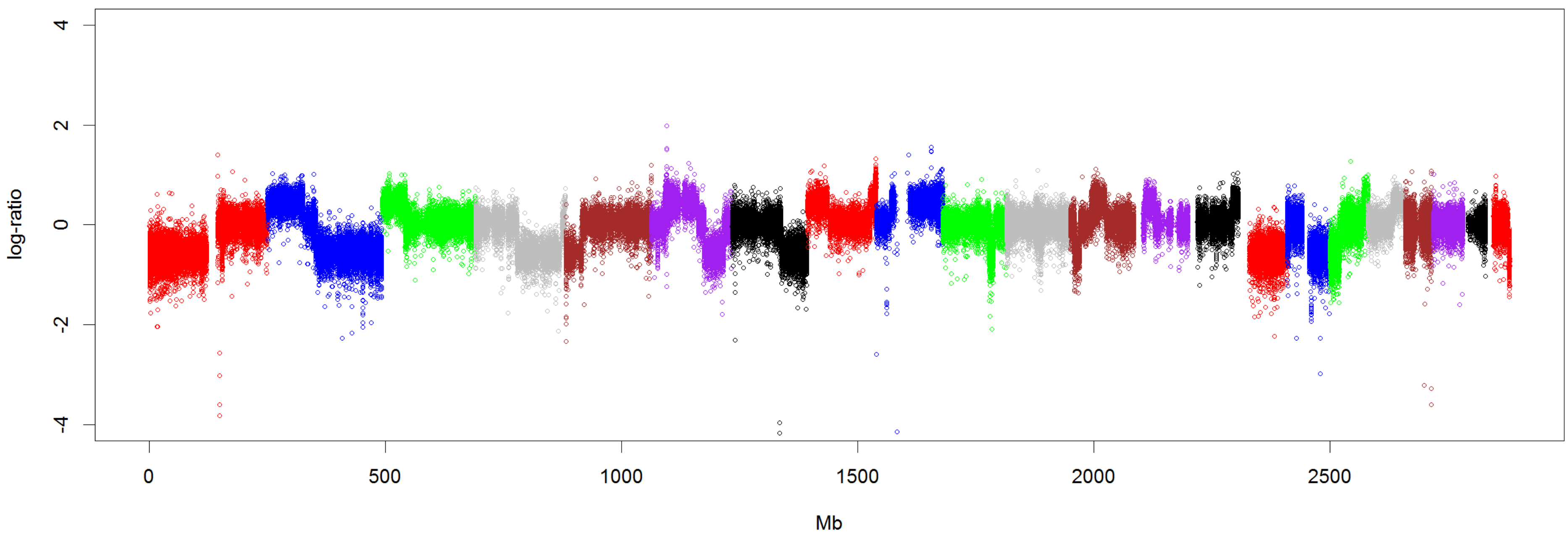

Patient Sample 26

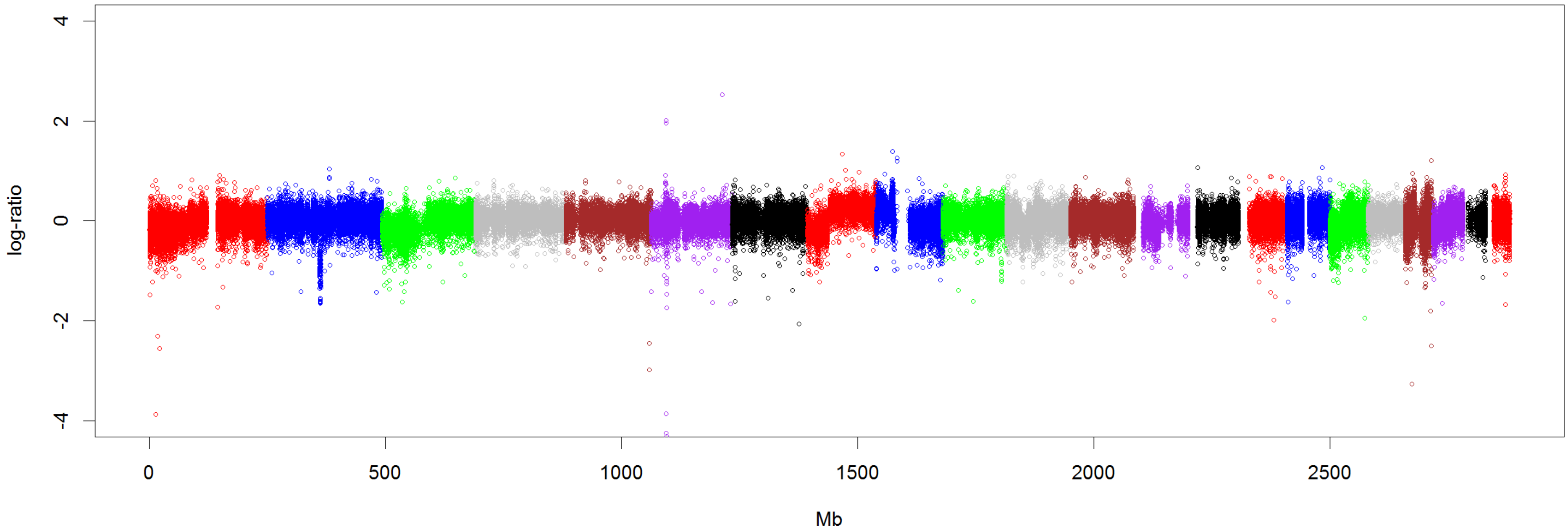

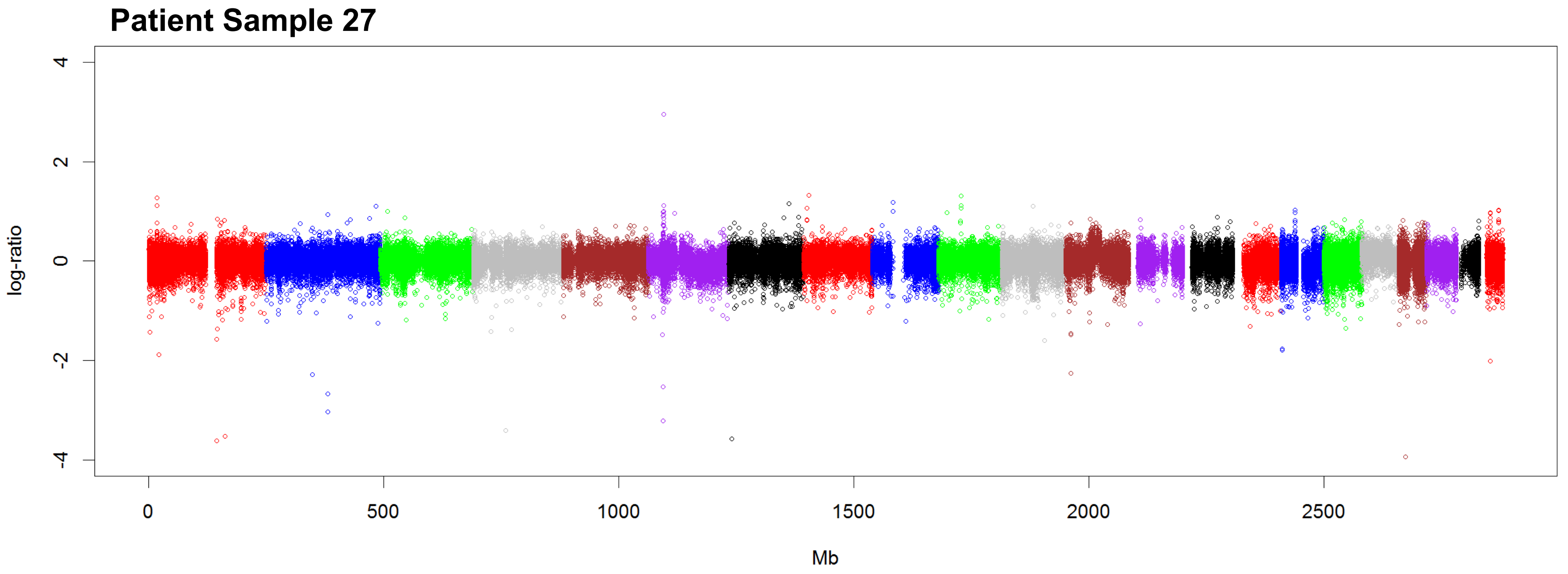
Patient Sample 27
log-ratio
4
2
0
-2
-4
0
500
1000
1500
2000
2500
Mb

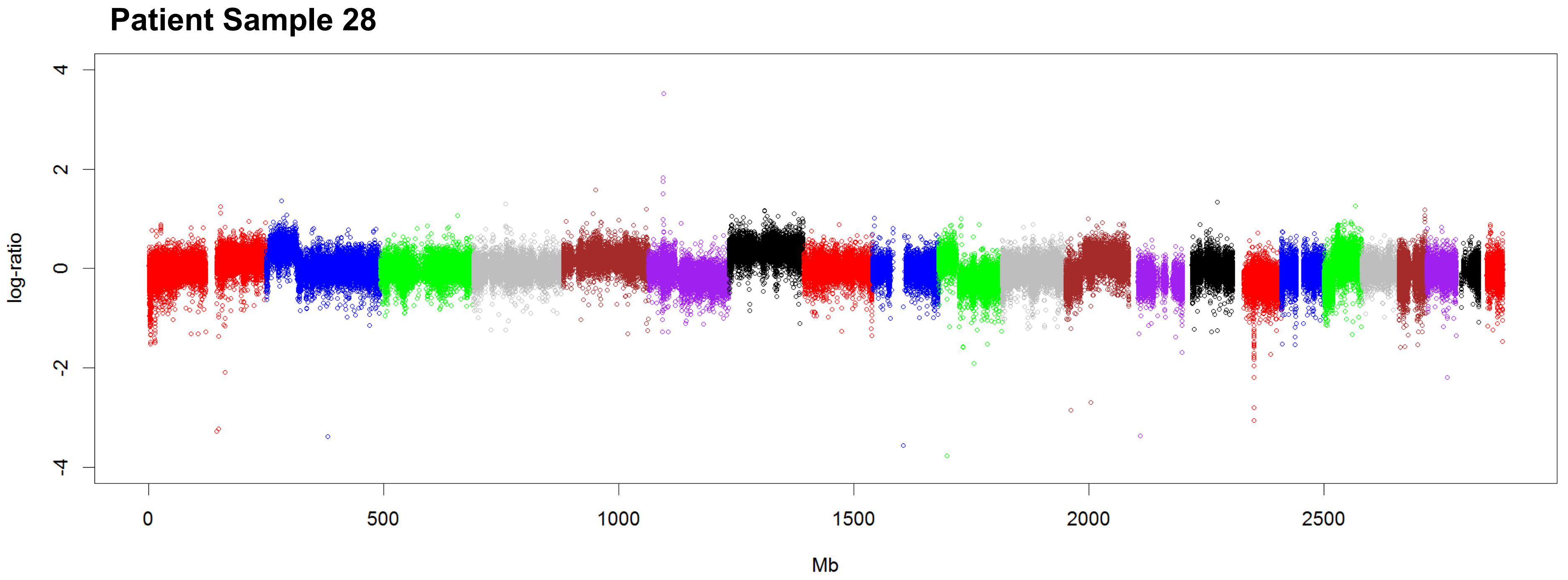
Patient Sample 28
log-ratio
4
2
0
-2
-4
0
500
1000
1500
2000
2500
Mb

Patient Sample 29

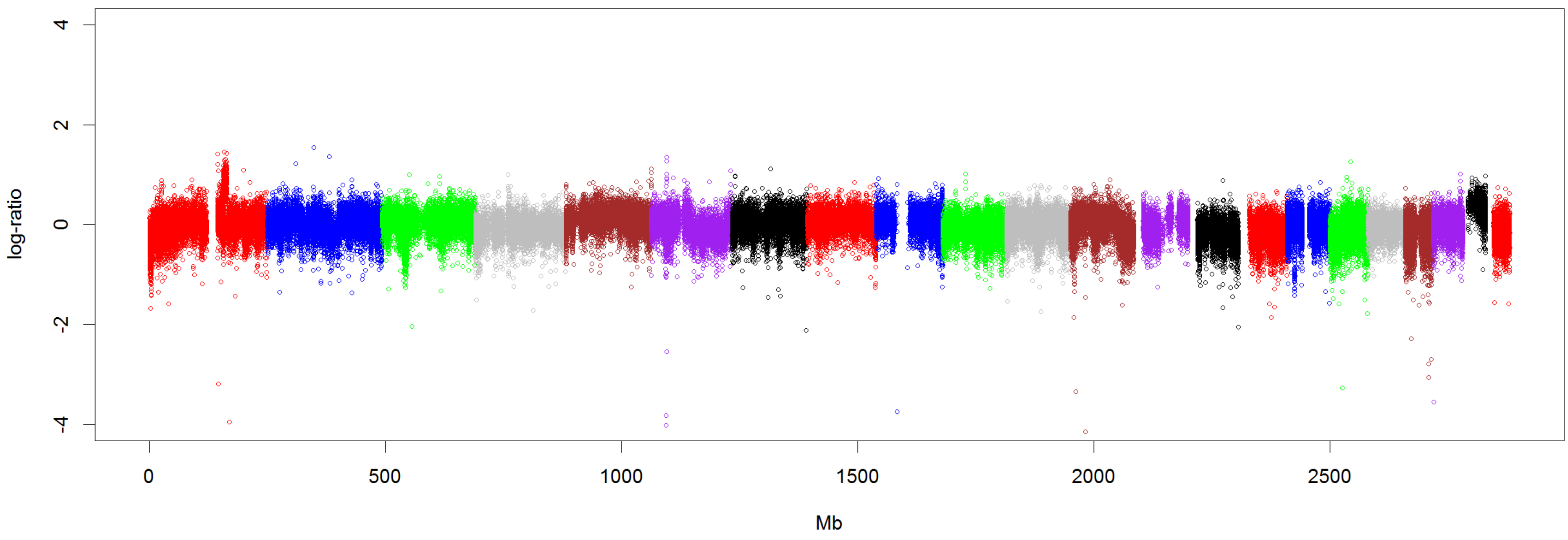

Patient Sample 30

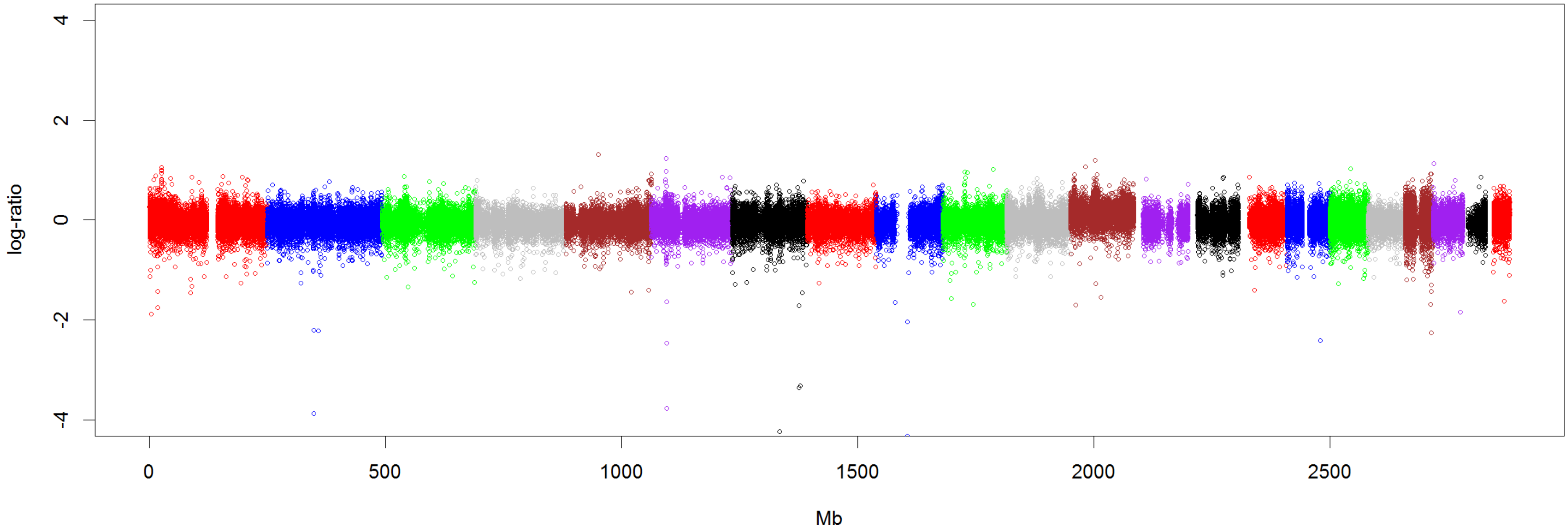

Patient Sample 31

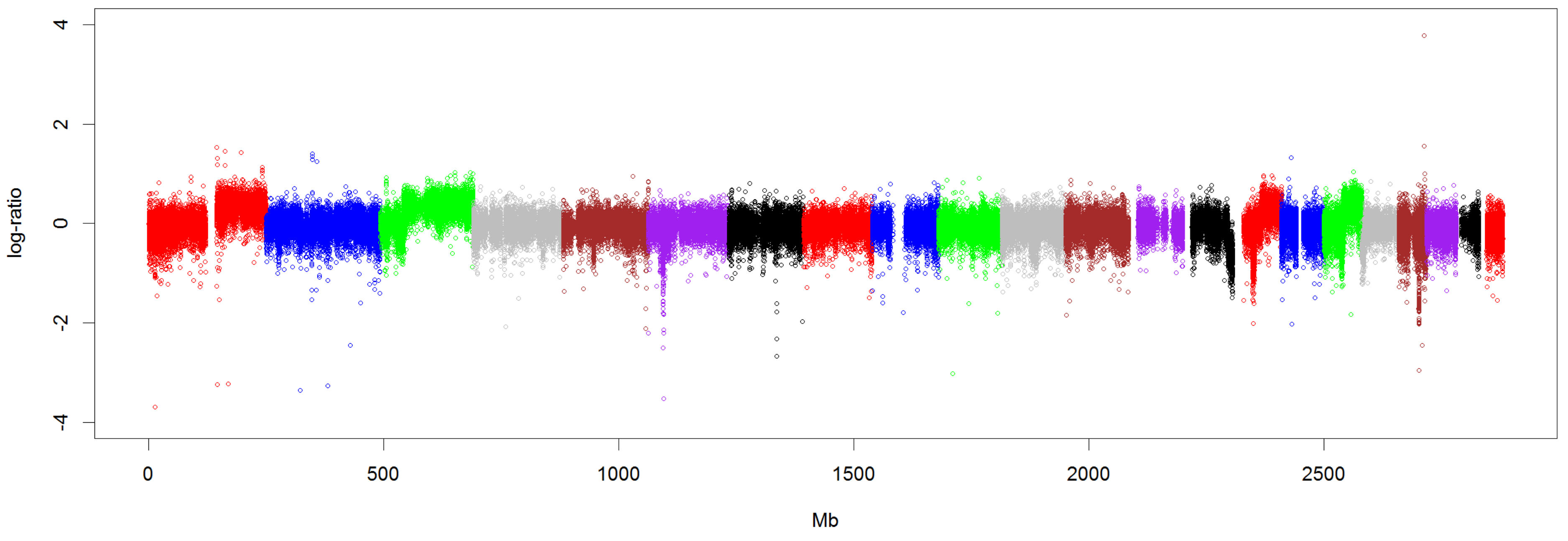

Patient Sample 32

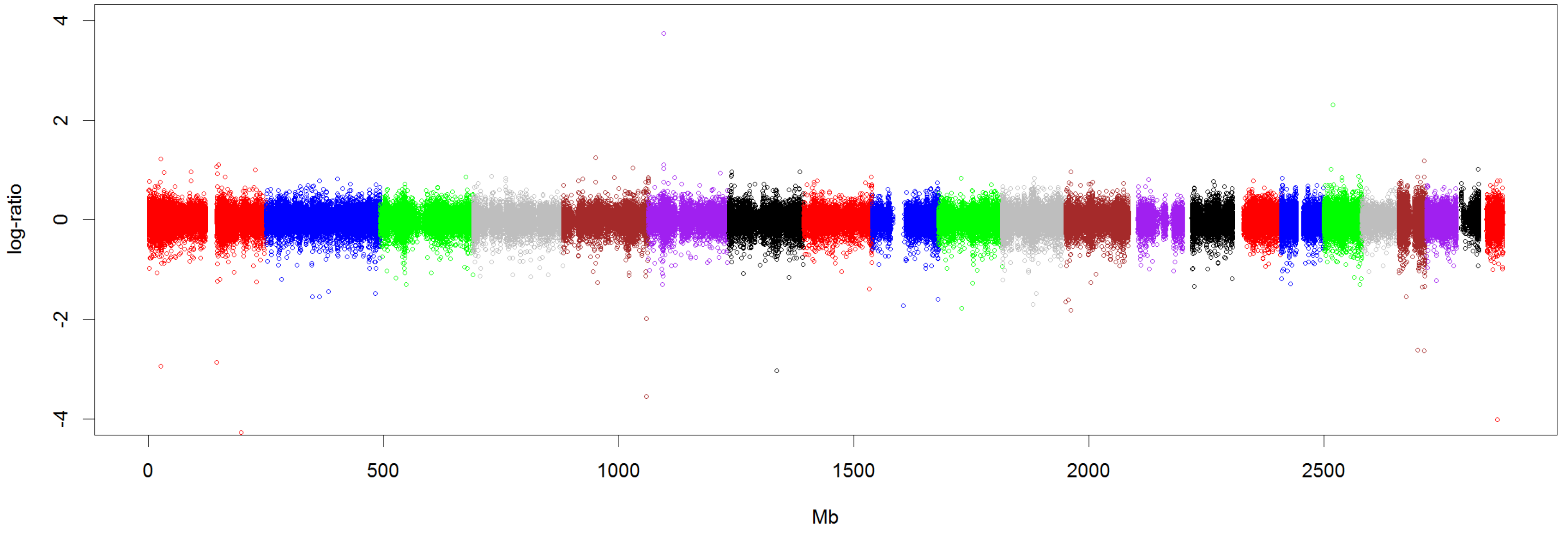

Patient Sample 33

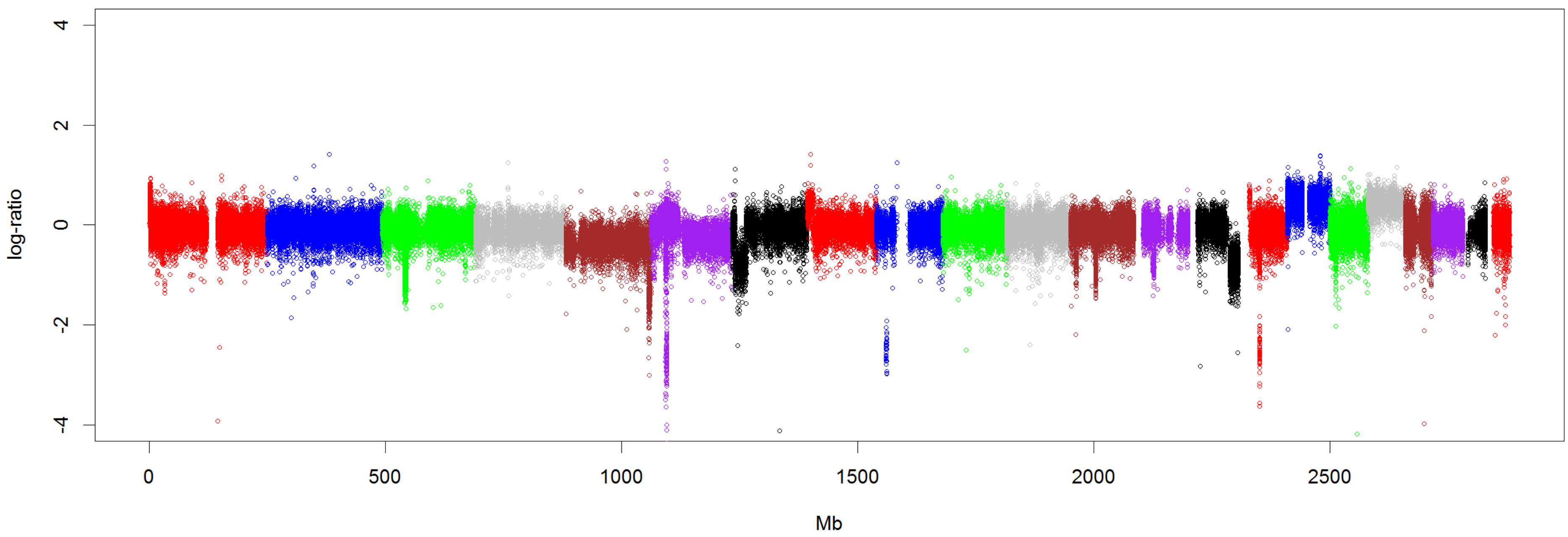

Patient Sample 34

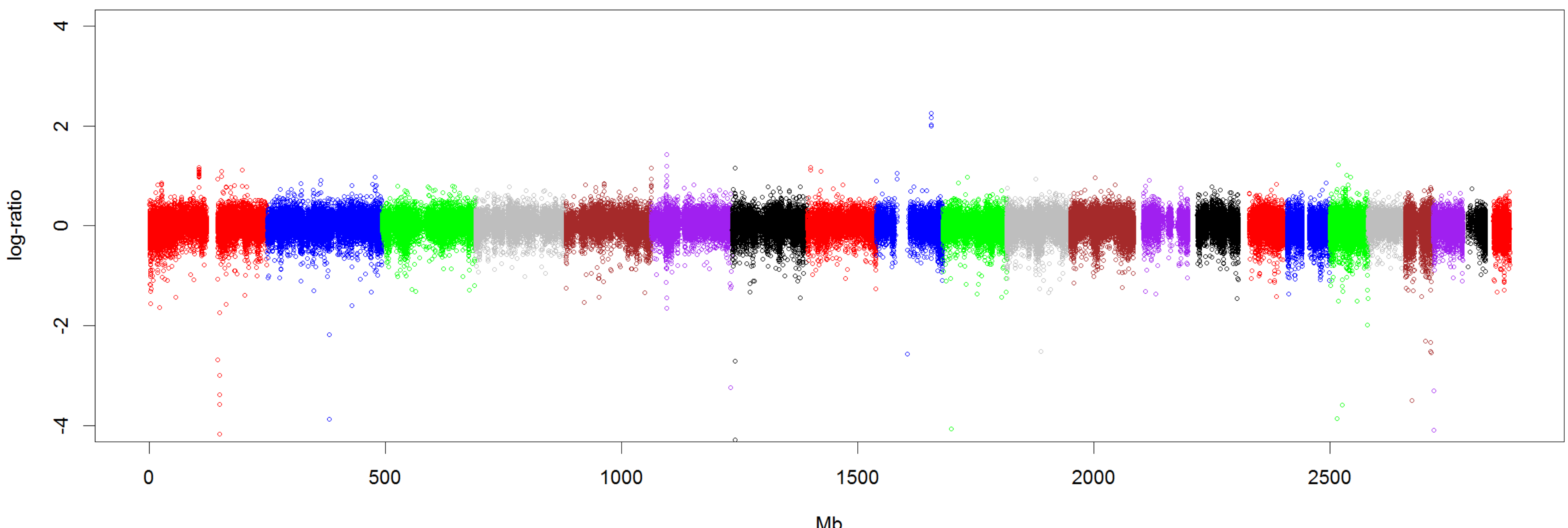

Patient Sample 35

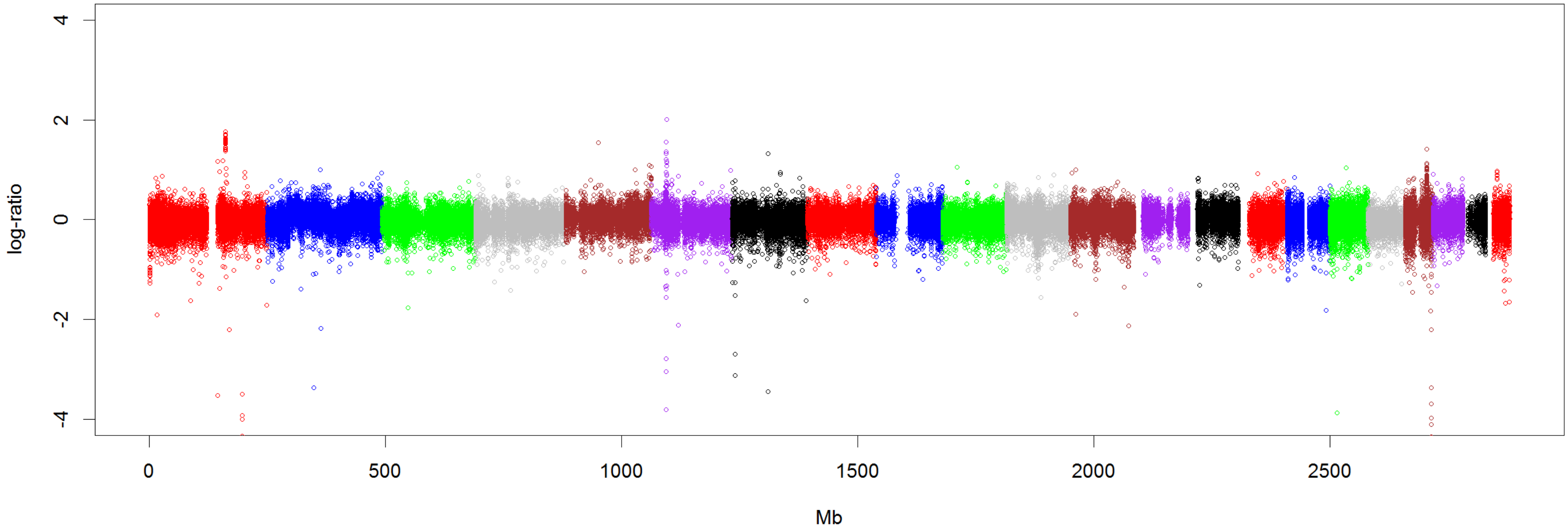

Patient Sample 36

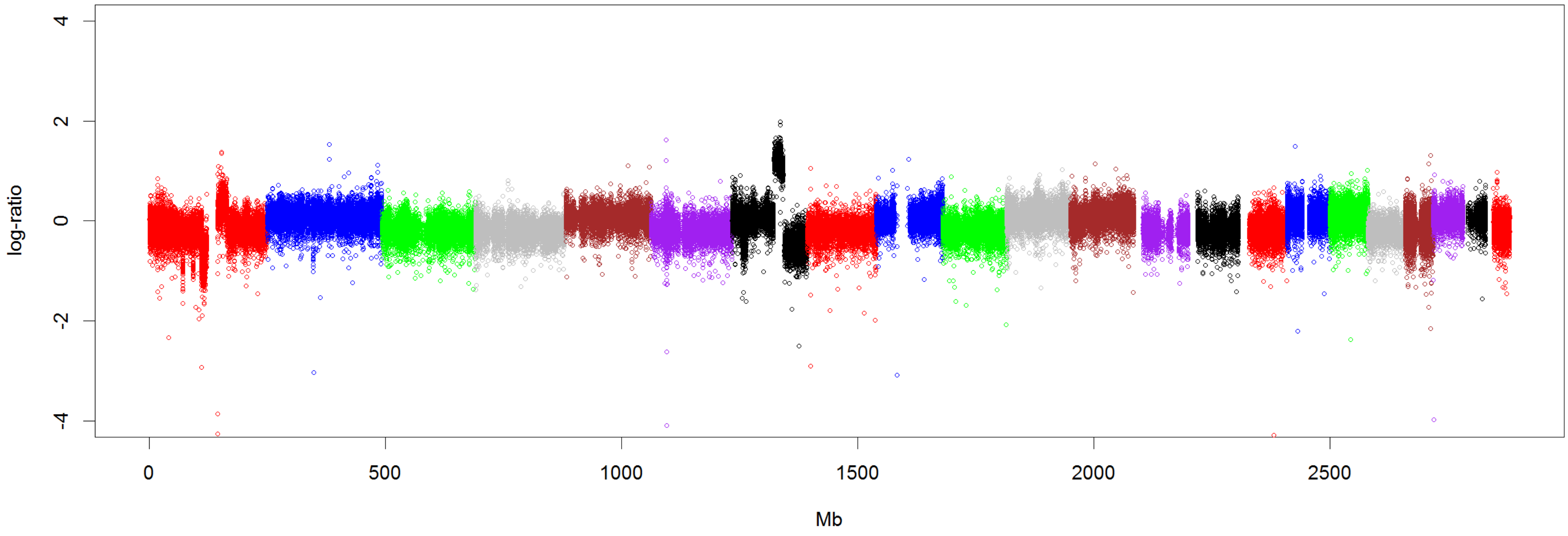

Patient Sample 37

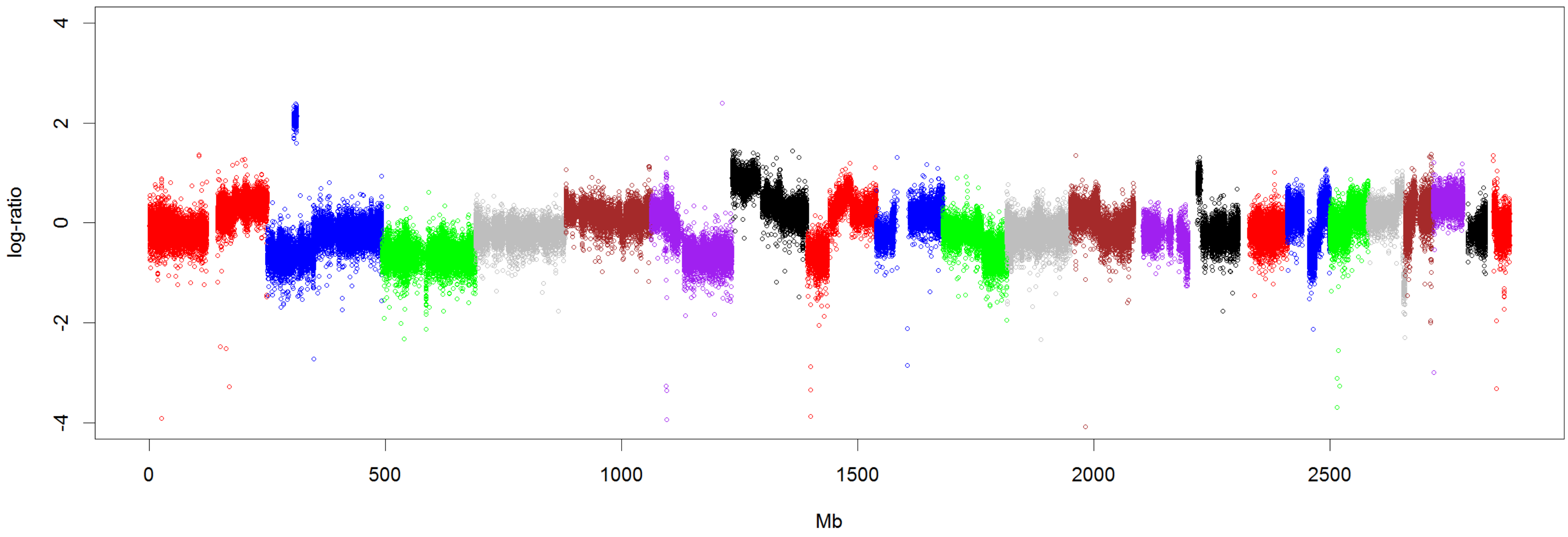

Patient Sample 38

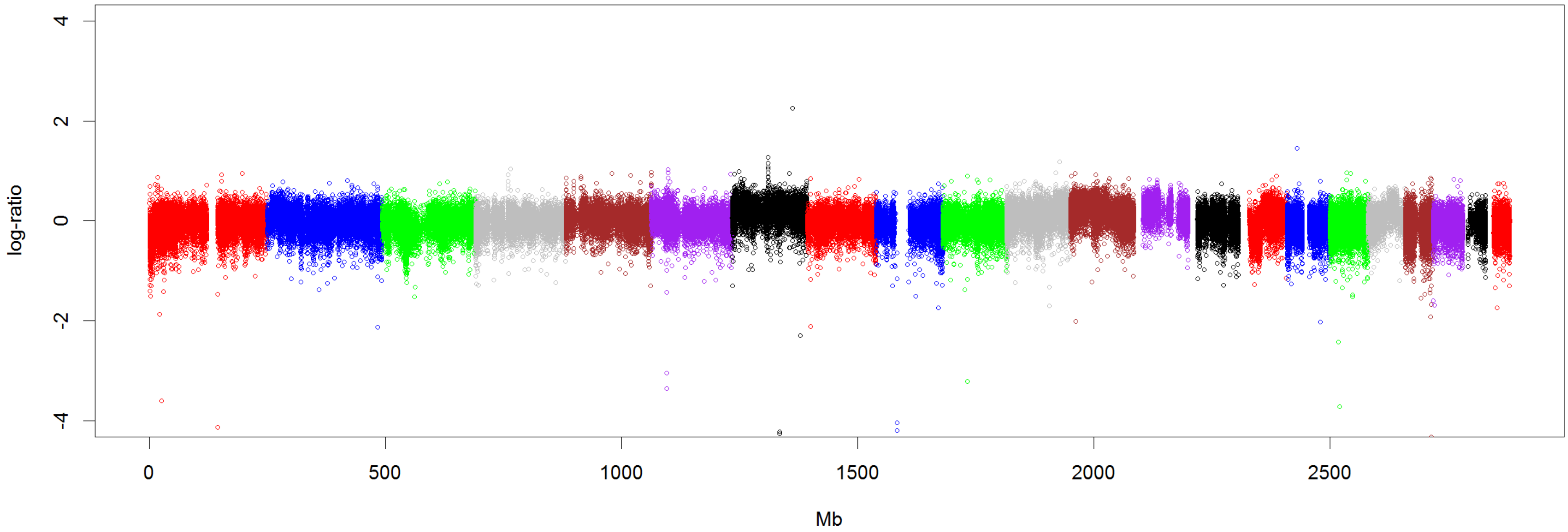

Patient Sample 39

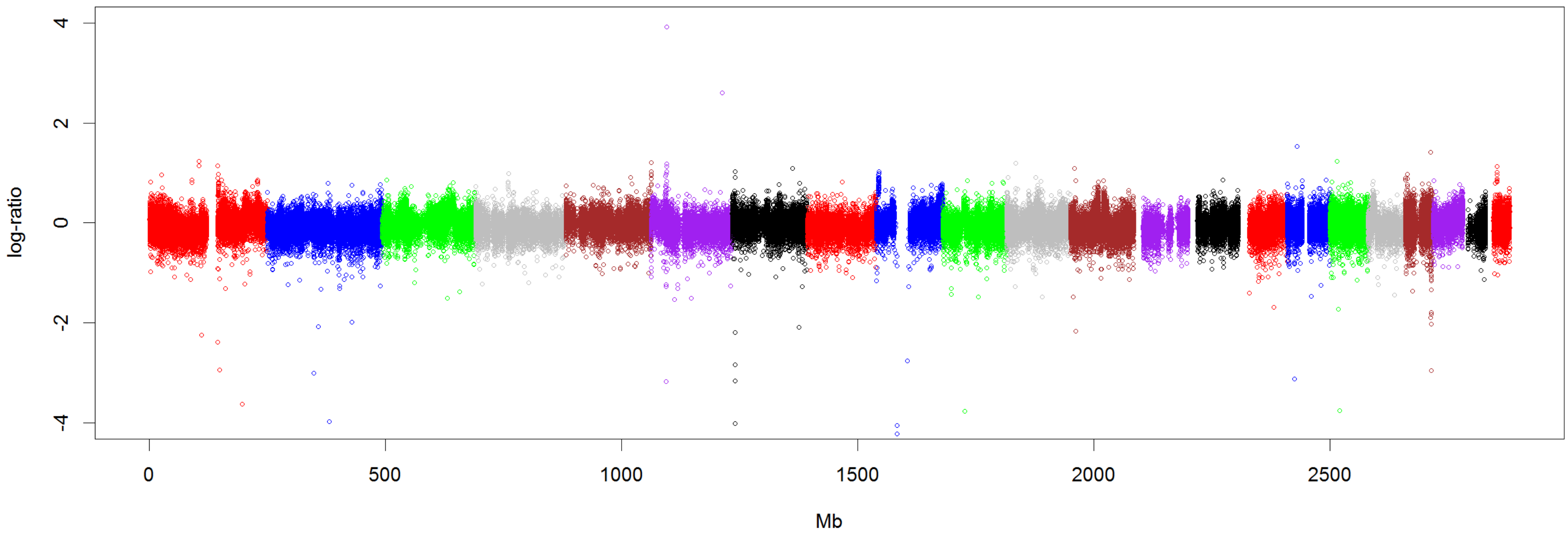

Patient Sample 40

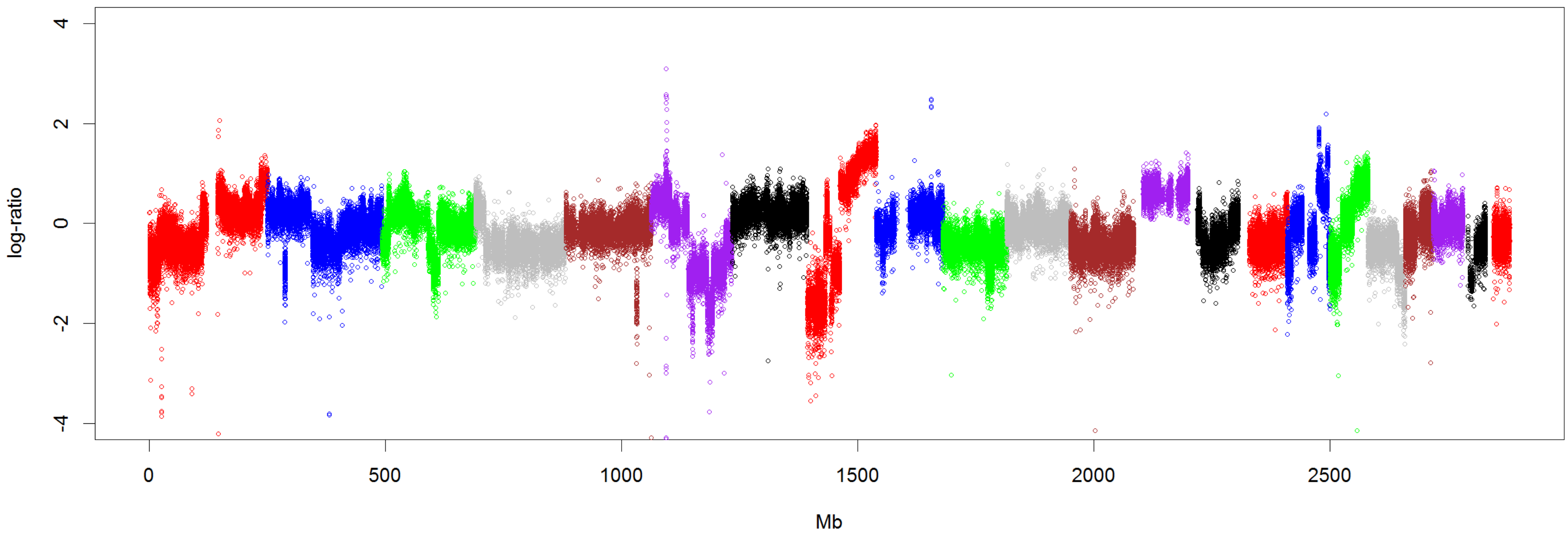

Patient Sample 41

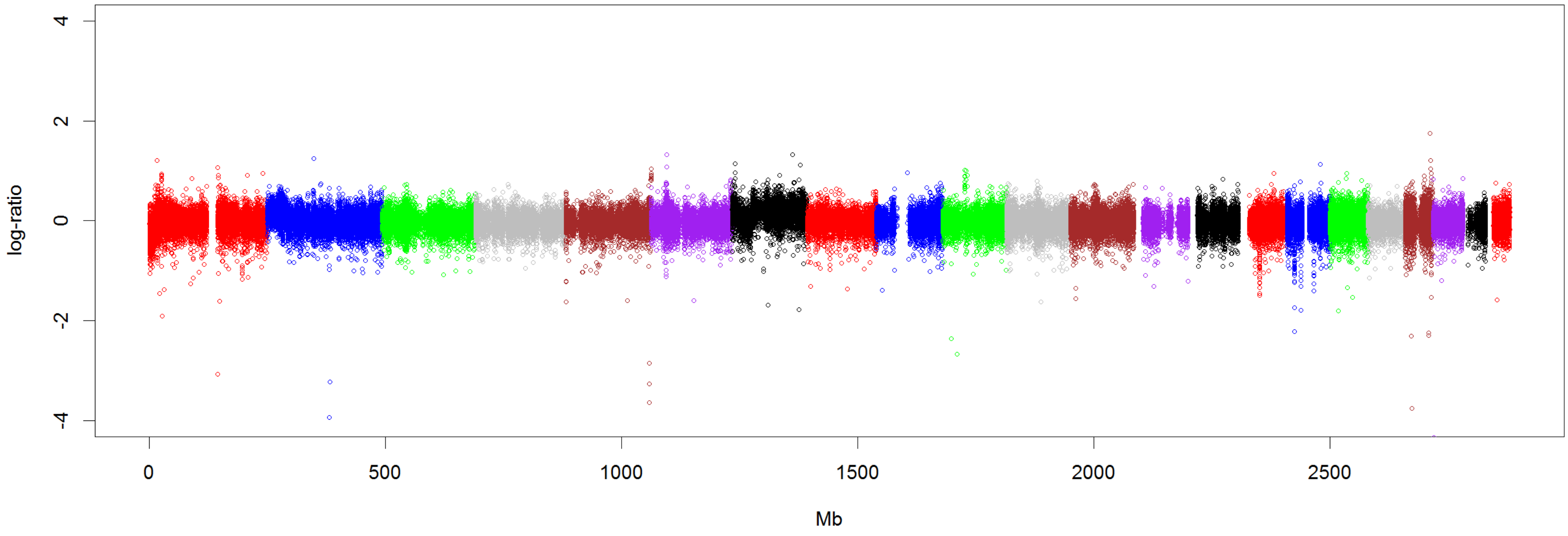

Patient Sample 42

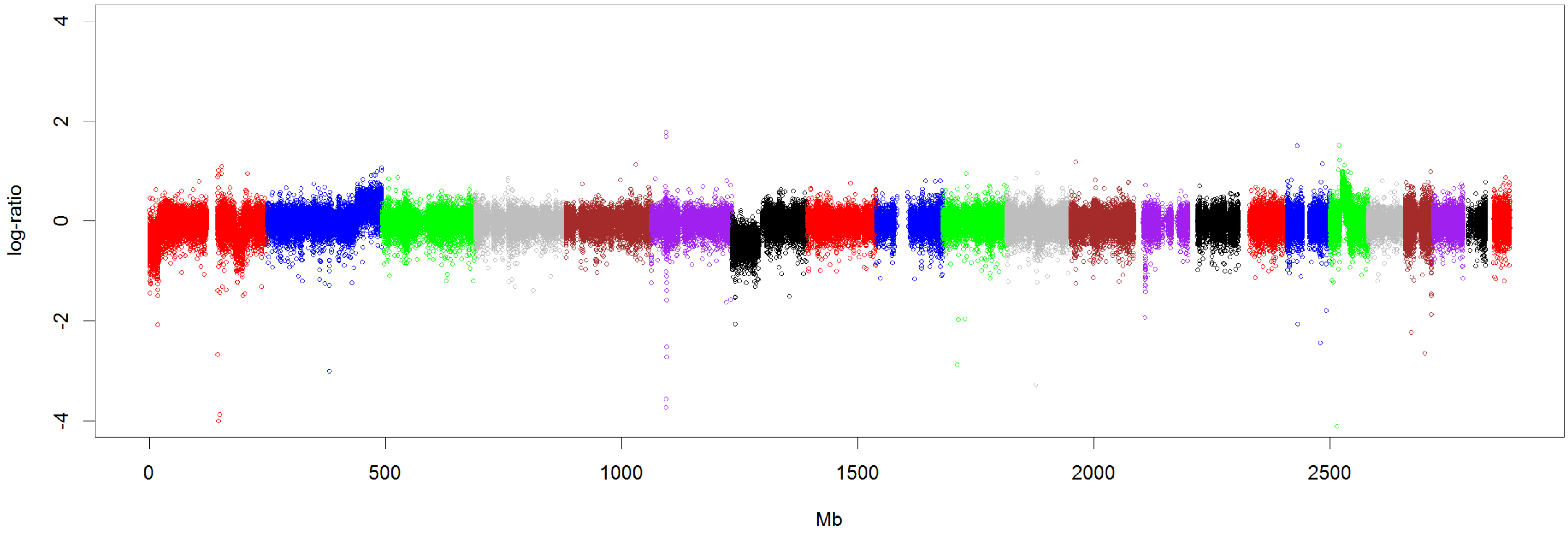

Patient Sample 43

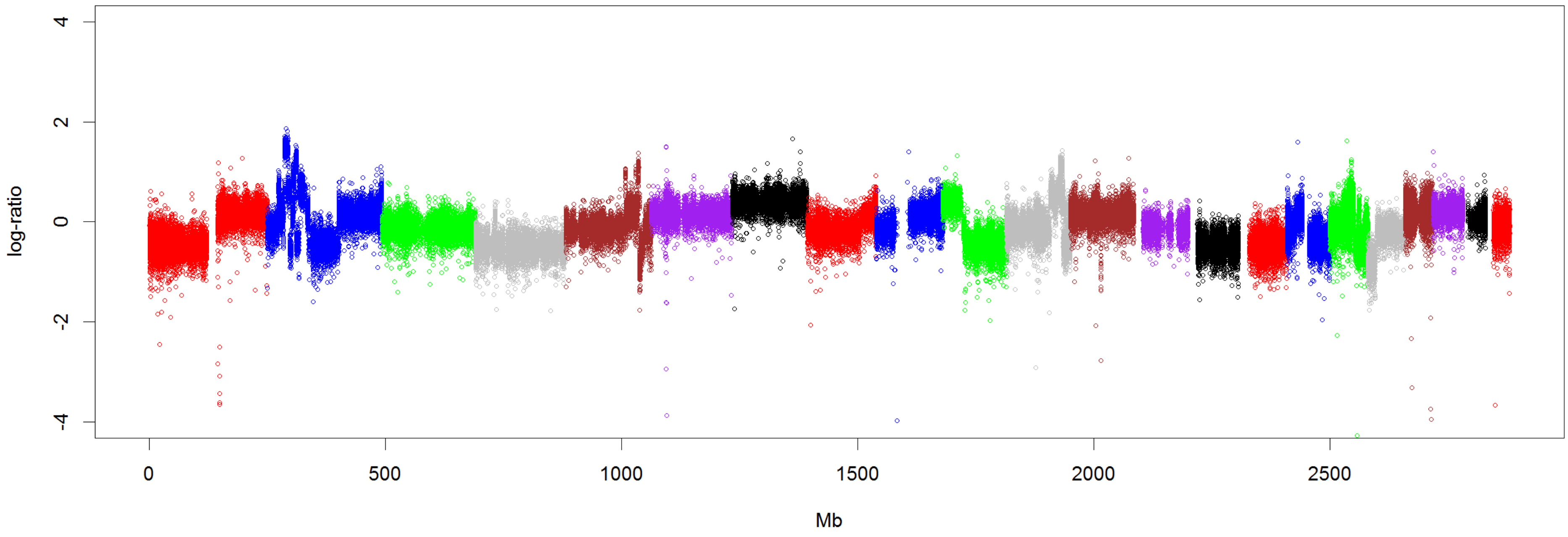

Patient Sample 44

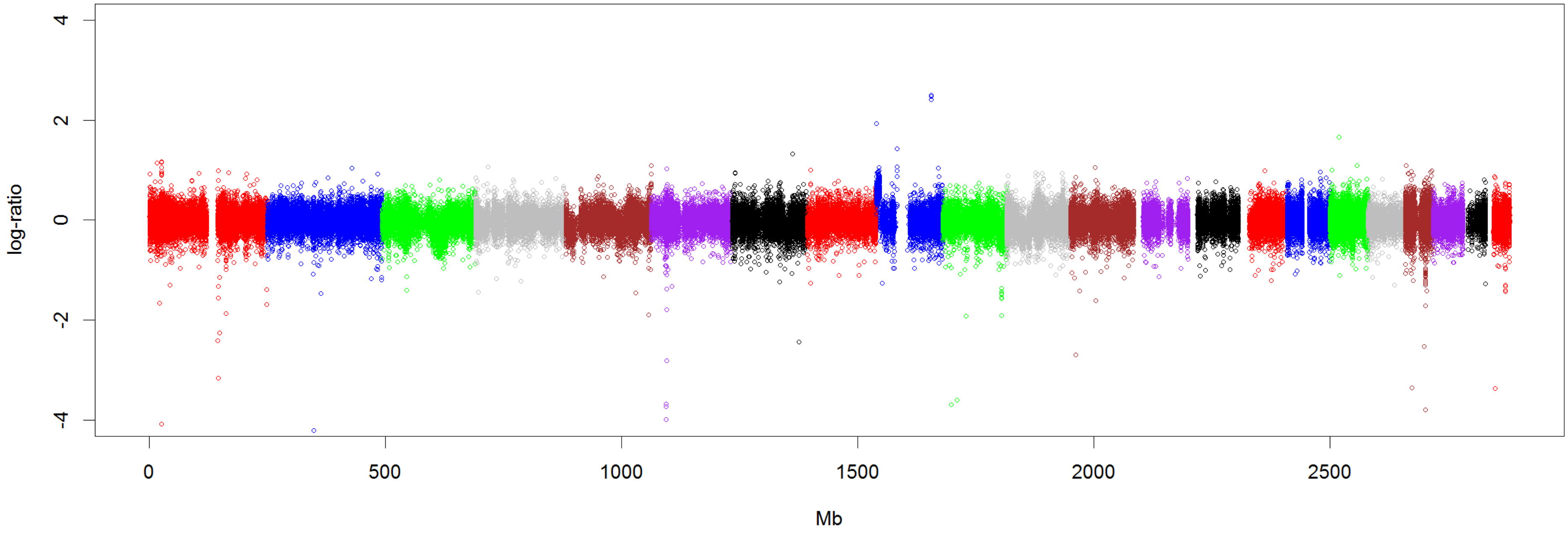

Patient Sample 45

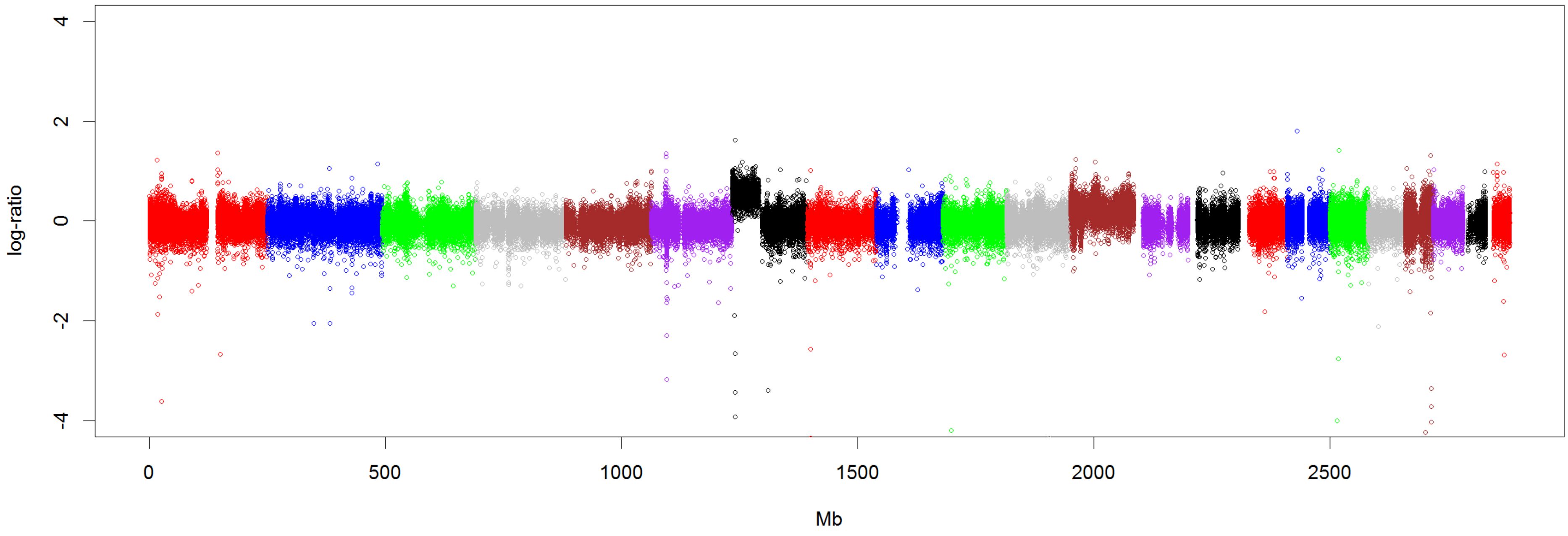

Patient Sample 46

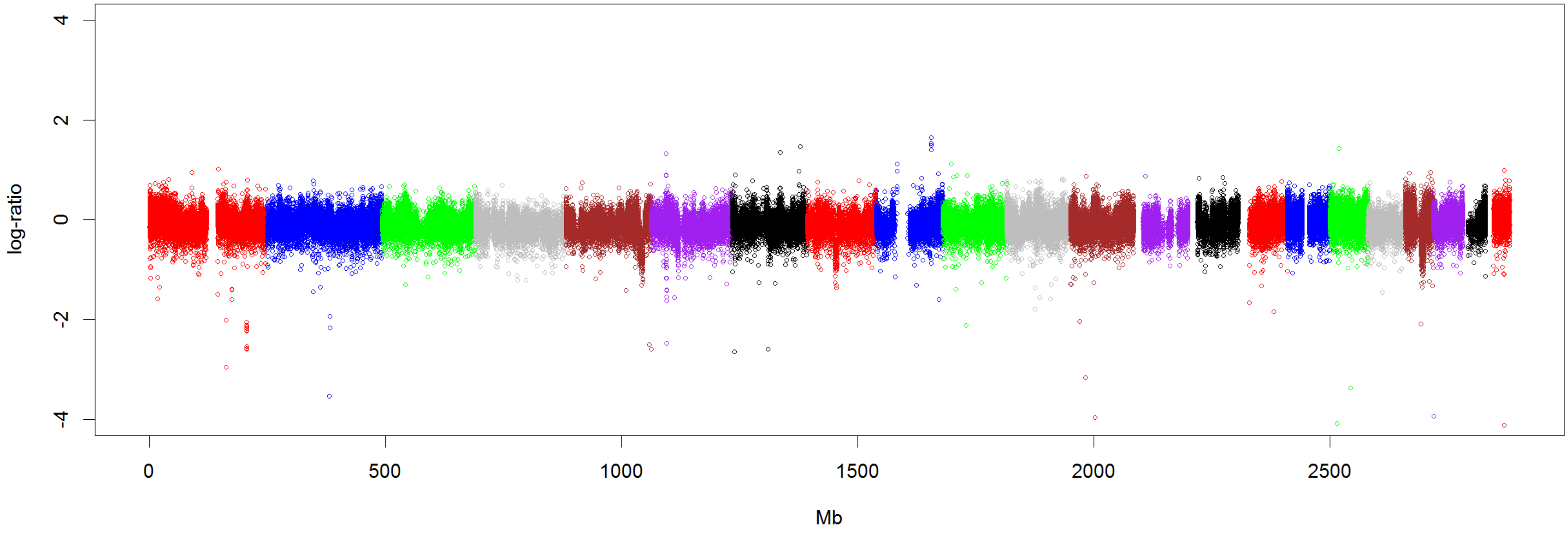

Patient Sample 47

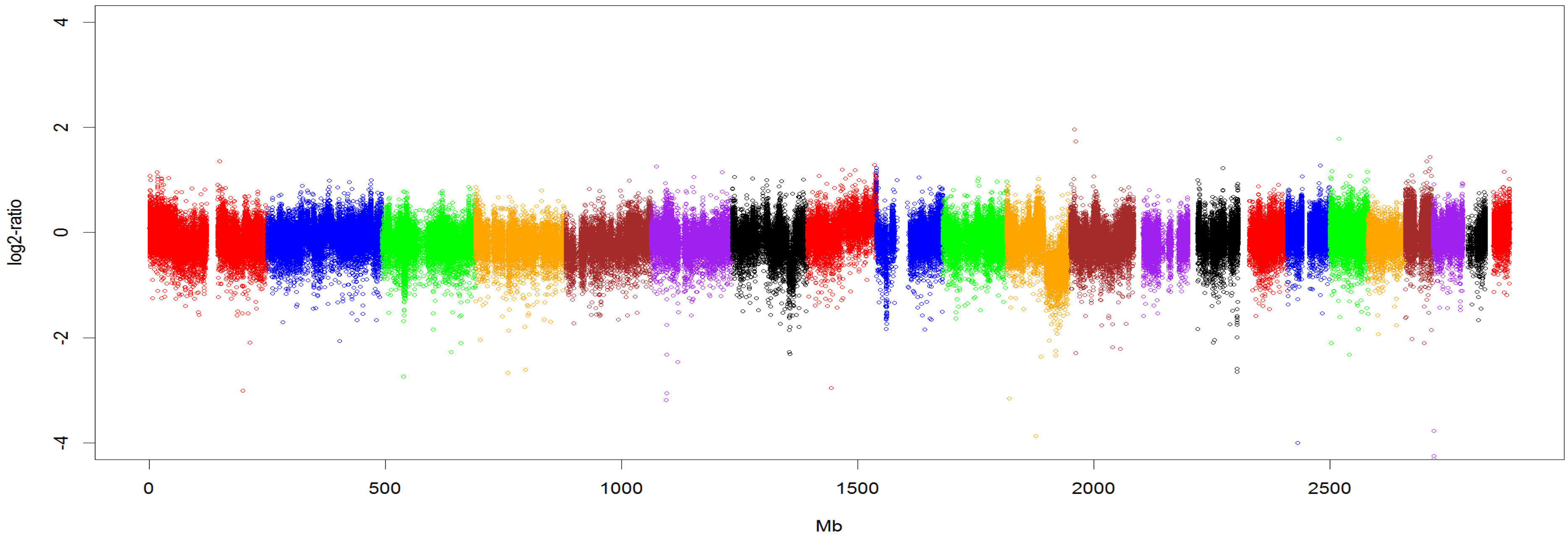

Patient Sample 48

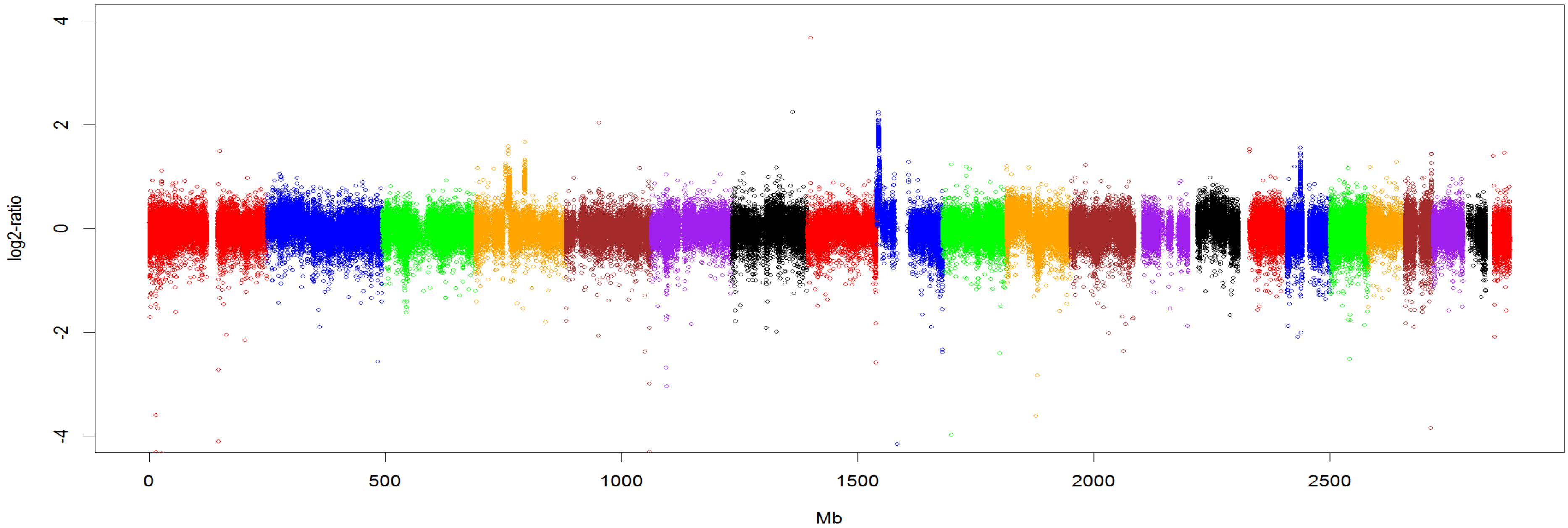

Patient Sample 49

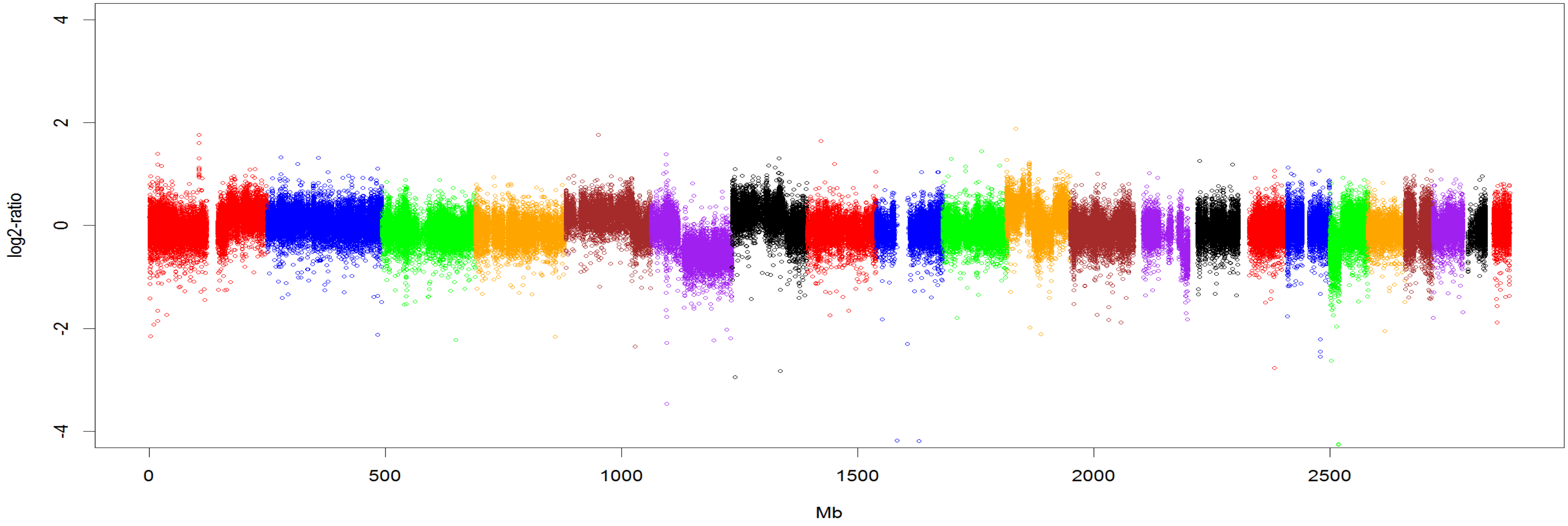

Patient Sample 50

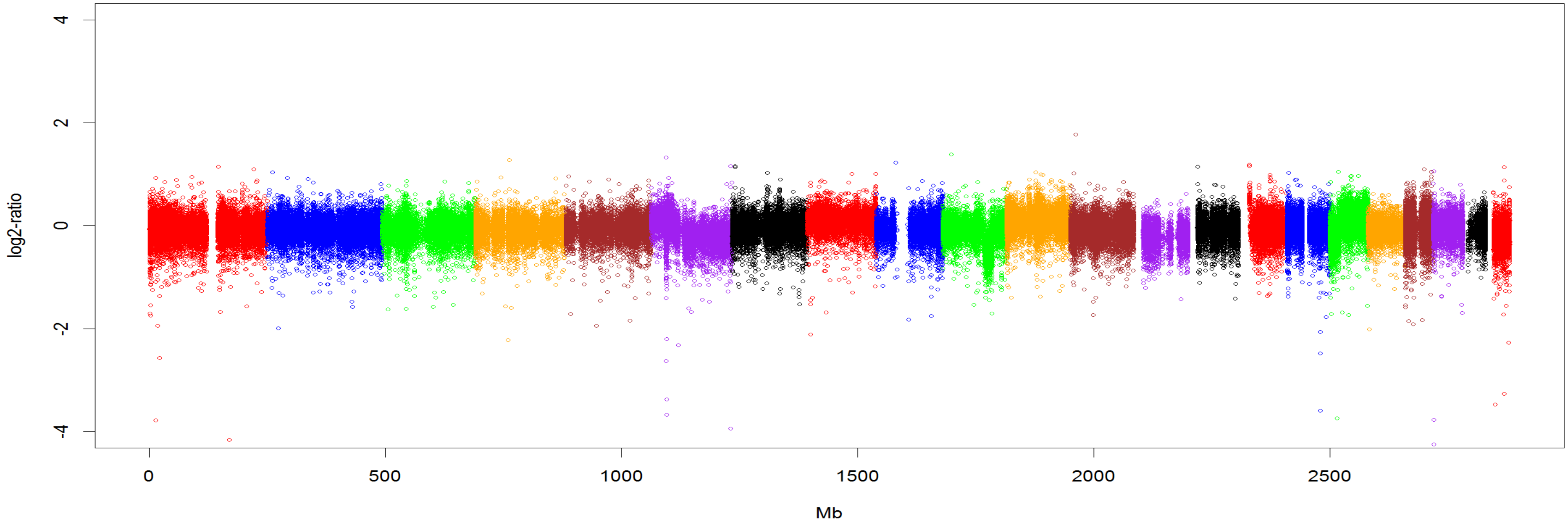

Patient Sample 51

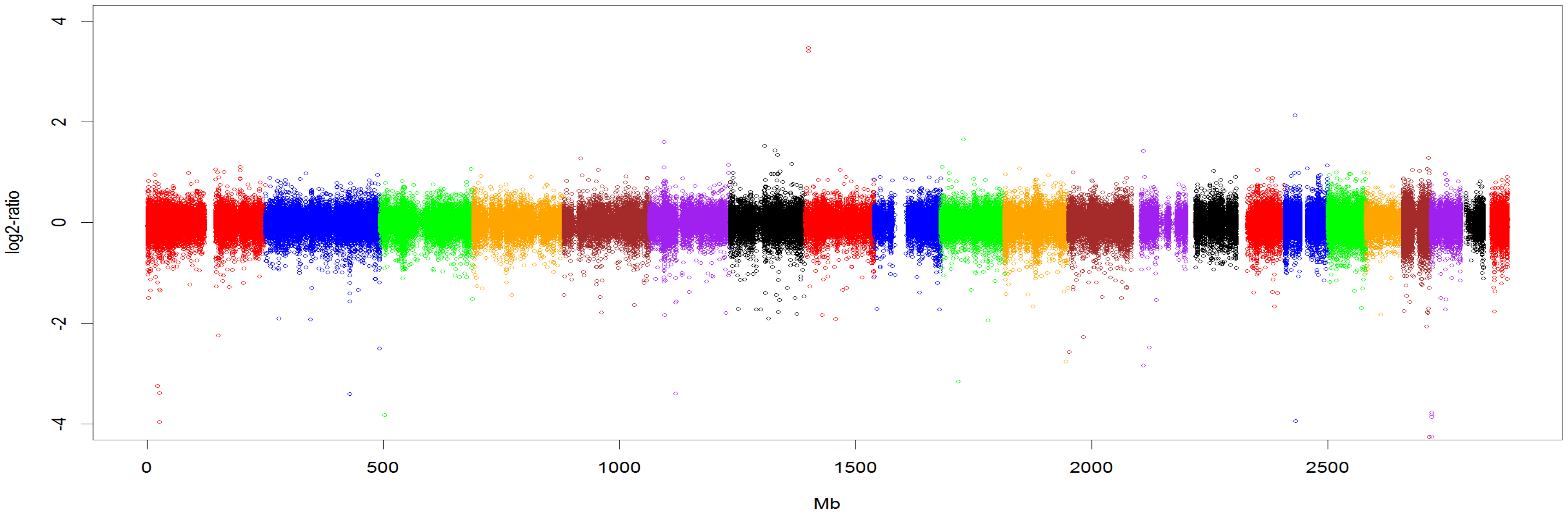

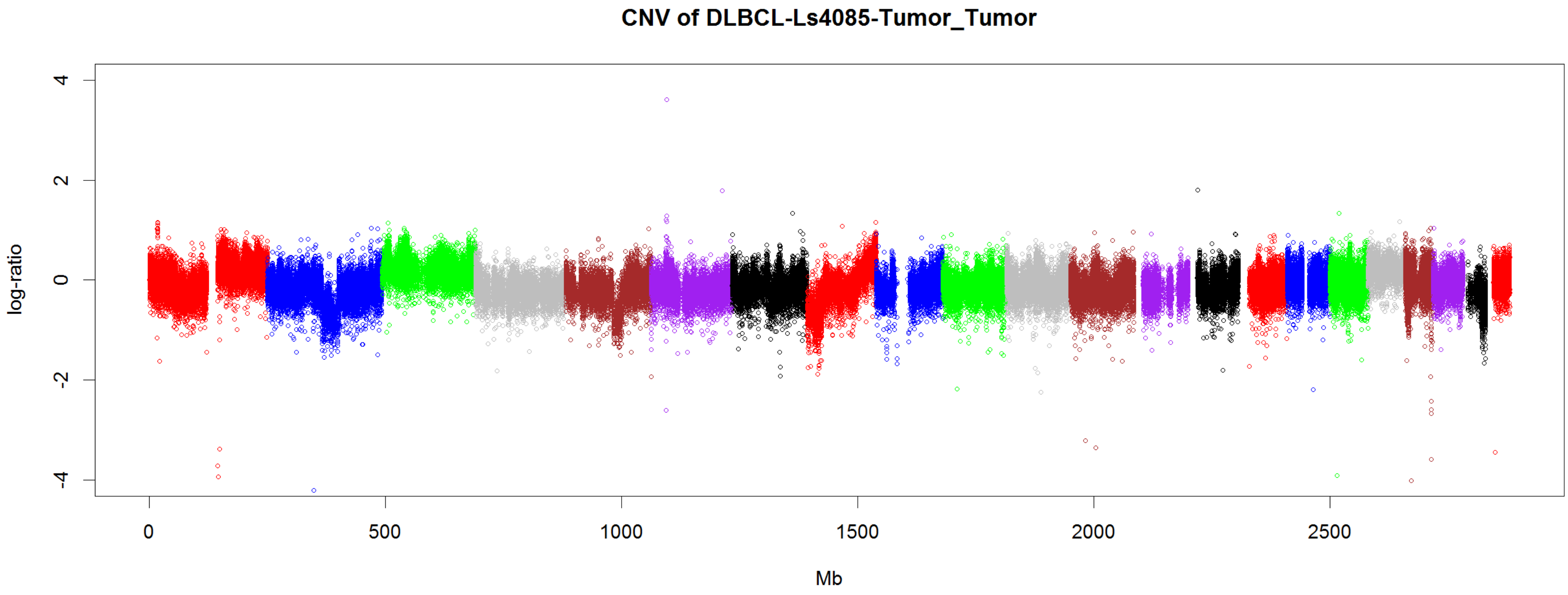
CNV of DLBCL-Ls4085-Tumor_Tumor
log-ratio
4
2
0
-2
-4
0
500
1000
1500
2000
2500
Mb

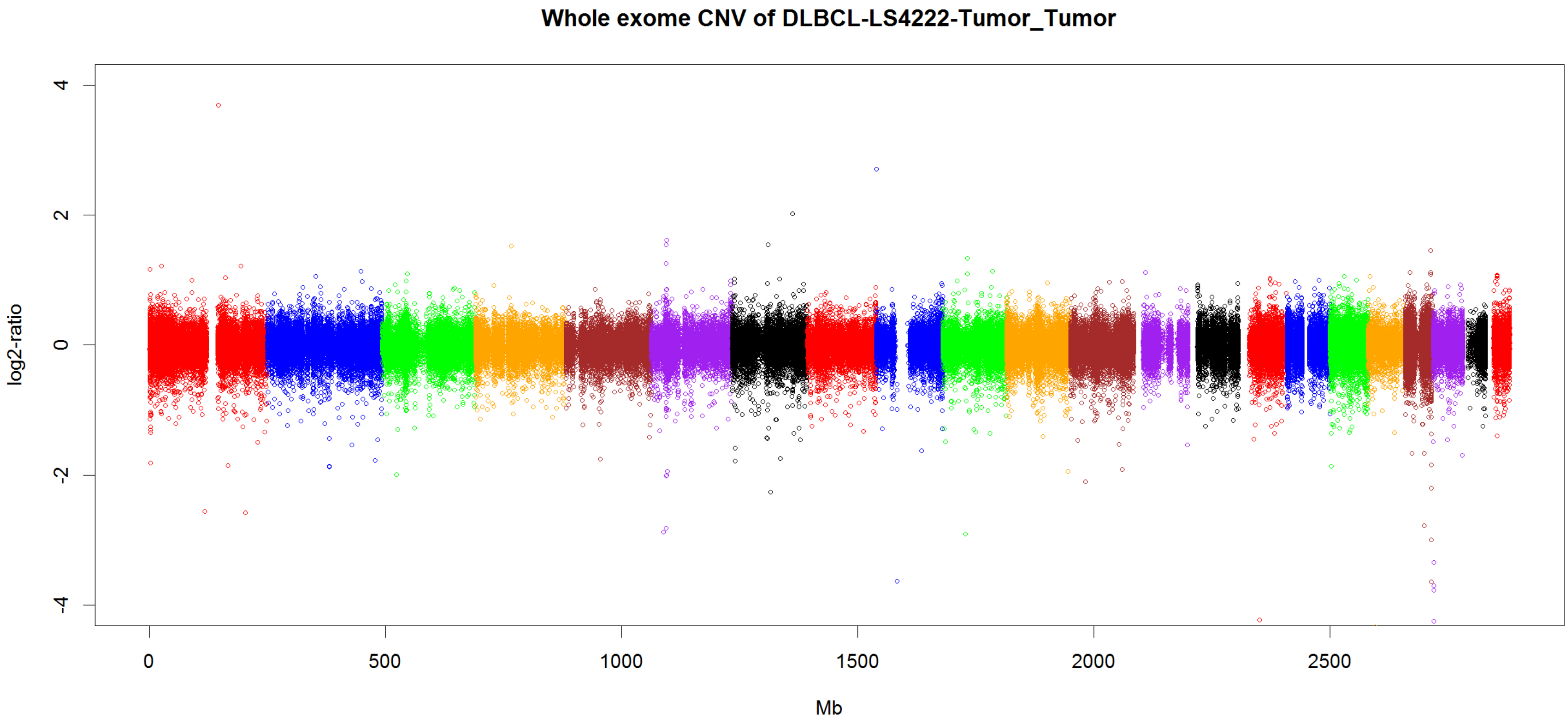
Whole exome CNV of DLBCL-LS4222-Tumor_Tumor
log2-ratio
4
2
0
-2
-4
0
500
1000
1500
2000
2500
Mb

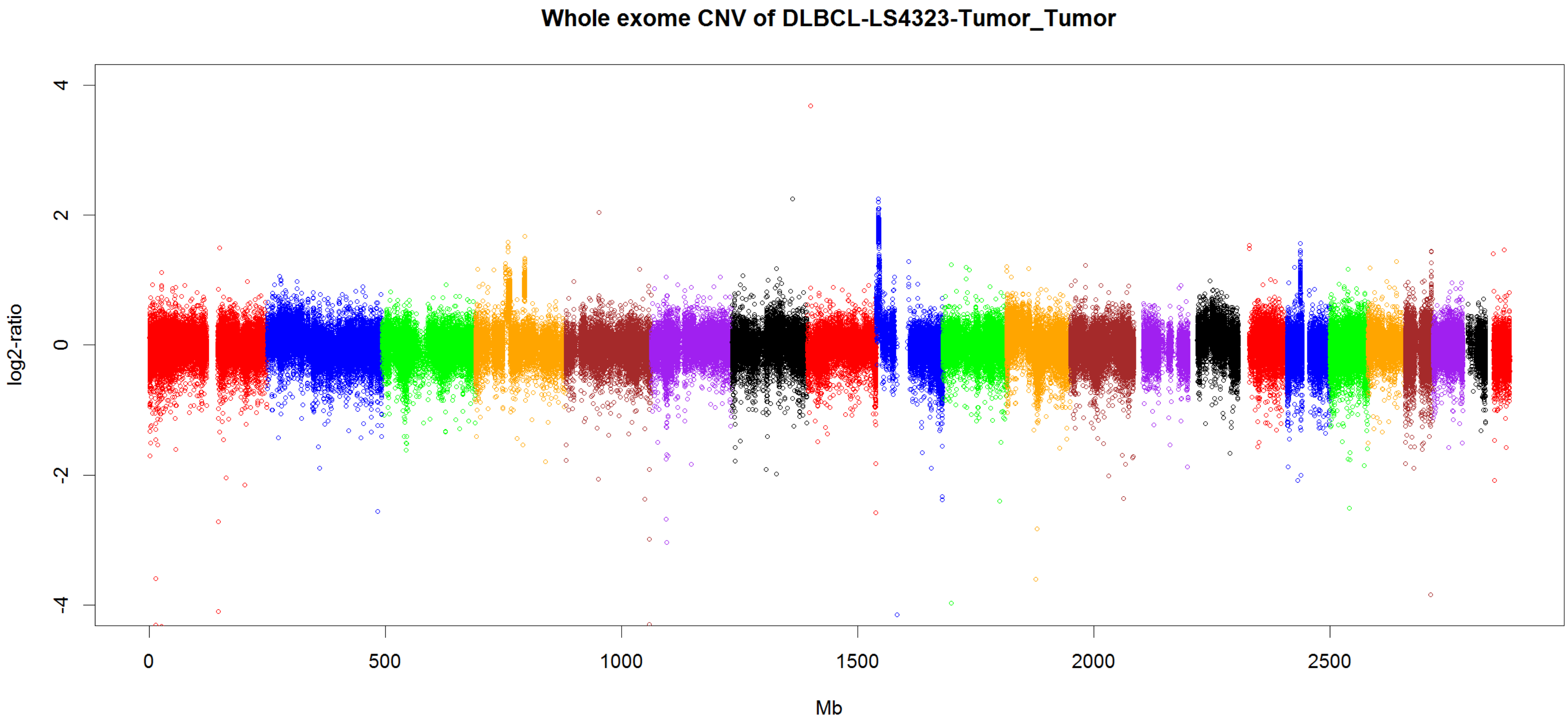
Whole exome CNV of DLBCL-LS4323-Tumor_Tumor
log2-ratio
4
2
0
-2
-4
0
500
1000
1500
2000
2500
Mb

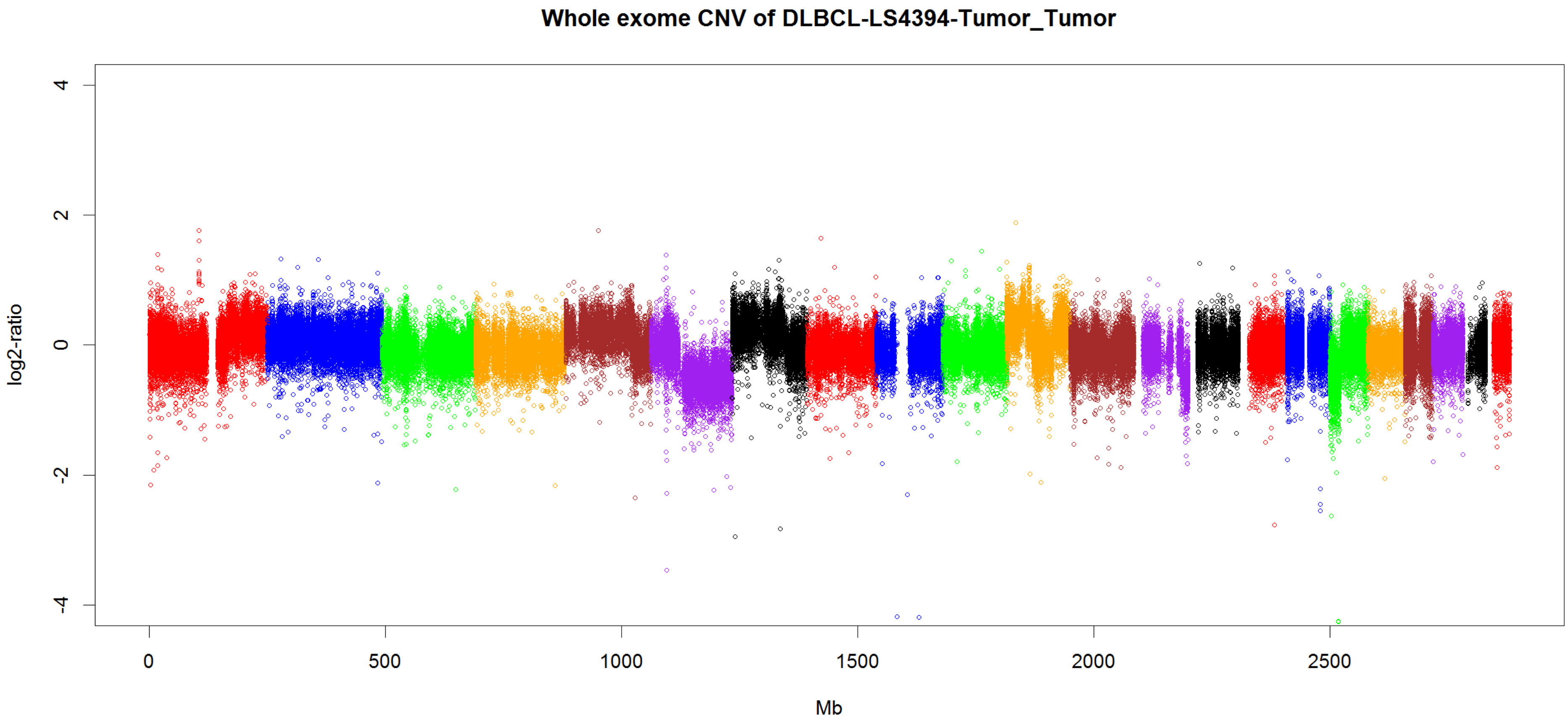
Whole exome CNV of DLBCL-LS4394-Tumor_Tumor
log2-ratio
4
2
0
-2
-4
0
500
1000
1500
2000
2500
Mb

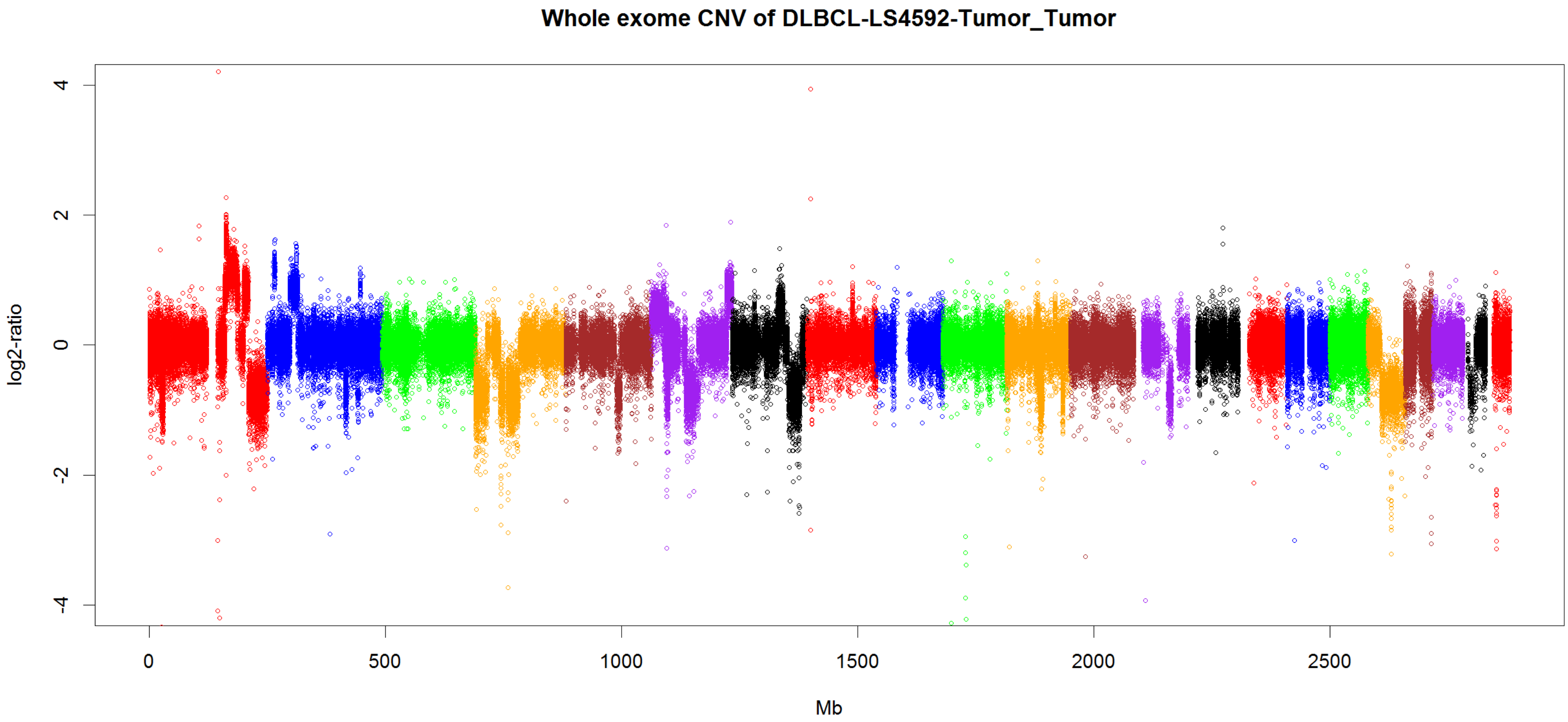
Whole exome CNV of DLBCL-LS4592-Tumor_Tumor
log2-ratio
4
2
0
-2
-4
0
500
1000
1500
2000
2500
Mb

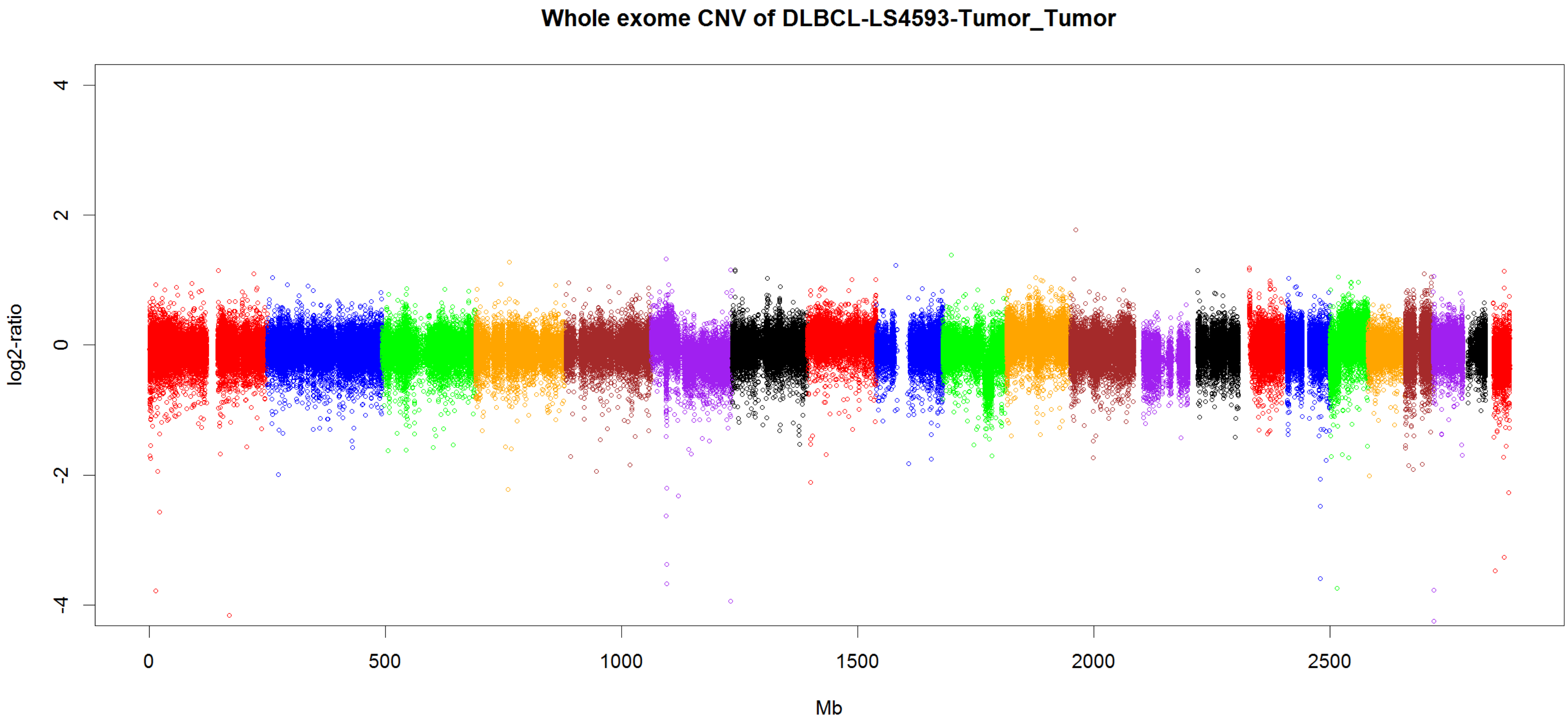

Whole exome CNV of DLBCL-LS4593-Tumor_Tumor
log2-ratio
4
2
0
-2
-4
0
500
1000
1500
2000
2500
Mb

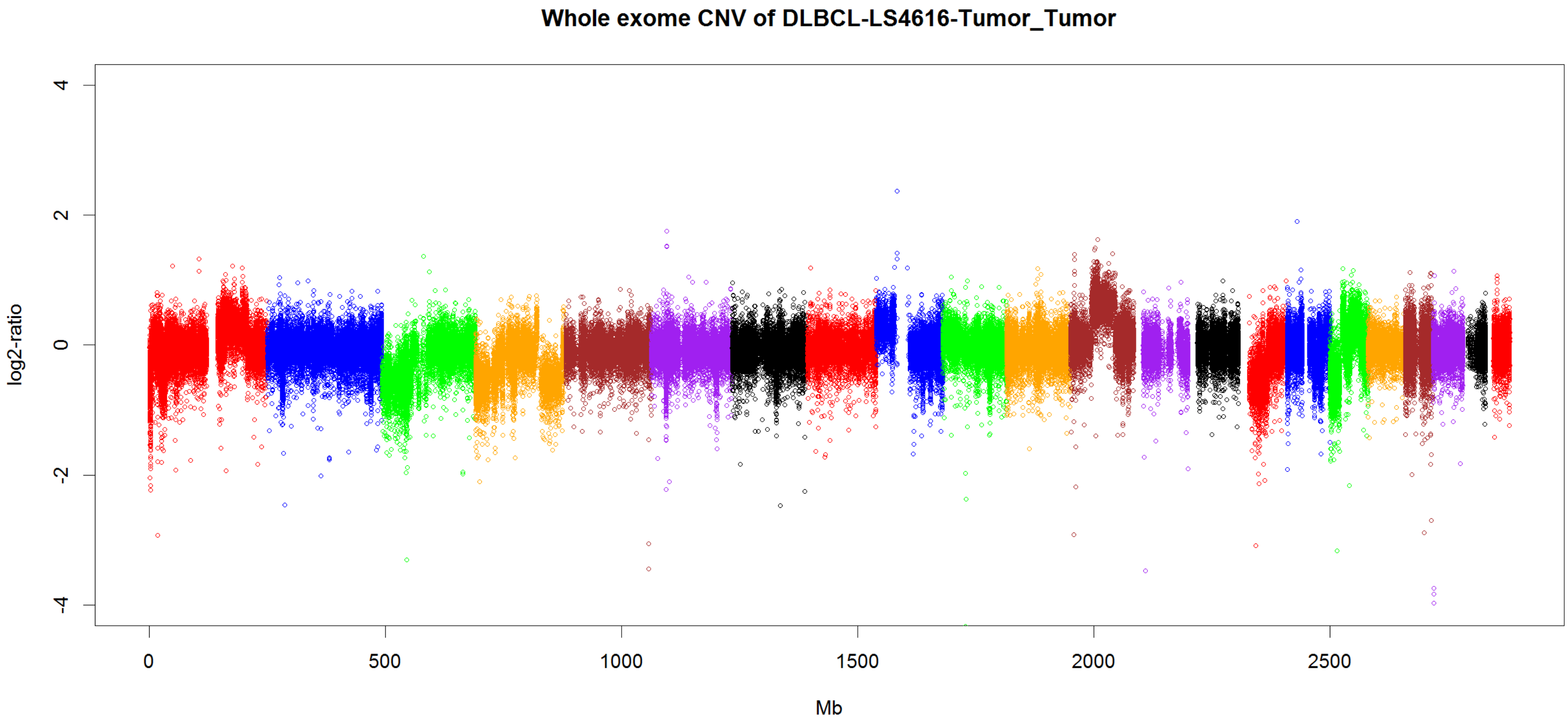
Whole exome CNV of DLBCL-LS4616-Tumor_Tumor
log2-ratio
4
2
0
-2
-4
0
500
1000
1500
2000
2500
Mb

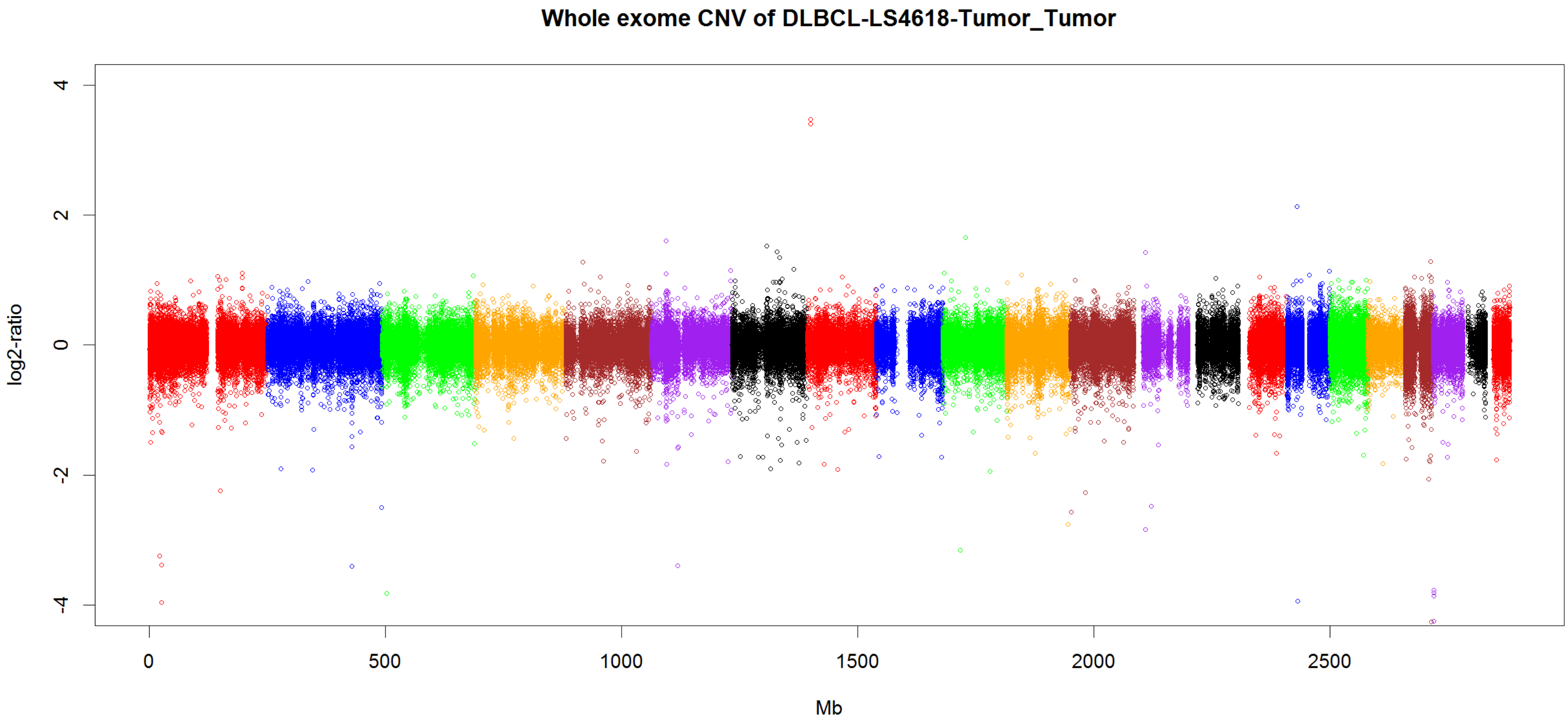
Whole exome CNV of DLBCL-LS4618-Tumor_Tumor
log2-ratio
4
2
0
-2
-4
0
500
1000
1500
2000
2500
Mb

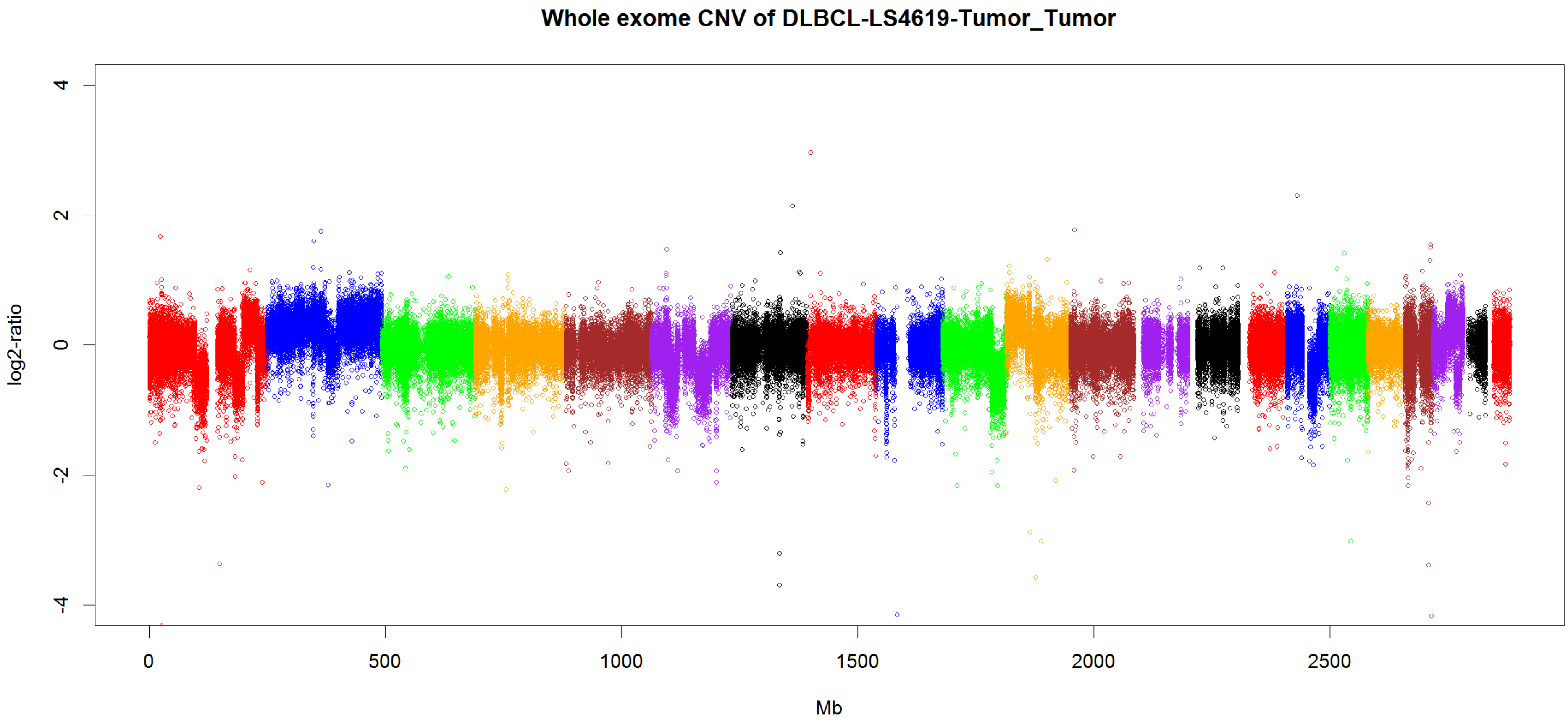
Whole exome CNV of DLBCL-LS4619-Tumor_Tumor
log2-ratio
4
2
0
-2
-4
0
500
1000
1500
2000
2500
Mb

Whole exome CNV of DLBCL-MAYO_DLBCL_234-Tumor_Tumor

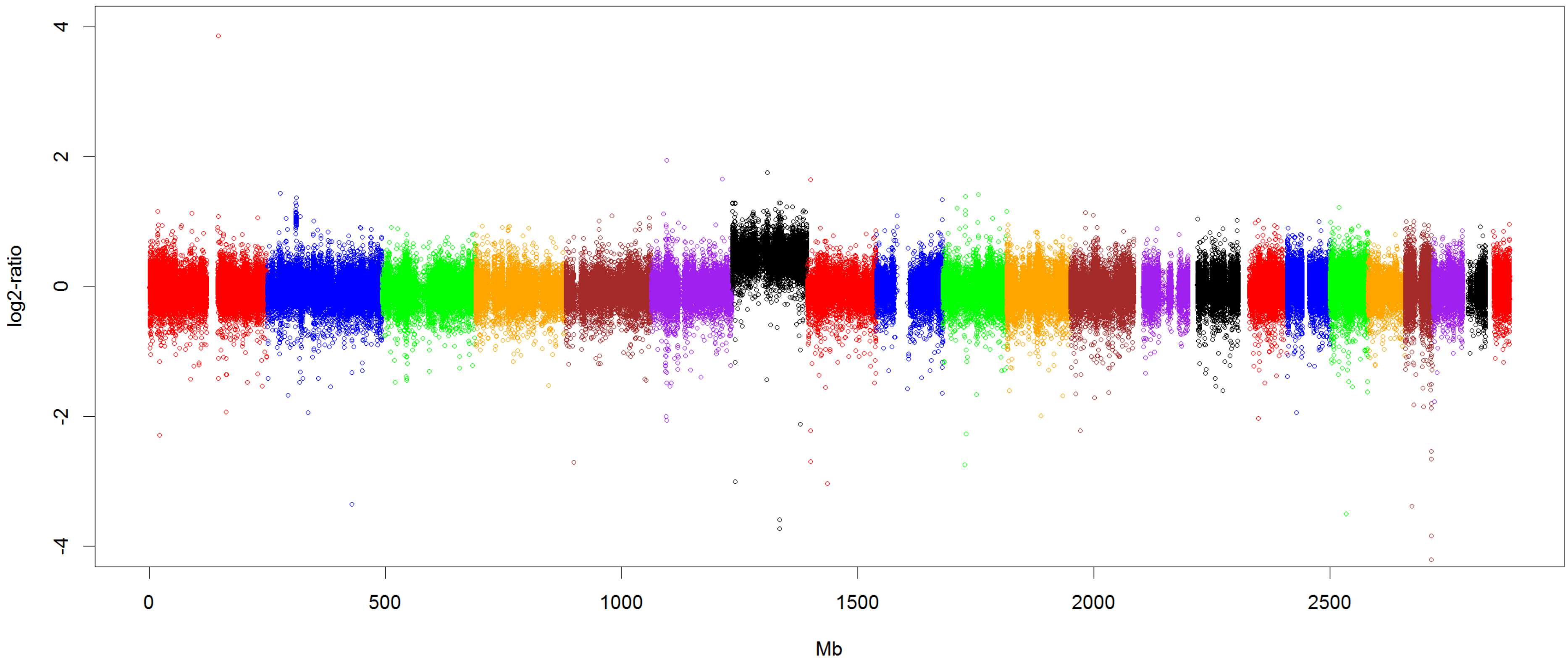

Whole exome CNV of DLBCL-MAYO_DLBCL_3653-Tumor_Tumor

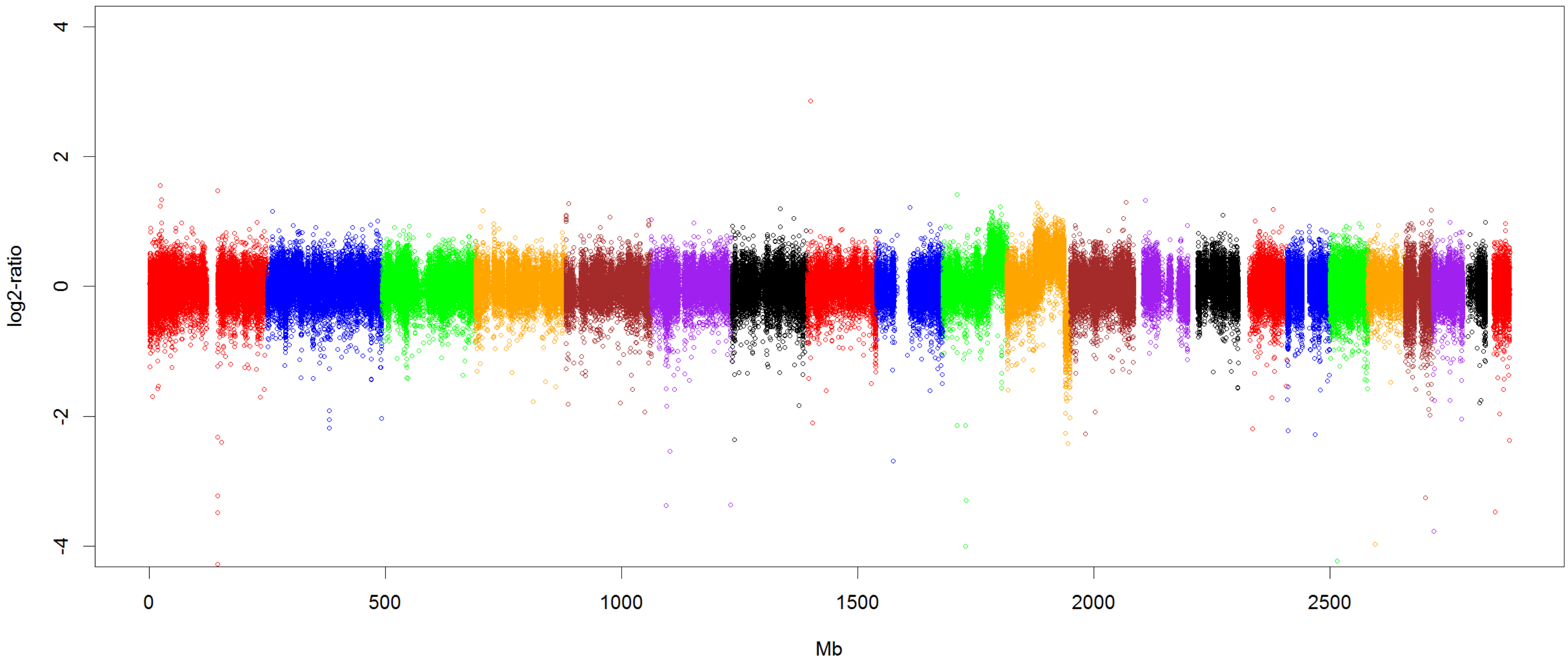

Whole exome CNV of DLBCL-MAYO_DLBCL_5613-Tumor_Tumor

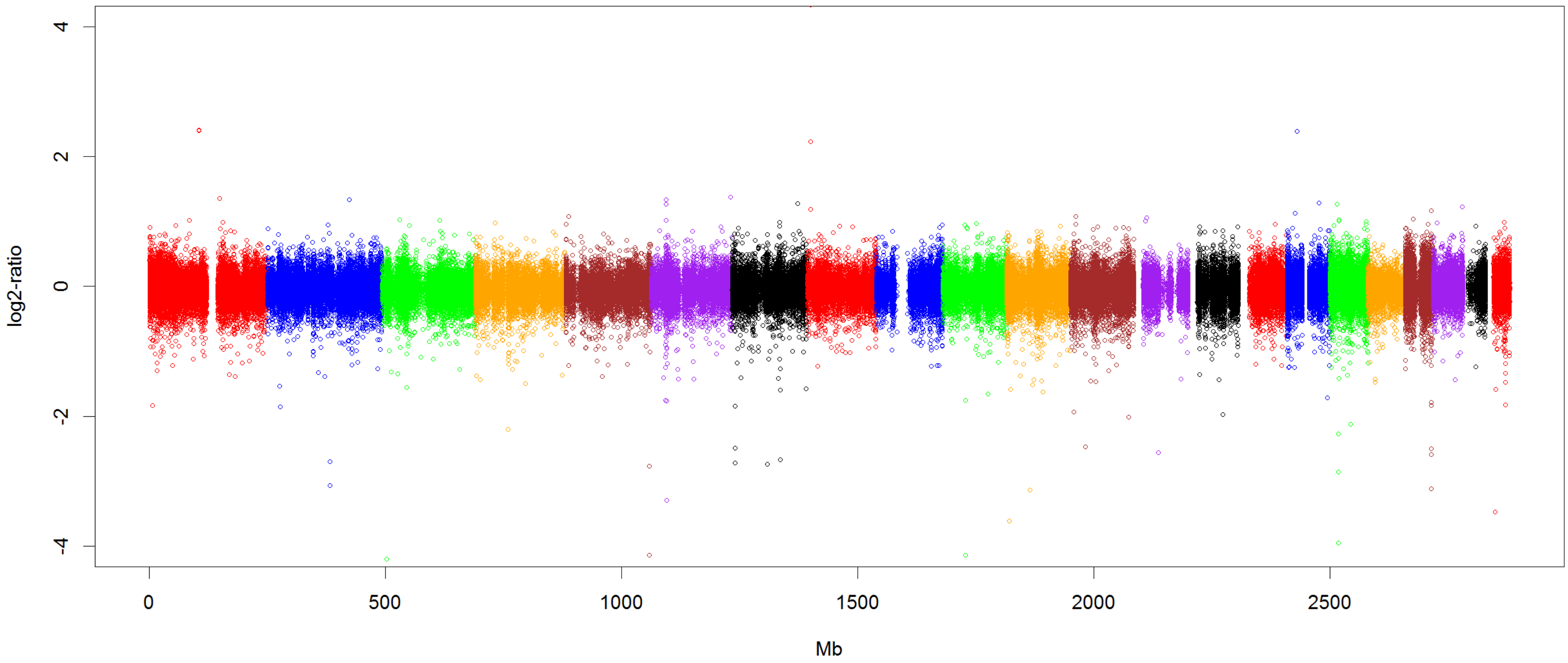

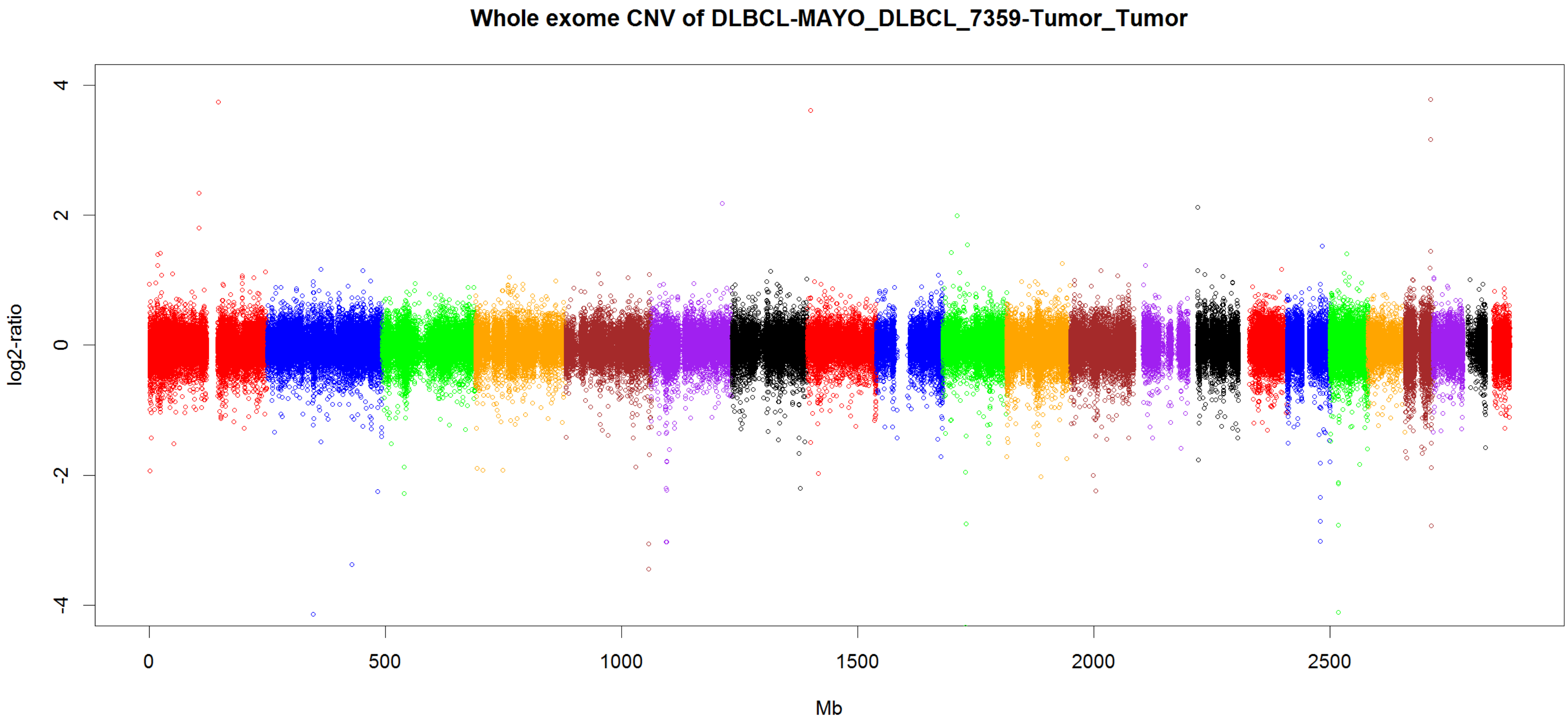

Whole exome CNV of DLBCL-MAYO_DLBCL_7359-Tumor_Tumor
log2-ratio
4
2
0
-2
-4
0
500
1000
1500
2000
2500
Mb
