# Supplementary material for: Whole-exome analysis reveals novel somatic genomic alterations associated with outcome in immunochemotherapy-treated diffuse large B-cell lymphoma
Source: Blood Cancer J. 2015 Aug 28;5(8):e346–. doi: 10.1038/bcj.2015.69 (PMC4558593; doi:10.1038/bcj.2015.69)
Supplement: Supplementary Figure 3 [file bcj201569x4.pdf]

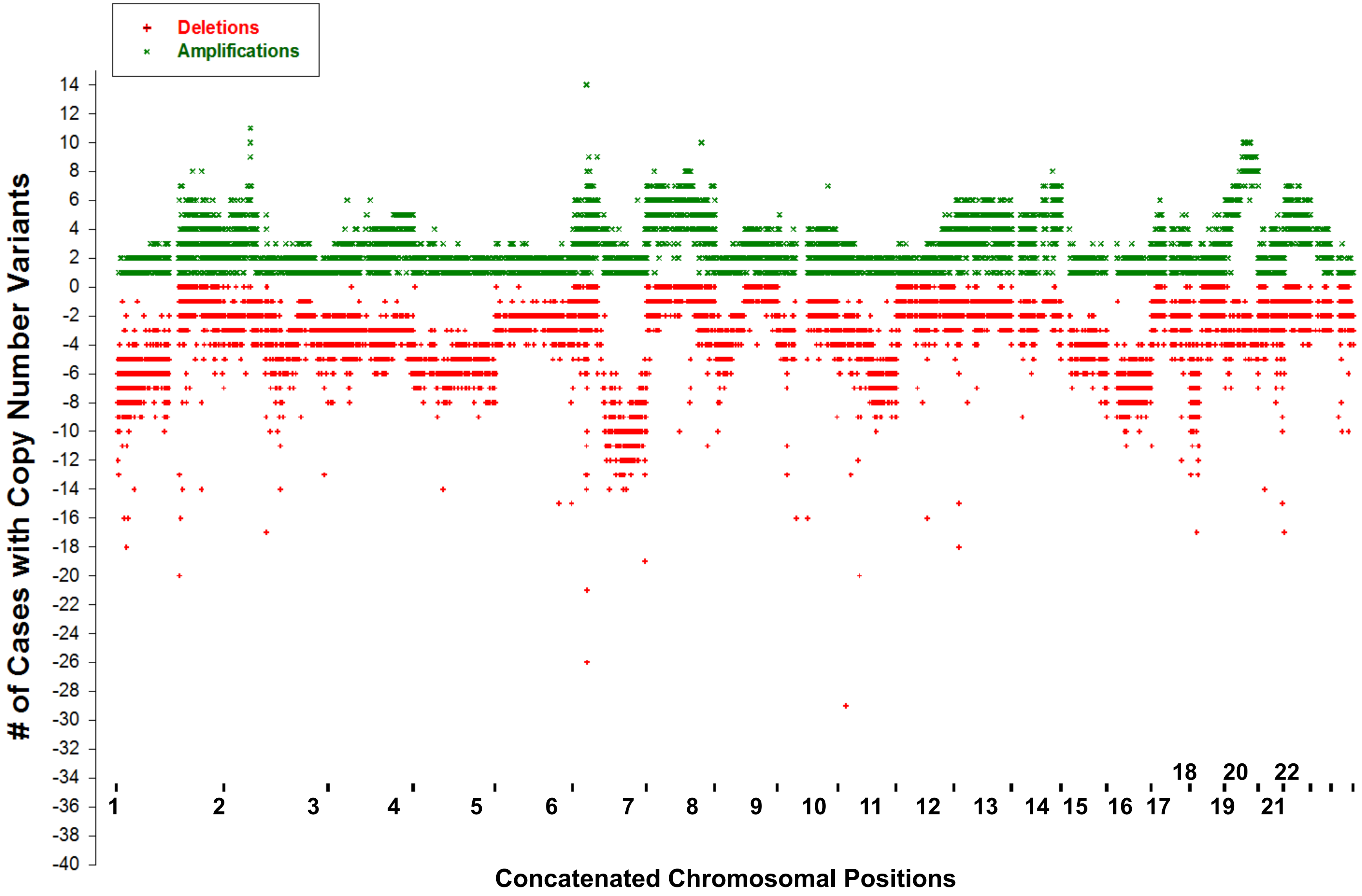

Supplemental Figure 3. Genome-wide view of gene level CNVs. For each gene, the number of DLBCL tumors with amplifications (in green, upper panel) and with deletions (in red, lower panel) were counted and displayed. The X-Axis is the concatenated chromosomal positions from Chr1 to 22 (left to right).
